# Cellular apoptosis susceptibility (CAS) is linked to integrin β1 and required for tumor cell migration and invasion in hepatocellular carcinoma (HCC)

**DOI:** 10.18632/oncotarget.8256

**Published:** 2016-03-23

**Authors:** Juliane Winkler, Stephanie Roessler, Carsten Sticht, Amanda L. DiGuilio, Elisabeth Drucker, Kerstin Holzer, Eva Eiteneuer, Esther Herpel, Kai Breuhahn, Norbert Gretz, Peter Schirmacher, Alessandro Ori, Stephan Singer

**Affiliations:** ^1^ Institute of Pathology, University Hospital Heidelberg, Heidelberg, Germany; ^2^ Medical Research Centre, Medical Faculty Mannheim, University of Heidelberg, Mannheim, Germany; ^3^ Department of Chemistry, Chemical Biology and Biomedical Engineering, Stevens Institute of Technology, Hoboken, NJ, USA; ^4^ Tissue Bank of the National Center for Tumor Diseases (NCT) Heidelberg, Heidelberg, Germany; ^5^ European Molecular Biology Laboratory (EMBL), Structural and Computational Biology Unit, Heidelberg, Germany; ^6^ Leibniz Institute on Aging - Fritz-Lipmann-Institute e.V. (FLI), Jena, Germany

**Keywords:** HCC, CAS, integrin β1, migration, nuclear transport

## Abstract

Importins and exportins represent an integral part of the nucleocytoplasmic transport machinery with fundamental importance for eukaryotic cell function. A variety of malignancies including hepatocellular carcinoma (HCC) show de-regulation of nuclear transport factors such as overexpression of the exportin Cellular Apoptosis Susceptibility (CAS). The functional implications of CAS in hepatocarcinogenesis remain, however, poorly understood. Here we integrated proteomics, transcriptomics and functional assays with patient data to further characterize the role of CAS in HCC. By analyzing ∼ 1700 proteins using quantitative mass spectrometry in HCC cells we found that CAS depletion by RNA*i* leads to de-regulation of integrins, particularly down-regulation of integrin β1. Consistent with this finding, CAS knockdown resulted in substantially reduced migration and invasion of HCC cell lines as analyzed by 2D ‘scratch’ and invasion chamber assays, respectively. Supporting the potential *in vivo* relevance, high expression levels of CAS in HCC tissue samples were associated with macroangioinvasion and poorer patient outcome. Our data suggest a previously unanticipated link between CAS and integrin signaling which correlates with an aggressive HCC phenotype.

## INTRODUCTION

Hepatocellular carcinoma (HCC) is among the most frequent malignant tumors and the second most lethal cancer worldwide with a rising incidence and limited therapeutic options [1, 2]. The only accepted systemic treatment for the advanced disease stages is the multikinase inhibitor sorafenib with a modest survival benefit of less than 3 months [3]. A deeper understanding of the molecular mechanisms that shape the malignant phenotype of HCC cells is critical to identify novel drug targets and will provide the basis for improved therapeutic approaches [4].

The nucleocytoplasmic transport machinery is indispensable to the selective exchange of macromolecules between the nuclear and cytoplasmic compartments [5] and is about to emerge as potential therapeutic target [6]. Nuclear transport receptors are an integral part of the transport system and belong predominantly to the karyopherin protein superfamily [5]. These include importins, exportins, and transportins which shuttle cargos between the nucleus and cytoplasm by passage through the nuclear pore complex (NPC) [7–9]. Classical protein import involves the recognition of a nuclear localization signal (NLS) on a cargo protein by importin-α (imp-α) and subsequent association with importin-β1 to form a trimeric complex [10]. This importin/cargo complex transverses the NPC, dissociates in a RanGTP dependent manner and thus releases its transport substrate into the nucleoplasm [7]. Imp-α is then re-shuttled to the cytoplasm by its exclusive exporter Cellular Apoptosis Susceptibility (CAS, CSE1L, XPO2) [7, 11] and therefore available for subsequent import events. CAS was initially described as an apoptosis susceptibility protein by Brinkmann et al. [12, 13]. However, CAS was also shown to be overexpressed in a variety of solid tumors [14–19] including HCC [20, 21]. Taken together, these data suggest diverse and context-specific functions of CAS that are still incompletely understood.

Integrins are type I transmembrane heterodimeric glycoprotein receptors. They are highly conserved receptors that facilitate cell adhesion and interaction with the extracellular matrix (ECM). Integrin dimers are each composed of 1 of 18 α-subunits and 1 of 8 β-subunits. They have the ability to form at least 24 distinct heterodimers with varied tissue distribution. The extracellular domain of each heterodimer binds proteins of the ECM including collagens (e.g. α1β1, α2β1), fibronectin (e.g. αvβ1, α5β1), and laminin (e.g. α3β1, α6β1), while the intracellular cytoplasmic tail domain is connected to cytoskeletal proteins via cytoplasmic adaptor proteins. This linkage allows integrins to transduce signals bi-directionally through the plasma membrane. Important for out-side-in signal transduction is the formation of the focal adhesion complex which mediates assembly of the actin cytoskeleton and activation of downstream signaling pathways involved in proliferation, survival, and migration. [22].

Here, we describe a previously unanticipated link between CAS and integrin β1. We show that CAS depletion in HCC cells results in a significant down-regulation of integrin β1 and strikingly reduced tumor cell motility. Furthermore, we found CAS and integrin β1 expression to be significantly higher in patients with macroangioinvasion (tumor thrombus in the portal vein) and shorter overall and disease-free survival. This indicates that CAS can be used as a negative prognostic marker in HCC.

## RESULTS

### CAS is linked to integrin β1

**Figure 1:**
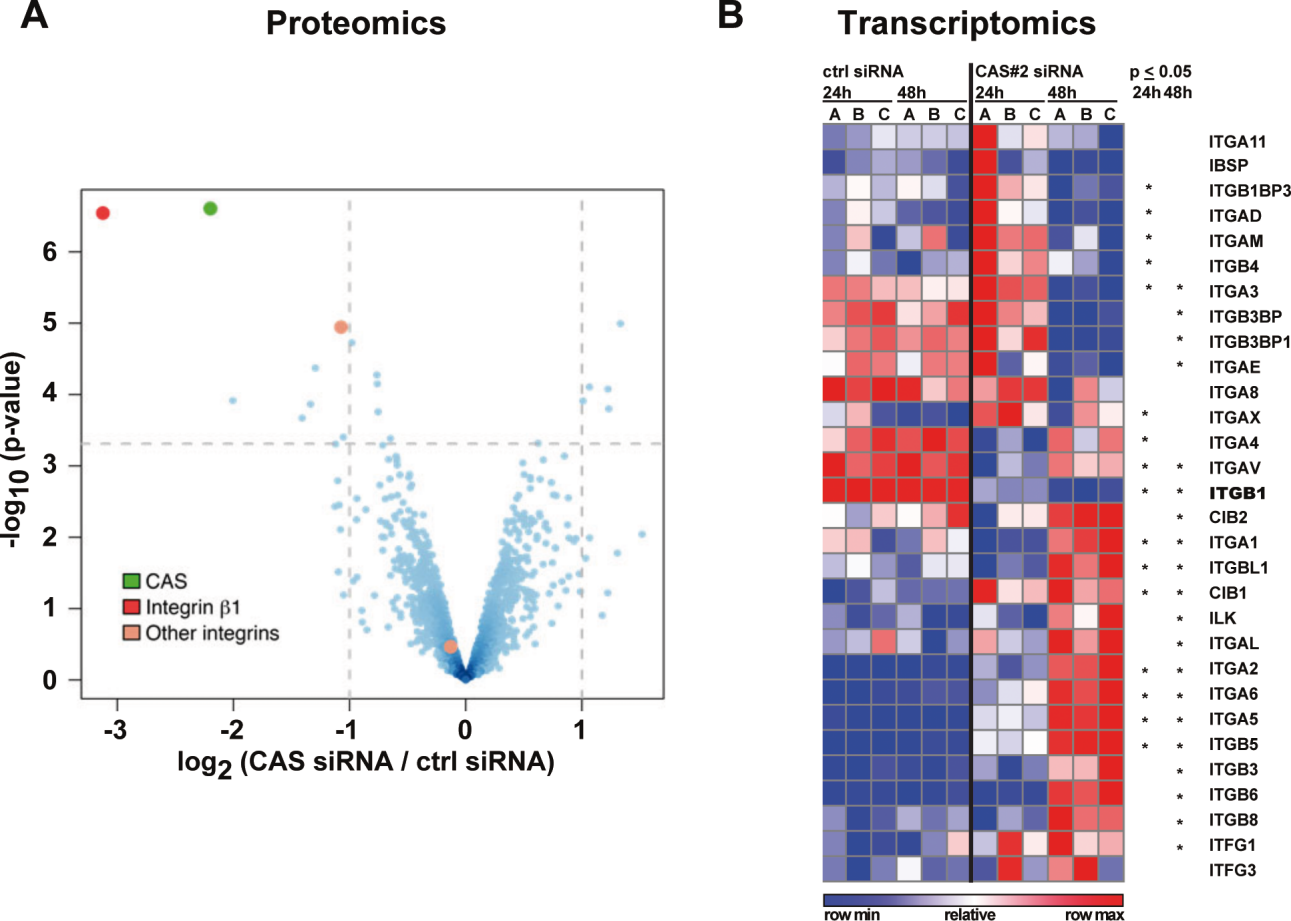
Integrin Δ1 protein and transcript are reduced after CAS silencing (**A**) HLE cells were treated either with a control siRNA (ctrl) or a CAS specific siRNA (CAS#2) for 72 h and analyzed by label-free shotgun proteomics. Vulcano plot depicts significance (*p*-values, -log_10_, y-axis) and protein fold changes (log_2_, x-axis) derived from three biological replicates (red: integrin Δ1, green: CAS, orange: integrin αV (upper dot) and integrin α5 (lower dot)) (**B**) HLE cells were treated as described in (A) either for 24 h or 48 h and subjected to cDNA microarray analyses. Blue and red heat map shows integrins and integrin-associated genes with each replicate relative to the mean expression across all samples. Stars indicate a significant *p*-value (*p* ≤ 0.05).

In an unbiased approach we analyzed global protein changes upon *si*RNA-mediated CAS depletion in HLE cells by label-free quantitative mass spectrometry (qMS). Interestingly, less than 1% of the 1716 measured proteins (listed in Supplementary Table 1) were significantly de-regulated based on an adjusted *p*-value of 0.05 and a fold change of > 2-fold (Figure 1A, upper left quadrant, for validation by immunoblotting see also Supplementary Figure 1B). The most striking change in protein level upon CAS knockdown occurred for integrin β1 (red dot). This factor, a key component in the integrin signaling pathway, was present at levels ∼ 6-fold lower following CAS knockdown. This change in protein abundance was even more dramatic than the knockdown target itself, which exhibited ∼ 4-fold lower levels (green dot). An additional member of the integrin family, integrin αV, was also down-regulated (∼ 2-fold, orange dot). Alternatively, integrin α5 remained unaltered (orange dot, lower middle quadrant). This observation was expanded to the whole integrin family by examination of the dataset from a previously performed genome wide expression profiling after CAS silencing in HLE cells [21]. As illustrated in Figure 1B, we found 25 integrin or integrin-associated genes to be significantly de-regulated 24 and/or 48 h after CAS knockdown (see Supplementary Figure 1A for knockdown efficiency). Consistent with the proteomic data, differential expression of *ITGB1* was observed by a significant decrease 24 h after CAS knockdown and further decrease after 48 h with high consistency between the replicates (for validation by quantitative real-time polymerase chain reaction (qRT-PCR) see Supplementary Figure 1C). *ITGAV* also exhibited reduced expression at both time points; however, had a tendency to recover after 48 h. In contrast, *ITGA2*, *ITGA6*, and *ITGB5* transcripts gradually increased. Meanwhile, other integrins such as *ITGA3* and *ITGA1* showed divergent up-down or down-up expression patterns, respectively. Thus, these data suggest a significant remodeling of integrins in response to CAS knockdown, reflected by a substantial down-regulation of integrin β1 and diverse expression patterns for other integrins.

### CAS is essential for migration and invasion of HCC cells *in vitro*

**Figure 2:**
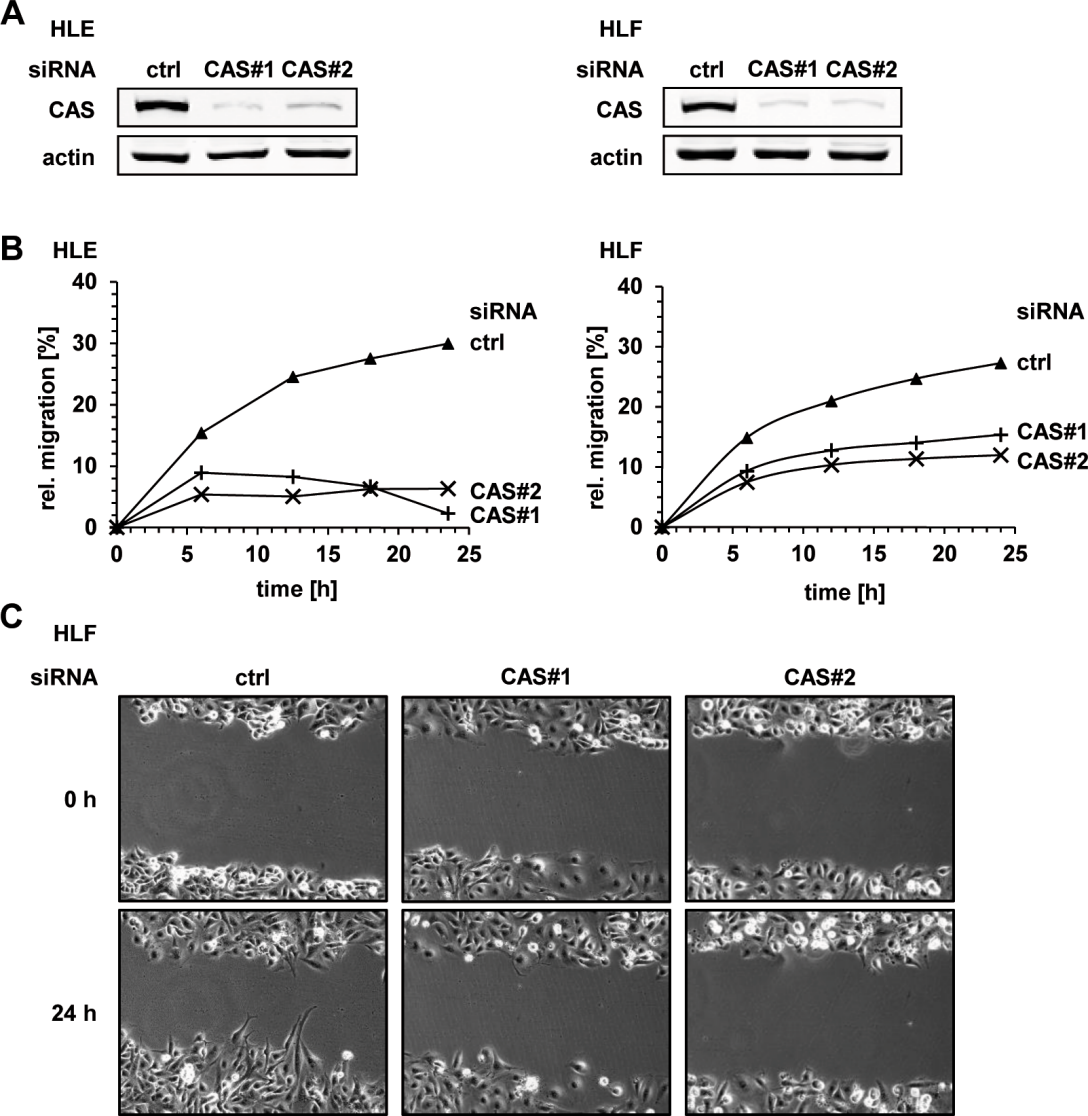
CAS is essential for migration of HCC cells *in vitro* (**A**) HLE and HLF cells were treated either with a control siRNA (ctrl) or two CAS specific siRNAs (CAS#1 and CAS#2) and cell extracts were immunoblotted with the indicated antibodies. (**B**) HLE (left panel) and HLF (right panel) cells were treated as described in (A). 48 h after transfection proliferation was inhibited using mitomycin C and confluent cell monolayer was scratched. Migration was measured by monitoring the closure of the “scratch-wound” using life cell imaging. Time course diagrams show a representative experiment, respectively. (**C**) Corresponding pictures illustrate the migration assay performed in HLF cells.

**Figure 3:**
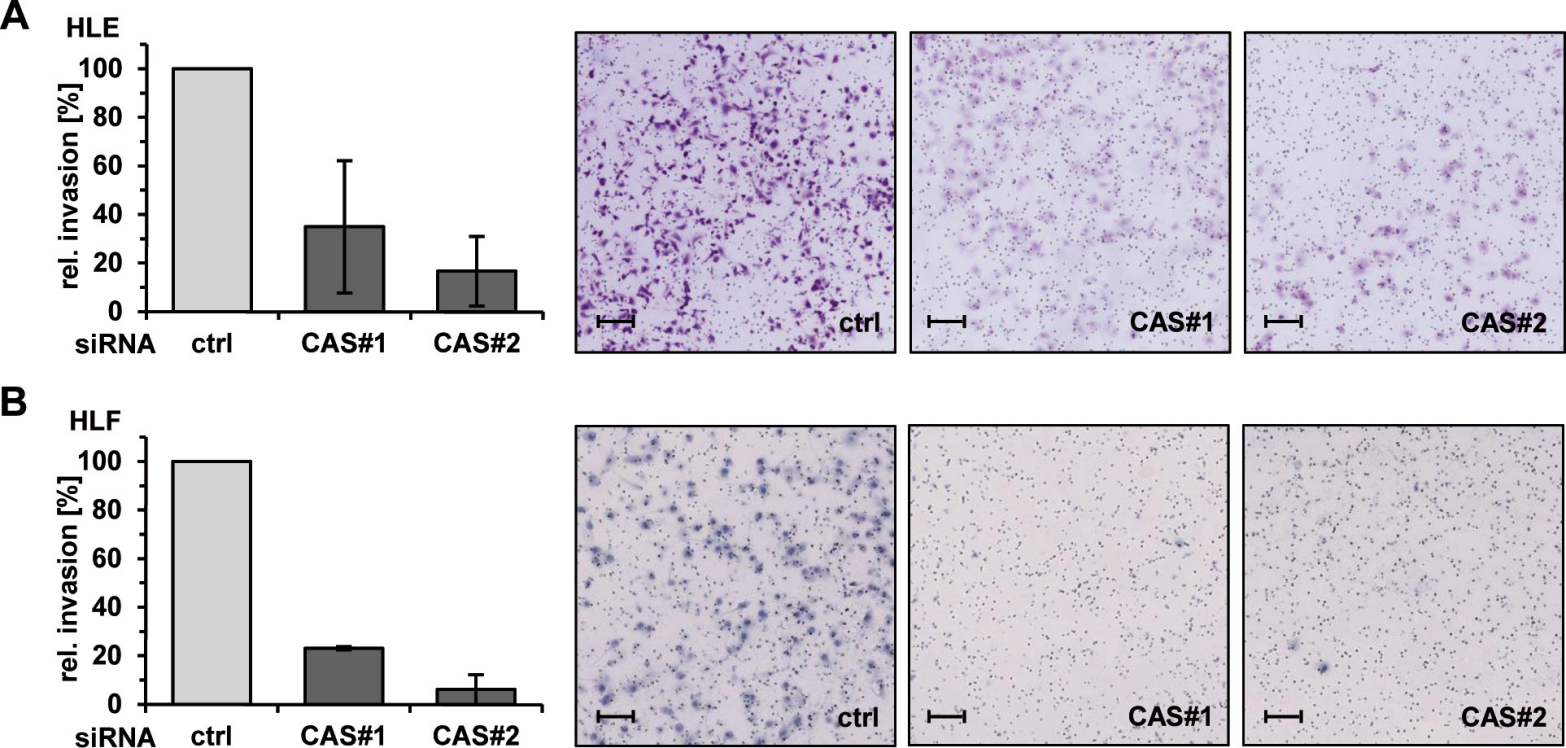
CAS is essential for invasion of HCC cells *in vitro* (**A**) HLE and (**B**) HLF cells were treated as described before and invasion through a matrigel coated membrane was analyzed 48 h after transfection. Invaded cells were stained with crystal-violet (right panel) and quantified (left panel). Data are normalized to the ctrl condition and represented as mean of three (A) or two (B) biological replicates ± standard deviation. scale bars = 100 μm.

Integrin β1 is the predominantly expressed β-subunit in epithelial cells and plays an important role in tumor cell migration and invasion [23]. Thus, we analyzed the effects of CAS depletion on migration and invasion capacity of HCC cells *in vitro*. We performed two-dimensional scratch assays combined with time-lapse microscopy to monitor the gap closure in the respective conditions. The knockdown efficiency of both CAS specific siRNAs (CAS#1 and CAS#2) is illustrated in Figure 2A (see also Supplementary Figure 2A). Consistent with our hypothesis, cell migration of HLE and HLF cells was strongly impaired following CAS silencing. This was reflected by an up to ∼ 5-fold lower gap closure under the respective siRNA conditions (Figure 2B, and Supplementary Figure 2B). Representative images from time-lapse microscopy of HLF cells are shown in Figure 2C (see also Supplementary Figure 2C). Encouraged by these findings, we investigated if CAS is necessary for tumor cell invasion using a transwell-chamber assay. The invasion capacity of HCC cells (HLE) was shown to be strikingly reduced upon CAS depletion (Figure 3A). Cells subjected to CAS knockdown invaded the matrigel up to 10 times less when compared to controls. A similar loss of invasion capacity was shown for HLF cells and is depicted in Figure 3B. To test if the impact of CAS on tumor cell motility is indeed related to integrin Δ1, we performed similar experiments after integrin Δ1 depletion in HLE cells (Supplementary Figure 3A). Supporting our hypothesis absence of integrin Δ1 led to a diminished migratory (Supplementary Figure 3B) and invasion capability (Supplementary Figure 3C) of HLE cells. In line with the observation that CAS is required to maintain integrin β1 expression, we conclude that CAS is essential for tumor cell migration and invasion in HCC cells.

### CAS is a negative prognostic marker in HCC

**Figure 4:**
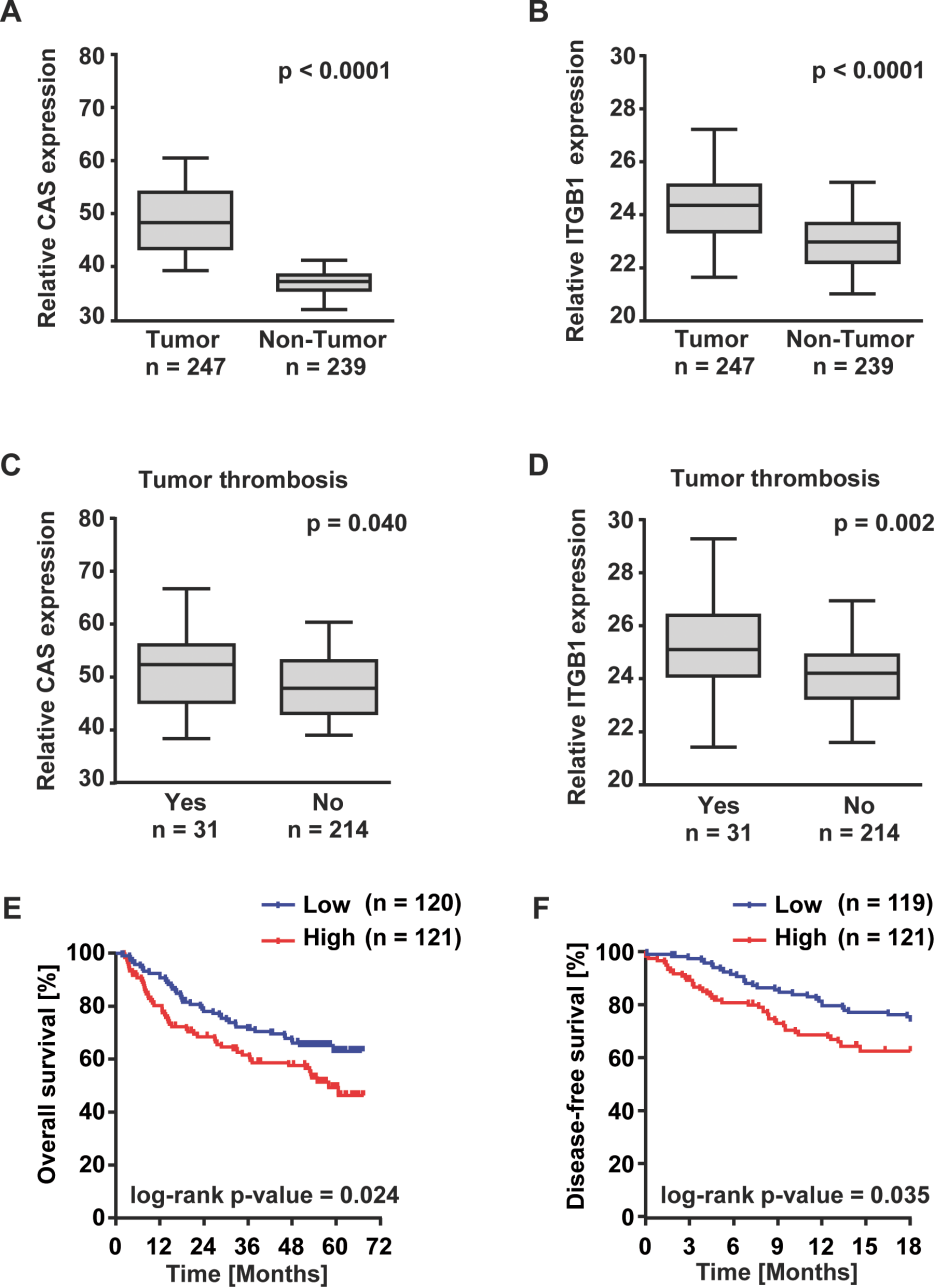
High expression of CAS is associated with poor patient outcome Shown are (**A**) CAS and (**B**) integrin Δ1 (ITGB1) gene expression in HCC (*n* = 247) compared to non-tumorous liver tissue (*n* = 239). Differential gene expression of (**C**) CAS and (**D**) ITGB1 in HCCs with (*n* = 31) or without (*n* = 214) a tumor thrombus in the portal vein. Information on tumor thrombosis was available for 245 patients. (**E**) Kaplan–Meier plots illustrate 6-year overall survival and (**F**) disease-free survival over 18 months of low (blue) and high (red) CAS expressing HCC patients. Cutoff value for low and high expression of CAS was the median CAS expression of all patients. Information on overall and disease-free survival were available for 241 and 240 patients, respectively.

These observations together with previously published reports [20, 21] suggest that CAS may be associated with an aggressive phenotype of liver cancer. Therefore, we examined the prognostic relevance of CAS expression in a large HCC patient cohort (*n* = 247). Consistent with our *in vitro* data, HCC tumor tissues are characterized by a higher median expression of CAS (Figure 4A) and integrin β1 (Figure 4B) compared to adjacent non-tumor tissue. Furthermore, a positive correlation of CAS and integrin Δ1 immunohistochemistry (IHC) scores (*r* = 0.43; *p* < 0.01; see Supplementary Figure 4A) was observed in another HCC cohort (*n* = 91) from which additional tissue specimen were available. Representative pictures of CAS and integrin Δ1 IHC stainings are shown in Supplementary Figure 4B.

Moreover, particularly relevant to the pro-migration and pro-invasion phenotype of CAS, HCC patients with macroangioinvasion (tumor thrombus in the portal vein) showed significantly higher CAS and integrin β1 expression (Figure 4C and 4D). Finally, the patient cohort was dichotomized based on the median value of CAS expression. Overall survival over 72 months as well as the disease-free survival over 18 months was significantly lower in HCC patients with high levels of CAS transcripts (Figure 4E and 4F).

Collectively, these data demonstrate that high expression of CAS is associated with a more aggressive course of the disease and a poorer outcome of HCC patients.

## DISCUSSION

In this study, we show that CAS is linked to integrin expression in HCC, particularly to integrin β1, and is essential for tumor cell motility in HCC cell lines. The preferentially expressed integrins in neoplastic liver cells include α1β1, α2β1, α3β1, and α6β1 [24] underscoring the importance of the β1 subunit in this particular cell type. The fundamental role of the β1 subunit for remodeling of the cytoskeleton [23] and activation of pro-migratory downstream targets [25] is highlighted by the embryonic lethal phenotype of integrin β1 knockout mice [26], by a reduced migration and invasion capacity in diverse solid tumors and an overall delayed tumor progression and metastatic spread upon integrin β1 knockdown/knockout [27–29]. In light of these studies the migration and invasion defect observed upon CAS depletion in HCC cells might be explained by down-regulation of integrin β1. However, the functional implications of up-regulated integrin α1, α6, and β5 transcripts in this scenario are less obvious and may reflect a compensatory response to integrin β1 reduction. This speculation is difficult to confirm since the integrin-subunit manifestations at protein level were below the detection limit of this qMS approach. As shown for integrin α5, a steadily increasing integrin transcript level upon CAS depletion does not necessarily reflect protein level changes. Furthermore, integrin-independent effects of CAS, for instance on cytoskeleton reorganization, require consideration. CAS has been described to interact directly with microtubules and to promote protrusion extension in breast cancer cells [30], which may also be relevant in HCC cells. Moreover, we cannot entirely rule out that a minor portion of the dramatic effects on tumor cell motility may result from a reduced overall fitness of the tumor cells after CAS knockdown. However, based on previously performed time course experiments [21], we do not expect a substantial impact on tumor cell survival 2 days after CAS depletion when functional assays were conducted. Finally, it is conceivable that a mislocalization of proteins, without a global change in protein level following CAS depletion, at least partially contributes to the described phenotype. Although label-free shotgun proteomics is a very powerful technique the resolution is limited. This may also explain why apoptosis-related proteins so tightly connected to CAS function, such as RASSF1 (Ras-associated domain family 1 gene product) [31] and XIAP (X-linked inhibitor of apoptosis) [21], did not emerge in our qMS analysis.

Despite these limitations, it is intriguing to speculate about possible direct and indirect mechanisms involved in CAS-dependent integrin β1 regulation. One scenario is the direct chromatin interaction of CAS with the promoter region of *ITGB1* and thereby the direct regulation of integrin β1 expression, similar to the mechanism by which CAS is known to regulate a subset of p53 target genes [32]. Another mechanism could involve transcription factors (TFs) driving integrin β1 expression that require imp-α for their nuclear import. Among the various imp-α isoforms imp-α1 appears of particular interest in this context, because it was one of the few transport factors including CAS we previously observed to be overexpressed in HCC tissue samples compared to non-tumorous liver [21]. Furthermore, in the aforementioned study we could link the pro-survival function of CAS in HLE and HLF cells to imp-α1. This assumption is further supported by a substantial nuclear accumulation of imp-α1 upon CAS silencing in HCC *in vitro* (Supplementary Figure 5). This finding may suggest that the regulation of integrin β1 and the functional effects of CAS on tumor cell motility are connected to imp-α1. In this perspective, TFs requiring imp-α1 for their nuclear import would be retained in the cytoplasm resulting in lowered expression of integrin β1. Obvious candidates would include TFs of the HGF-signaling cascade, e.g. AP1 (Activator protein 1), Erk1/2 (Extracellular-signal regulated kinase), and STAT3 (Signal transducer and activator of transcription 3), that were shown to up-regulate integrin β1 [33]. However, according to previous reports not specifically analyzing HCC cells, none of these TFs seem to rely on imp-α1. STAT3 was reported to be dependent on imp-α5 and imp-α7 [34], Erk1/2 requires imp-α7 [35] and AP1 is completely independent of imp-α [36]. Comprehensive, cell-specific proteomic studies investigating TFs and transcriptional co-regulators exclusively relying on imp-α1-dependent import appear crucial in this context, but are, to the best of our knowledge, lacking. Furthermore, we cannot rule out other imp-α isoforms possibly involved in the regulation of integrin Δ1. Finally, the strong decrease of integrin β1 protein may also hint to an additional, post-transcriptional level of regulation (e.g. translation or protein stability) possibly supported by the fact that CAS can be localized in the cytoplasm. Dissecting the detailed mechanism(s) by which CAS regulates integrin β1 will be a rewarding subject for future studies.

The importance of the nucleocytoplasmic transport machinery as a potential target in cancer therapy is supported by a previously described global enhancement of nuclear transport upon malignant transformation [37] and already exemplified by specific inhibitors of the nuclear export protein Crm1 (Chromosome region maintenance 1 protein homolog). Selective Inhibitors of Nuclear Export (SINE), like Selinexor, already entered Phase I/II of clinical trials for solid and hematological malignancies [38]. The expression profile of CAS in HCC patient samples and its functional effects in HCC cell lines, as presented here and in our previous study [21], may designate CAS as a promising therapeutic target. This hypothesis is also supported by the observation that CAS depletion does not significantly affect the viability of non-tumorigenic liver cells (THLE-2, Supplementary Figure 6A) and does not induce apoptotic cell death in these cells (see Supplementary Figure 6B and 6C). These data indicate tumor-specific effects of CAS and are encouraging, but require further validation experiments including appropriate *in vivo* models. Given that similar characteristics, such as overexpression, pro-tumorigenic functions, and association with a poor prognosis, were also described for imp-α1 [21, 39], an important transport substrate of CAS in HCC, a potential therapeutic strategy may involve the disruption of the CAS/imp-α1 transport cycle. Thus, blocking the interaction of CAS and imp-α1 with a compound could be a promising approach to improve the treatment of HCC patients in the future.

## MATERIALS AND METHODS

### Tissue culture

The human hepatoma cell lines HLE (JCRB0404; Osaka, Japan) and HLF (JCRB0405; Osaka, Japan), established *in vitro* from the hepatocellular carcinoma of a 68-year-old patient from Dor et al. [40], were cultured with Dulbecco's modified Eagle's medium (DMEM, obtained from PAA Laboratories, Cölbe, Germany) in an atmosphere containing 5% CO_2_. Medium was supplemented with 10% fetal calf serum (FCS) and 1% penicillin/streptomycin (Sigma, München, Germany). SV40-T-antigen-immortalized human liver epithelial cells (THLE-2) [41] were cultivated with BEBM Basal Medium supplemented with BEGM SingleQuot Kit (obtained from Lonza, Walkersville, MD, USA), except Gentamycin/Amphotericin and Epinephrine. Additionally, the medium for THLE-2 cells was supplemented with EGF (Epidermal growth factor, 5 ng/ml), phosphoethanolamine (70 ng/ml) and 10% FCS. THLE-2 cells were cultivated in cell+ plastic culture bottles and dishes purchased from Sarstedt (Nümbrecht, Germany).

### siRNA-transfections

Target-specific small interfering RNAs (siRNAs) CAS#1 (5′-GGAACUCAGCGAUGCAAAU-3′) and CAS#2 (5′-CAGGAUAAUGUUAUCAAAGUA-3′) were purchased from Eurofins MWG Operon (Ebersberg, Germany). Integrin Δ1#1 (5′-ACAGATGAAGTTAACAGTGAA-3′) and integrin Δ1#2 (5′-TTGCAGTTATGCAGAATCCAA-3′) were obtained from QIAGEN (Hilden, Germany) and the QIAGEN All-Stars duplex served as negative control siRNA for all knockdown experiments. The transfections were performed according to the manufacturer's instructions using Oligofectamine (Invitrogen, Karlsruhe, Germany) with a final siRNA concentration of 50 nM. Knockdown efficiency was verified for CAS specific siRNAs 24 h after transfection (Supplementary Figure 1A).

### Immunoblotting

Immunoblotting was performed as previously described [21]. In brief, whole protein lysates were separated by SDS/PAGE and transferred to nitrocellulose membranes (Whatman, Dassel, Germany). Membranes were incubated with the following primary antibodies diluted in 5% Milk/TBST-containing blocking solution: mouse monoclonal anti-actin (1:10000, 691391, MP Biomedicals, Illkirch, France), anti-CAS mouse monoclonal antibody (1:500, ab54674, Abcam, Cambridge, UK), rabbit monoclonal antibodies of anti-integrin Δ1 (1:2 000, ab179471, Abcam), anti-integrin αV (1:2 000, ab124968, Abcam), and anti-HO-1 (1:20 000, ab68477, Abcam). Detection was performed using Odysee Sa Infrared Imaging System (LI-COR Bioscience, Bad Homburg, Germany).

### Immunofluorescence (IF) microscopy

IF staining was performed as previously described [42]. In brief, HLE and HLF cells grown on coverslips were fixated with ice-cold methanol for 5 min and acetone for 30 sec and incubated with anti-imp-α1 rabbit monoclonal antibody for 1 h at RT (1:50, ab84440, Abcam, Cambridge, UK) and incubated with the corresponding secondary antibody (anti-rabbit MFP488 IgG, 1:200, MFP-A1034, MoBiTec, Göttingen, Germany).

### Migration and invasion assay

Tumor cell migration was measured in a two-dimensional “scratch” assay following two days of siRNA-mediated knockdown. To repress proliferation cells were treated with mitomycin C (5 μg/mL) for 3 h before the cell monolayer was scratched with a pipette tip. Cells were then incubated with hepatocyte growth factor (HGF, 10 ng/mL) for 24 h to induce migration. Scratches were monitored using the Olympus Cell^R Live Cell Imaging System with an IX81 motorized inverted microscope and a Hamamatsu camera, fitted with a climate chamber. Images were acquired using the Olympus excellence RT software (Olympus, Hamburg, Germany). Relative migratory capacity was determined by calculating the percentage of the cell-free area.

Tumor cell invasion was analyzed by using BD Biocoat Matrigel invasion chambers (BD Bioscience, Heidelberg, Germany). 48 h after transient transfection with the described siRNAs cells were transferred in membrane coated inserts suspended in FCS free medium (25 000 cells/insert). Cells were stimulated to pass through the membrane into the subjacent matrix attracted by 10% FCS containing medium from the bottom of the inserts. After 24 h, cells that passed through the matrigel reaching the lower part of the membranes were fixed with 4% formalin and stained with crystal violet (0.5% (w/v) and 10% (v/v) methanol). Membranes were digitally imaged and invaded cells were counted visually.

### Multiplex analysis of cell death and cell viability

In order to determine cell viability and cell death, the CellTox Green cytotoxicity assay was multiplexed with the CellTiter-Blue cell viability assay (both Promega, Mannheim, Germany). First, the amount of dead cells was quantified using Cell Tox Glow solution (1:5 dilution according to manufacturer's instructions) with FLUOSTAR Omega microplate reader using the filter combination excitation: 485 nm, emission: 520 nm. Subsequently, Cell Titer Blue was applied in a 1:5 dilution to the same wells. After incubation of 1 h, metabolic activity was measured using the filter combination excitation: 540 nm, emission: 610 nm.

### Proteomic analyses

Mass spectrometry was conducted as recently described [21]. In brief, lysates isolated from HLE cells three days upon siRNA treatment were processed and analyzed in triplicates by liquid chromatography-tandem mass spectrometry (LC-MS/MS). Peptides were assessed using a nano-Acquity UPLC system (Waters, Eschborn, Germany) connected online to a LTQ-Orbitrap Velos Pro instrument (Thermo Fisher Scientific, Bremen, Germany).

### Gene expression analysis

Investigation of integrin gene expression levels upon CAS knockdown was based on a dataset from a previously performed Affymetrix mRNA Microarray analysis [21]. Validation of integrin Δ1 gene expression was conducted as previously described [21] using qRT-PCR and ITGB1 primers (forward: AAGCGAAGGCATCCCTGAAA, reverse: GTCTACCAACACGCCCTTCA) purchased from Apara Bioscience (Denzlingen, Germany).

### Human liver samples

Our study used a published Affymetrix U133A2.0 gene expression data set derived from 247 HCC patients (90.7% HBV positive) as described by Roessler et al. [43] (Gene Expression Omnibus accession number GSE14520). Patient samples of this data set were obtained with informed consent from patients at the Liver Cancer Institute (LCI) and Zhongshan Hospital (Fudan University, Shanghai, China). Immunohistochemical (IHC) staining of a HCC tissue microarray (*n* = 91) was performed by the Tissue Bank of the National Center for Tumor Diseases (NCT) Heidelberg. The use of the samples was approved by the local Ethics Committee. IHC staining was performed using an anti-CAS mouse monoclonal antibody (1:50, ab54674, Abcam, Cambridge, UK) and an anti-integrin Δ1 rabbit monoclonal antibody (1:1 000, ab179471, Abcam) in an automated immunostaining instrument (BenchMark ULTRA IHC/ISH Staining module, Ventana Medical Systems, Tucson, USA). The OptiView DAB IHC Detection Kit (OptiView, Ventana Medical Systems) was used based on the manufacturer's protocol including the following steps: 4 min deparaffination at 62°C, rinsing with EZ Prep, incubation with Cell Conditioner No. 1 for 64 min at 90°C, followed by an incubation with the primary antibody for 24 min at 36°C, 4 min exposure to Optiview Peroxidase Inhibitor, 12 min incubation with Optiview HQ Universal Linker, 12 min treatment in Optiview HRP Multimer, 8 min incubation with a mixture of Optiview H_2_O_2_ and DAB, 4 min exposure to Optiview copper, counterstaining with Haematoxylin for 12 min, and finally an incubation with Bluing Reagent for 4 min. Each incubation was followed by multiple rinsing steps in reaction buffer. The procedure for dehydration of each TMA slide was as follows: 5 min 70% ethanol, 5 min 96% ethanol, followed by two washing steps with 100% ethanol 5 min, and finally 5 min Xylene using the Leica autostainer XL. Finally, the slides were mounted with cover slips (Leica CV5030). IHC scoring was calculated as described before [21].

### Statistical analysis and software

Data are presented as the mean ± standard deviation (SD) of three independent experiments except otherwise specified. Expression differences between HCC and non-tumorous liver samples were assessed by nonparametric Mann-Whitney *U* tests. Overall and disease-free survival of 242 patients was available and analyzed by Kaplan-Meier curves and log-rank *p*-values using GraphPad Prism 6 (GraphPad Software, Inc, La Jolla, CA, USA). The statistical significance was defined as *p* < 0.05.

## SUPPLEMENTARY MATERIALS FIGURES AND TABLE



## Abbreviations

AP1: Activator protein 1
CAS: Cellular apoptosis susceptibility
Crm1: Chromosome region maintenance 1 protein homolog
DMEM: Dulbecco's modified Eagle's medium
FCS: Fetal calf serum
HCC: Hepatocellular carcinoma
HGF: Hepatocyte growth factor
IHC: Immunohistochemistry
Imp-α1: Importin-α1
Imp-α5: Importin-α5
Imp-α7: Importin-α7
ECM: Extracellular matrix
EGF: Epidermal growth factor
Erk1/2: Extracellular-signal regulated kinase
LC-MS/MS: Liquid chromatography-tandem mass spectrometry
qMS: Quantitative mass spectrometry
qRT-PCR: Quantitative real-time polymerase chain reaction
NLS: Nuclear localization signal
NPC: Nuclear pore complex
RASSF1A: Ras-associated domain family 1 gene product
siRNA: Short interfering RNA
SD: Standard deviation
SINE: Selective Inhibitors of Nuclear Export
STAT3: Signal transducer and activator of transcription 3
TF: Transcription factor
XIAP: X-linked inhibitor of apoptosis.
