# Supplementary material for: Cellular apoptosis susceptibility (CAS) is linked to integrin β1 and required for tumor cell migration and invasion in hepatocellular carcinoma (HCC)
Source: Oncotarget. 2016 Mar 23;7(16):22883–92. doi: 10.18632/oncotarget.8256 (PMC5008409; doi:10.18632/oncotarget.8256)
Supplement: Supplementary file 1 [file oncotarget-07-22883-s001.pdf]

Cellular apoptosis susceptibility (CAS) is linked to integrin β1 and required for tumor cell migration and invasion in hepatocellular carcinoma (HCC)

Supplementary Materials

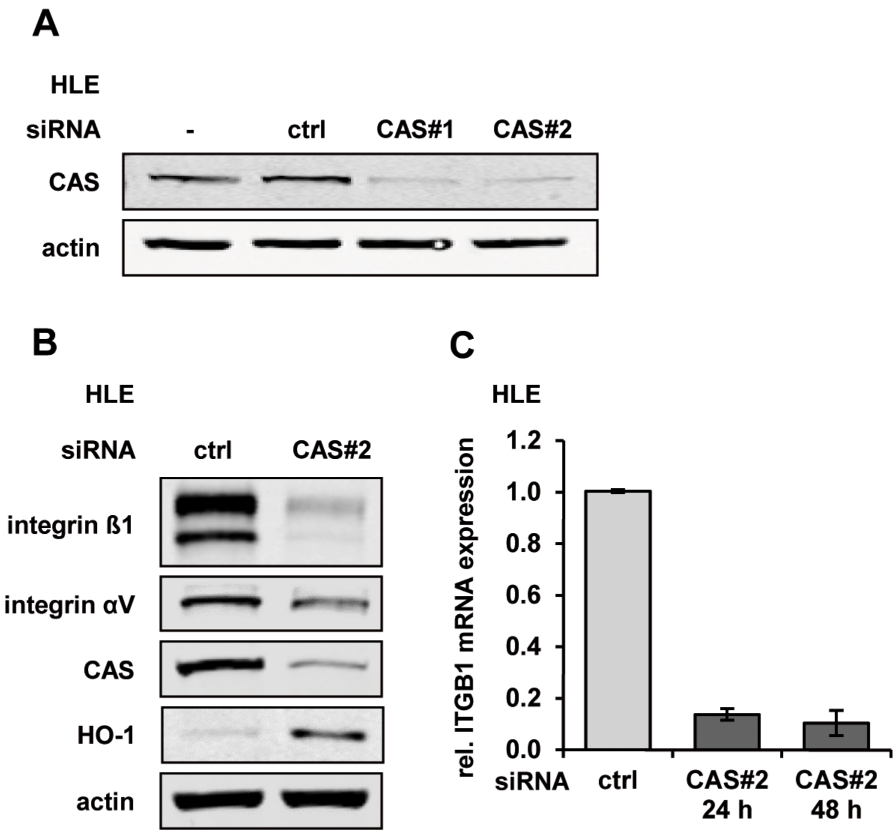

Supplementary Figure S1: Integrin ß1 protein and transcript are reduced after CAS silencing. (**A**) HLE cells were treated either with a control siRNA (ctrl) or two different CAS specific siRNAs (CAS#1 and #2) for 24 h including an untreated condition. HLE cell extracts were immunoblotted for CAS and ß-actin. (**B**) HLE cells were treated either with a control siRNA (ctrl) or a CAS specific siRNA (CAS#2) for 24 h. HLE cell extracts were immunoblotted for CAS, integrin ß1, integrin αV, Heme oxygenase 1 (HO-1) and ß-actin. (**C**) HLE cells were treated as described in (A) for 24 or 48 h, respectively. Bar diagram shows integrin ß1 (ITGB1) transcript level analyzed by qRT-PCR.

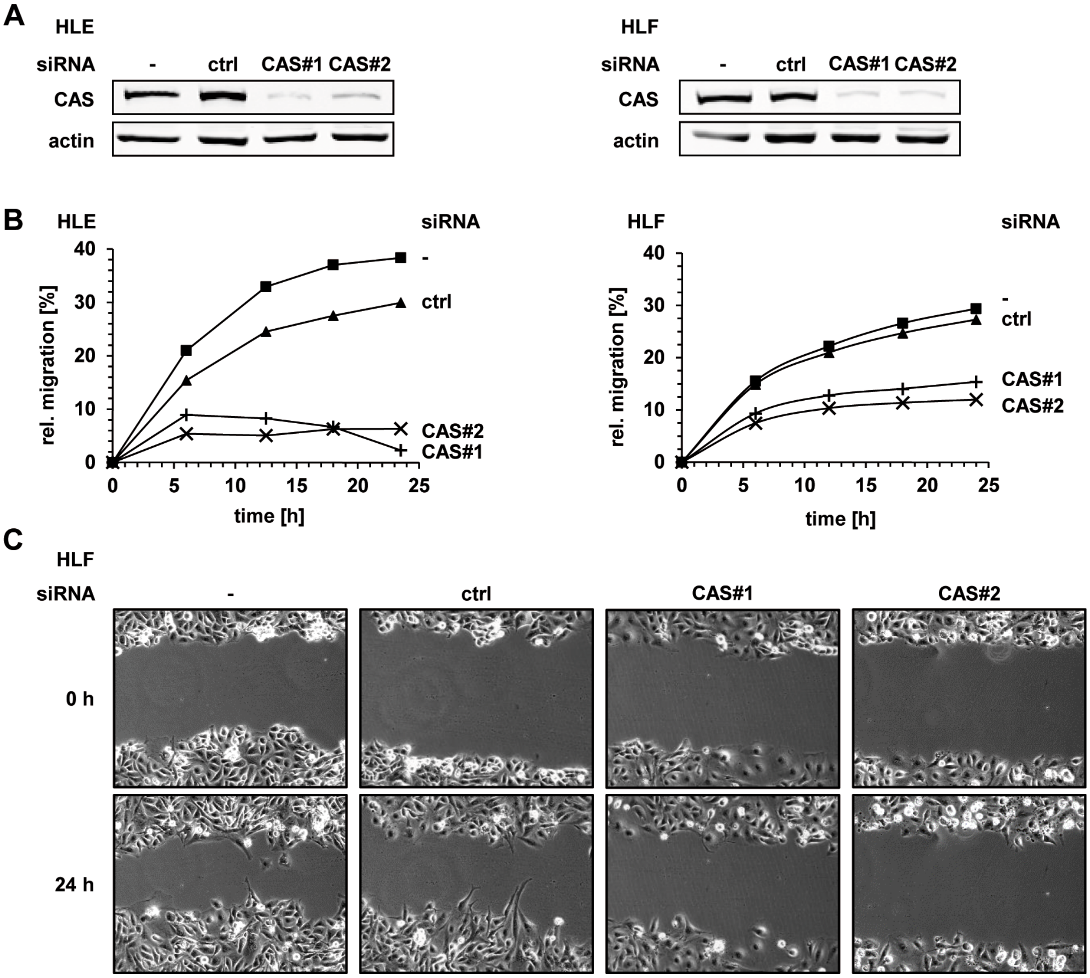

Supplementary Figure S2: CAS is essential for migration of HCC cells *in vitro*. (**A**) HLE and HLF cells were treated either with a control siRNA (ctrl) or two CAS specific siRNAs (CAS#1 and CAS#2) including an untreated condition and cell extracts were immunoblotted with the indicated antibodies. (**B**) HLE (left panel) and HLF (right panels) cells were treated as described in (A). 48 h after transfection proliferation was inhibited using mitomycin C and confluent cell monolayer was scratched. Migration was measured by monitoring the closure of the "scratch-wound" using live cell imaging. Time course diagram shows a representative experiment. (**C**) Corresponding pictures illustrate the migration assay performed in HLF cells.

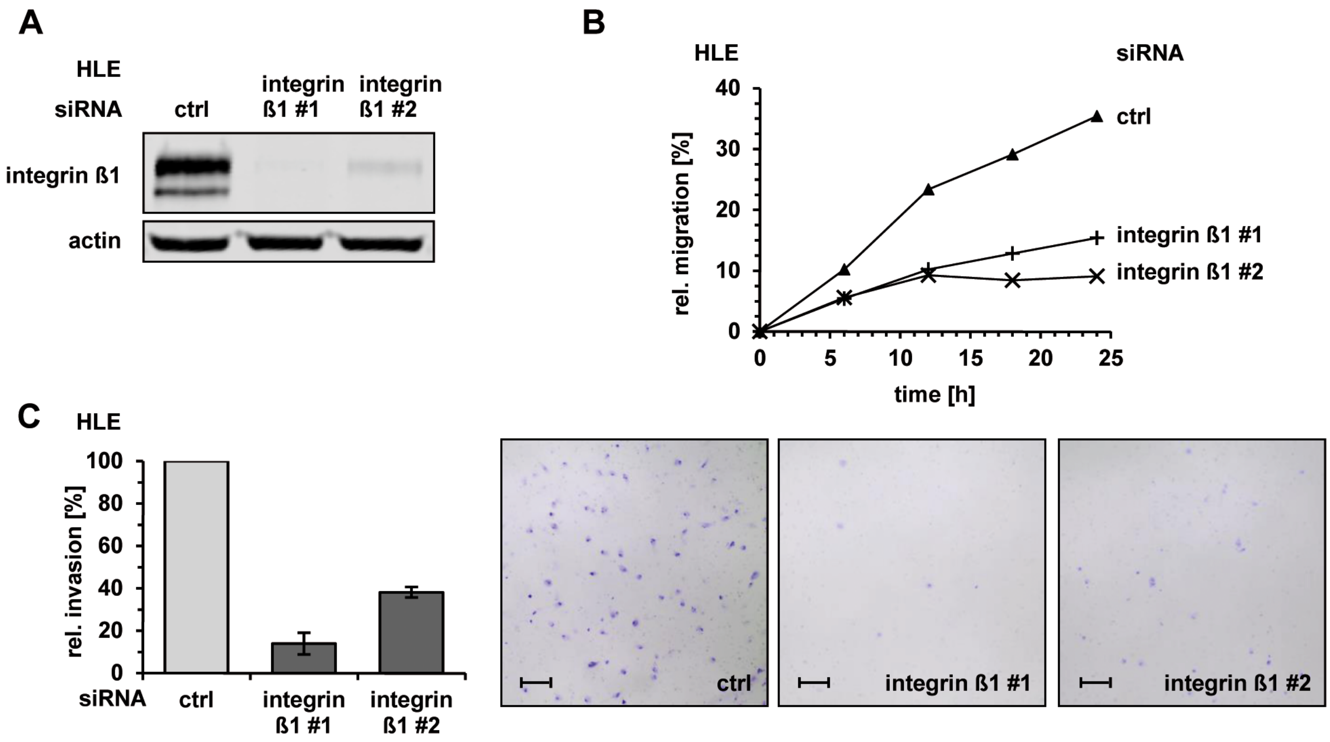

Supplementary Figure S3: Integrin ß1 is essential for migration and invasion of HCC cells *in vitro*. (**A**) HLE cells were treated either with a control siRNA (ctrl) or two integrin ß1 specific siRNAs (integrin ß1#1 and integrin ß1#2) and cell extracts were immunoblotted with the indicated antibodies. (**B**) HLE cells were treated as described in (A). Migration assays were performed as described in Figure 2. Time course diagram shows a representative experiment. (**C**) Invasion ability of HLE cells was analyzed as described in Figure 3. Invaded cells were stained with crystal-violet (right panel) and quantified (left panel). Data are normalized to the ctrl condition and represented as mean of two biological replicates ± standard deviation. scale bars = 100 µm.

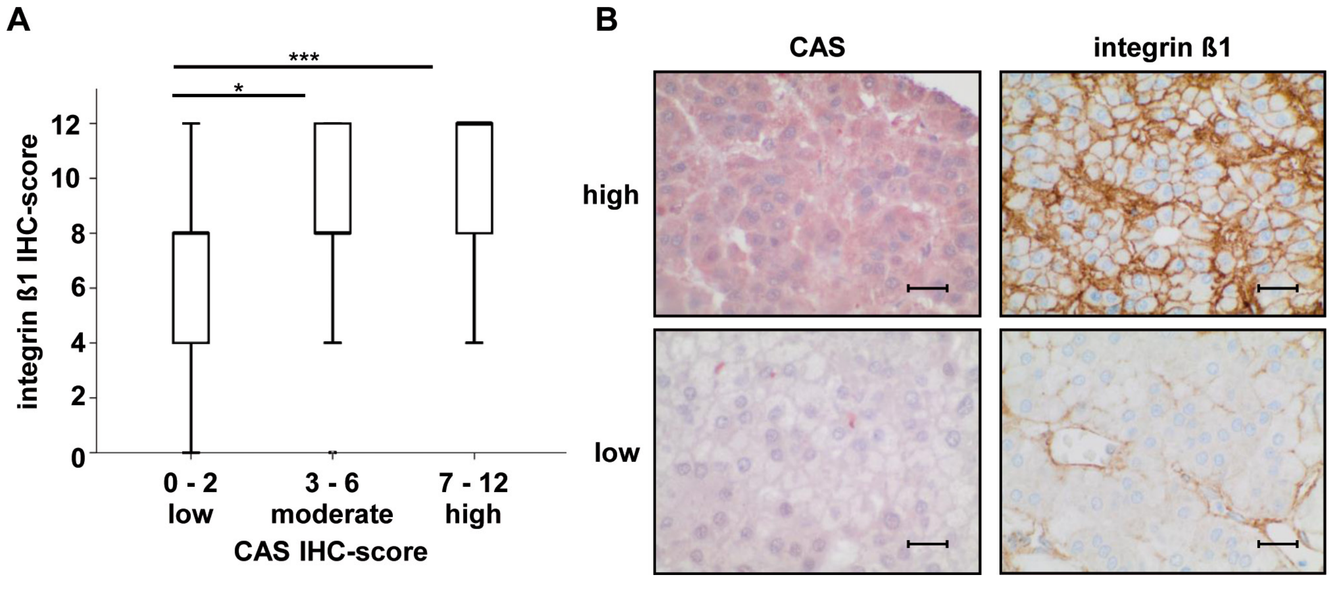

Supplementary Figure S4: CAS and integrin ß1 protein expression are correlated in HCC *in vivo*. (**A**) Box blot diagram shows immunohistochemical (IHC) score for integrin ß1 and CAS assessed by the product of IHC staining intensity and quantity in HCCs ($n = 91$). (**B**) Pictures illustrate IHC staining for CAS and corresponding IHC staining for integrin ß1 of the same HCC specimens. Shown are representative pictures for high (upper panel) and low (lower panel) expressed CAS and integrin ß1 protein, respectively. Scale bars = 20 µm.

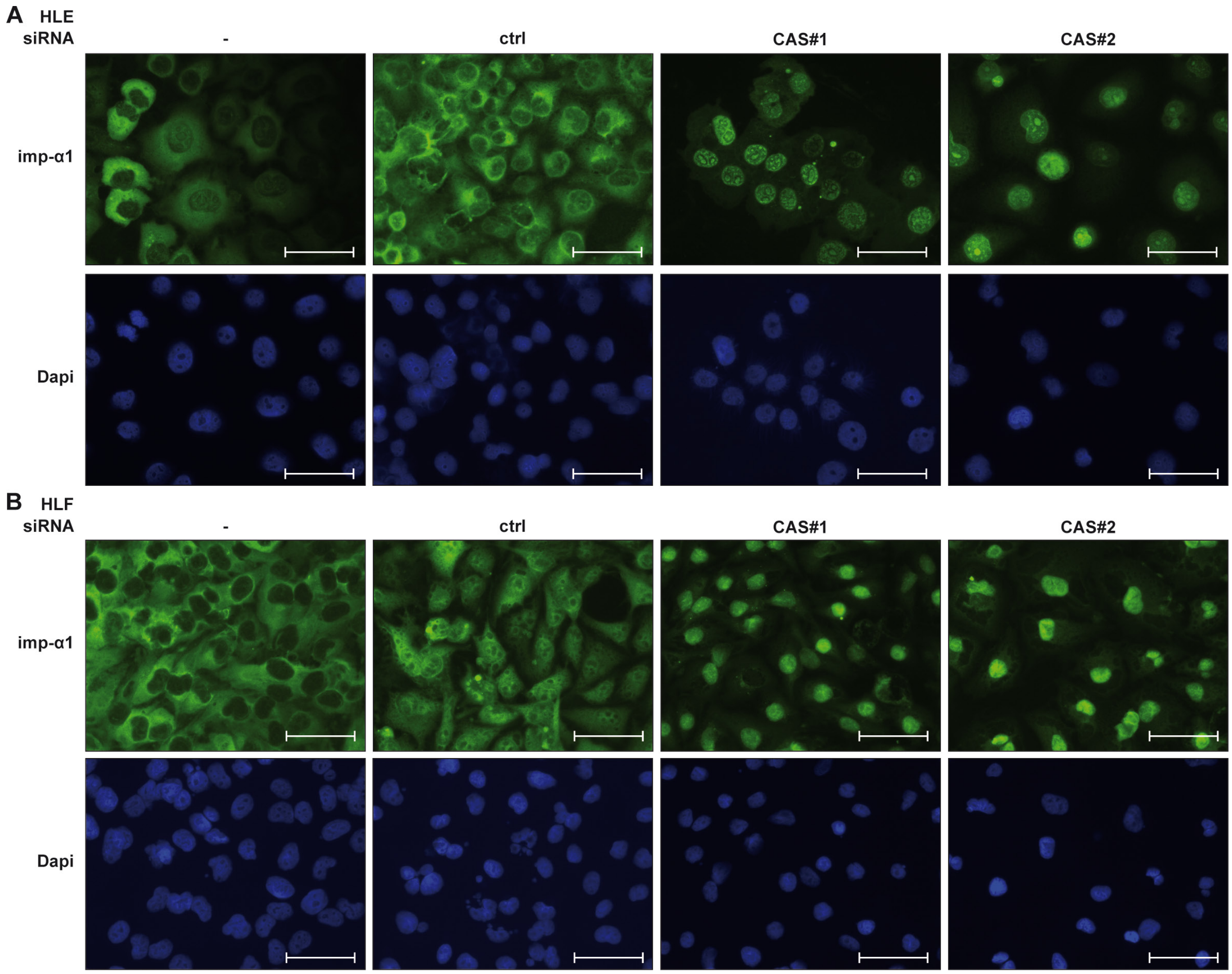

Supplementary Figure S5: CAS/imp-α1 transport cycle is active in HCC. HLE (**A**) or HLF (**B**) cells were treated either with a control (ctrl) or two different CAS specific siRNAs (CAS#1 and #2) for 48 h. Representative pictures show immunofluorescence staining of imp-α1 (upper panels) and DAPI (lower panel). Scale bars = 50 μm.

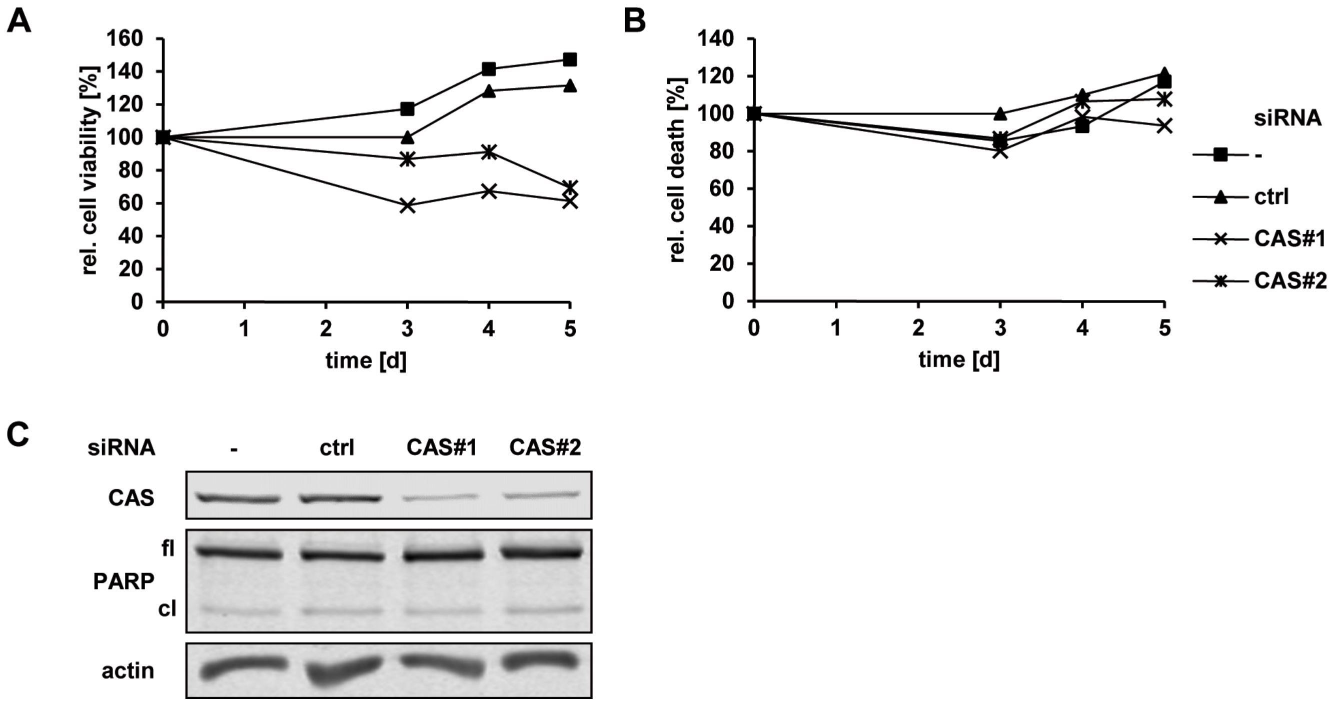

Supplementary Figure S6: Impact of CAS depletion on THLE-2 cells. THLE-2 cells were treated either with unspecific (ctrl) siRNA or two different CAS specific siRNAs (CAS#1, CAS#2). 3, 4 and 5 d after transfection of indicated siRNA relative cell viability was analyzed using CellTiterBlue assay (**A**) and relative cell death was assessed by Celltox assays (**B**). Data information: Data are normalized to the control siRNA condition and derived from a representative experiment. (**C**) Cell extracts were immunoblotted 72 h after transient transfection of the indicated siRNAs for CAS, full length (fl) and cleaved (cl) PARP (Poly (ADP-ribose) polymerase), and ß-actin.

Supplementary Table S1: Integrin ß1 protein is reduced after CAS silencing

Gene	Protein names	ID	logFC	AveExpr	*t*	*p value*	adj.*p*.val
XPO2	Exportin-2/Cellular apoptosis susceptibility protein	P55060	−2.1985	30.0777	18.7326	0.0000	0.0002
ITB1	Integrin beta-1	P05556	−3.1238	27.7467	18.3484	0.0000	0.0002
HMOX1	Heme oxygenase 1	P09601	1.3324	27.8160	−10.9492	0.0000	0.0049
ITAV	Integrin alpha-V	P06756	−1.0735	25.6552	10.7693	0.0000	0.0049
DDX21	DEAD box protein 21	Q9NR30	−0.9804	28.5947	9.9937	0.0000	0.0065
PLCB4	Phospholipase C-beta-4	Q15147	−1.2946	24.0757	10.2871	0.0000	0.0122
PLOD2	Procollagen-lysine,2-oxoglutarate 5-dioxygenase 2	O00469	−0.7651	26.2747	8.5488	0.0001	0.0130
TXND5	Thioredoxin domain-containing protein 5	Q8NBS9	−0.7611	28.7096	8.1797	0.0001	0.0144
RIN1	Ras interaction/interference protein 1	Q13671	1.0658	24.1875	−8.0556	0.0001	0.0144
SMTN	Smoothelin	P53814	1.2244	23.7498	−9.1478	0.0001	0.0144
RIR1	Ribonucleoside-diphosphate reductase subunit M1	P23921	−2.0054	26.2620	7.5252	0.0001	0.0176
RIR2	Ribonucleoside-diphosphate reductase subunit M2	P31350	1.0103	27.3705	−7.5105	0.0001	0.0176
EPN1	Epsin-1	Q9Y6I3	−1.3360	24.5112	8.4045	0.0001	0.0180
KITH	Thymidine kinase, cytosolic	P04183	1.2317	28.0824	−7.2206	0.0002	0.0194
VASP	Vasodilator-stimulated phosphoprotein	P50552	−0.7545	26.2834	7.1105	0.0002	0.0200
SDCB1	Syntenin-1	O00560	−1.4087	25.2429	6.8874	0.0002	0.0229
UB2V1	Ubiquitin-conjugating enzyme E2 variant 1	Q13404	−1.0540	26.6800	6.2405	0.0004	0.0394
DIM1	Probable dimethyladenosine transferase	Q9UNQ2	−0.6478	24.5636	6.1956	0.0004	0.0394
TACC3	Transforming acidic coiled-coil-containing protein 3	Q9Y6A5	0.6232	26.0344	−6.0407	0.0005	0.0421
COIA1	Collagen alpha-1(XVIII) chain	P39060	−1.1213	25.5146	6.6810	0.0005	0.0421
SMD2	snRNP core protein D2	P62316	−0.7139	28.1386	5.9756	0.0005	0.0421
SQSTM	Sequestosome-1	Q13501	0.8499	27.8123	−5.6426	0.0007	0.0544
PRC2C	BAT2 domain-containing protein 1	Q9Y520	−0.5996	25.1633	5.6302	0.0007	0.0544
RGAP1	Rac GTPase-activating protein 1	Q9H0H5	−0.5945	24.4421	5.5836	0.0008	0.0544
HMGB3	High mobility group protein B3	O15347	−0.6633	25.5558	5.5390	0.0008	0.0544
CLUS	Clusterin	P10909	0.6678	23.5104	−5.5258	0.0008	0.0544
LRC47	Leucine-rich repeat-containing protein 47	Q8N1G4	−0.5926	26.5287	5.4908	0.0009	0.0544
SPDLY	Protein Spindly (hSpindly)	Q96EA4	0.4920	26.1759	−5.4360	0.0009	0.0557
COPB	Coatomer subunit beta	P53618	−0.5899	28.0446	5.3705	0.0010	0.0577
AHNK	Desmoyokin	Q09666	−0.4833	33.4286	5.1023	0.0013	0.0699
NAGK	N-acetylglucosamine kinase	Q9UJ70	0.5024	26.1499	−5.0986	0.0013	0.0699
SPAG5	Sperm-associated antigen 5	Q96R06	−0.5666	23.8774	5.0843	0.0013	0.0699
UBS3B	Ubiquitin-associated and SH3 domain-containing protein B	Q8TF42	−0.4845	27.0154	5.0562	0.0014	0.0699
GLSK	Glutaminase kidney isoform, mitochondrial	O94925	−0.4906	28.2131	5.0185	0.0014	0.0699
PRDBP	Protein kinase C delta-binding protein (Cavin-3)	Q969G5	0.5562	25.6437	−5.0149	0.0015	0.0699

PAI1	Serpin E1	P05121	−0.6527	28.8215	4.9727	0.0015	0.0699
NHRF1	Na(+)/H(+) exchange regulatory cofactor NHE-RF1	O14745	0.6406	25.6295	−4.9641	0.0015	0.0699
SERPH	Serpin H1	P50454	0.5573	29.0719	−4.9591	0.0015	0.0699
PSD10	26S proteasome non-ATPase regulatory subunit 10	O75832	−1.1017	25.6587	4.9216	0.0016	0.0711
NT5D1	5'-nucleotidase domain-containing protein 1	Q5TFE4	0.7378	23.4476	−4.8714	0.0017	0.0734
PEPL	Periplakin	O60437	−0.4664	24.9020	4.8074	0.0018	0.0761
GALT2	Polypeptide N-acetylgalactosaminyltransferase 2	Q10471	−0.7386	24.8625	5.2088	0.0019	0.0761
GDIA	Rab GDP dissociation inhibitor alpha	P31150	−0.4776	27.3815	4.7334	0.0020	0.0803
PSMG2	Proteasome assembly chaperone 2	Q969U7	0.5559	24.5936	−5.1042	0.0021	0.0806
EDF1	Endothelial differentiation-related factor 1	O60869	0.7557	25.5827	−5.4739	0.0025	0.0933
ACOX1	Peroxisomal acyl-coenzyme A oxidase 1	Q15067	−0.5867	23.9060	4.5280	0.0026	0.0933
KI67	Antigen KI-67	P46013	0.4555	28.1474	−4.5261	0.0026	0.0933
HMOX2	Heme oxygenase 2	P30519	0.7789	25.6355	−4.5161	0.0026	0.0933
SRRM2	Serine/arginine repetitive matrix protein 2	Q9UQ35	−0.6411	27.1391	4.4845	0.0027	0.0939
RALB	Ras-related protein Ral-B	P11234	0.8833	24.2934	−4.8037	0.0028	0.0939
THIO	Thioredoxin	P10599	0.4396	28.7897	−4.4392	0.0029	0.0939
MDC1	Mediator of DNA damage checkpoint protein 1	Q14676	−0.9369	24.4615	4.7827	0.0029	0.0939
EXOS3	Exosome component 3	Q9NQT5	−0.6918	24.1024	4.7719	0.0029	0.0939
CND1	Condensin complex subunit 1	Q15021	−0.5391	25.8242	4.3349	0.0033	0.1036
CNOT1	CCR4-NOT transcription complex subunit 1	A5YKK6	−1.0893	24.9826	4.2725	0.0035	0.1099
RL27	60S ribosomal protein L27	P61353	−1.1255	28.1726	4.2319	0.0037	0.1136
DNM1L	Dynamin-1-like protein	O00429	0.4111	27.2669	−4.2043	0.0038	0.1156
PP6R3	Serine/threonine-protein phosphatase 6 regulatory subunit 3	Q5H9R7	−0.8597	24.3704	4.4483	0.0041	0.1213
GLYM	Serine hydroxymethyltransferase, mitochondrial	P34897	−0.4908	27.3193	4.1350	0.0042	0.1219
HS74L	Heat shock 70 kDa protein 4L	O95757	−0.5662	23.7324	4.0961	0.0044	0.1259
PP1R7	Protein phosphatase 1 regulatory subunit 7	Q15435	0.5038	24.6264	−4.0594	0.0046	0.1265
CCS	Copper chaperone for superoxide dismutase	O14618	0.6344	24.2118	−4.3343	0.0047	0.1265
CAZA2	F-actin-capping protein subunit alpha-2	P47755	0.4361	26.2665	−4.0483	0.0047	0.1265
TOP2A	DNA topoisomerase II alpha	P11388	−0.4391	27.0992	4.0350	0.0048	0.1265
TOP1	DNA topoisomerase I	P11387	−0.3663	26.9004	4.0186	0.0049	0.1265
GNL3	Guanine nucleotide-binding protein-like 3	Q9BVP2	−0.6123	25.8037	3.9953	0.0050	0.1265
GNA13	Guanine nucleotide-binding protein subunit alpha-13	Q14344	0.6399	24.2612	−4.2554	0.0051	0.1265
SPRE	Sepiapterin reductase	P35270	0.4701	26.4504	−3.9715	0.0052	0.1265
XPP1	Xaa-Pro aminopeptidase 1	Q9NQW7	0.5079	25.7910	−3.9332	0.0054	0.1265
NEDD8	Neddylin	Q15843	0.5632	24.6379	−4.1905	0.0055	0.1265

PSMD5	26S proteasome non-ATPase regulatory subunit 5	Q16401	−0.6028	24.5510	3.9253	0.0055	0.1265
MEP50	WD repeat-containing protein 77	Q9BQA1	0.4758	25.3509	−3.9060	0.0056	0.1265
CISD1	CDGSH iron-sulfur domain-containing protein 1	Q9NZ45	−0.9399	23.7550	4.5597	0.0057	0.1265
SRSF2	Serine/arginine-rich splicing factor 2	Q01130	−0.4781	26.3321	3.8913	0.0057	0.1265
TXNL1	Thioredoxin-like protein 1	O43396	0.4124	28.3072	−3.8661	0.0059	0.1265
PABP2	Polyadenylate-binding protein 2	Q86U42	−0.7324	25.2894	4.1164	0.0059	0.1265
KI20A	Kinesin-like protein KIF20A	O95235	−0.5346	25.3965	3.8631	0.0060	0.1265
SERA	D-3-phosphoglycerate dehydrogenase	O43175	0.3546	28.4372	−3.8581	0.0060	0.1265
PPIA	Peptidyl-prolyl cis-trans isomerase A	P62937	0.3943	31.3657	−3.8581	0.0060	0.1265
1433G	14-3-3 protein gamma	P61981	0.5908	27.5177	−3.8576	0.0060	0.1265
CK068	UPF0696 protein C11orf68	Q9H3H3	0.4173	25.1842	−3.8528	0.0060	0.1265
CATD	Cathepsin D	P07339	0.3668	27.6419	−3.8481	0.0061	0.1265
TXLNA	Alpha-taxilin	P40222	−0.4578	26.1599	3.8242	0.0063	0.1265
UFO	Tyrosine-protein kinase receptor UFO	P30530	−0.6821	24.7641	4.0628	0.0063	0.1265
VIME	Vimentin	P08670	0.3563	33.3655	−3.8161	0.0063	0.1265
PGM2	Phosphoglucomutase-2	Q96G03	0.6866	26.1771	−3.8153	0.0063	0.1265
CCD86	Coiled-coil domain-containing protein 86	Q9H6F5	−0.5266	24.5916	4.0469	0.0064	0.1266
APOL2	Apolipoprotein L2	Q9BQE5	0.5145	25.1718	−4.0340	0.0065	0.1266
PTN1	Tyrosine-protein phosphatase non-receptor type 1	P18031	0.5558	24.7134	−3.7815	0.0066	0.1266
PKN2	Protein-kinase C-related kinase 2	Q16513	−0.6849	24.3196	3.7733	0.0067	0.1266
ERLN1	Erlin-1	O75477	0.8769	25.7655	−3.7717	0.0067	0.1266
WDR82	WD repeat-containing protein 82	Q6UXN9	0.5044	24.8427	−3.7559	0.0069	0.1279
THTM	3-mercaptopyruvate sulfurtransferase	P25325	0.6842	28.0274	−3.9638	0.0071	0.1295
RTN4	Reticulon-4	Q9NQC3	−0.5101	28.5696	3.7284	0.0071	0.1295
CYR61	Protein CYR61	O00622	−0.6511	28.1620	3.7156	0.0072	0.1295
RL11	60S ribosomal protein L11	P62913	−0.4729	28.4686	3.7143	0.0073	0.1295
WDR12	Ribosome biogenesis protein WDR12	Q9GZL7	0.4236	26.3701	−3.6794	0.0076	0.1343
TP4A2	Protein tyrosine phosphatase type IVA 2	Q12974	−1.0790	24.7106	3.8812	0.0078	0.1365
NMES1	Normal mucosa of esophagus-specific gene 1 protein	Q9C002	0.7119	24.7265	−3.8691	0.0079	0.1367
NMI	N-myc-interactor	Q13287	0.3494	25.7931	−3.6437	0.0080	0.1367
PPP6	Serine/threonine-protein phosphatase 6 catalytic subunit	O00743	−0.4568	24.7217	3.6228	0.0082	0.1376
MMP1	Matrix metalloproteinase-1	P03956	0.4536	25.4111	−3.6107	0.0083	0.1376
CD166	Activated leukocyte cell adhesion molecule	Q13740	−0.3697	25.7731	3.6089	0.0084	0.1376
SMCE1	BRG1-associated factor 57	Q969G3	0.5418	24.4409	−3.8205	0.0084	0.1376
NEP1	Ribosomal RNA small subunit methyltransferase NEP1	Q92979	−0.4432	26.6072	3.6024	0.0084	0.1376
PR40A	Fas ligand-associated factor 1	O75400	−0.4208	23.4007	3.5885	0.0086	0.1389
AIM1	Absent in melanoma 1 protein	Q9Y4K1	−0.4607	23.5066	3.7800	0.0088	0.1399
CHMP6	Charged multivesicular body protein 6	Q96FZ7	0.3839	24.8347	−3.5676	0.0088	0.1399
PMVK	Phosphomevalonate kinase	Q15126	0.4582	24.4712	−3.5628	0.0089	0.1399
TMM43	Transmembrane protein 43	Q9BTV4	−0.6385	24.3604	4.0828	0.0090	0.1400

RL37A	60S ribosomal protein L37a	P61513	1.5188	26.6873	−3.5459	0.0091	0.1405
HCFC1	Host cell factor 1	P51610	−0.3449	25.8096	3.5122	0.0095	0.1456
TX1B3	Tax1-binding protein 3	O14907	0.6152	25.0499	−4.0174	0.0096	0.1456
NEXN	Nexilin	Q0ZGT2	−0.4842	24.4258	3.6910	0.0098	0.1472
CPSF7	Cleavage and polyadenylation specificity factor subunit 7	Q8N684	−0.7205	23.9396	3.9892	0.0099	0.1472
IBP7	Insulin-like growth factor-binding protein 7	Q16270	−0.7006	24.4597	3.4742	0.0100	0.1477
SPS2L	SPATS2-like protein	Q9NUQ6	0.6159	25.2745	−3.6559	0.0102	0.1477
AATM	Glutamate oxaloacetate transaminase 2	P00505	1.0666	25.9622	−3.6551	0.0102	0.1477
CAV1	Caveolin-1	Q03135	0.8902	26.3473	−3.4572	0.0103	0.1477
CALD1	Caldesmon	Q05682	−0.4215	25.6437	3.4467	0.0104	0.1477
PA1B2	Platelet-activating factor acetylhydrolase IB subunit beta	P68402	0.9485	25.5209	−3.4462	0.0104	0.1477
AAAT	Neutral amino acid transporter B(0)	Q15758	0.6118	26.0791	−3.4316	0.0106	0.1495
RDH11	Retinol dehydrogenase 11	Q8TC12	−0.4418	25.3536	3.4191	0.0108	0.1508
FKB1A	Peptidyl-prolyl cis-trans isomerase FKBP1A	P62942	0.4535	29.0829	−3.4126	0.0109	0.1509
PDXK	Pyridoxal kinase	O00764	0.9329	23.9102	−3.5641	0.0114	0.1567
G6PI	Glucose-6-phosphate isomerase	P06744	0.3146	28.2650	−3.3687	0.0116	0.1567
NU153	Nucleoporin Nup153	P49790	−0.4061	24.0969	3.3656	0.0116	0.1567
SPTB2	Spectrin beta chain, non-erythrocytic 1	Q01082	−0.3247	28.3518	3.3626	0.0117	0.1567
RBBP7	Retinoblastoma-binding protein 7	Q16576	0.6905	26.2029	−3.3353	0.0121	0.1615
SRRM1	Serine/arginine repetitive matrix protein 1	Q8IYB3	−0.3789	25.1408	3.3093	0.0126	0.1661
RL38	60S ribosomal protein L38	P63173	−0.4300	26.4331	3.2869	0.0130	0.1694
SFPQ	Splicing factor, proline- and glutamine-rich	P23246	−0.3177	29.5622	3.2813	0.0131	0.1694
GNAI2	Guanine nucleotide-binding protein G(i) subunit alpha-2	P04899	0.3579	26.9814	−3.2787	0.0131	0.1694
PRAF3	ADP-ribosylation factor-like protein 6-interacting protein 5	O75915	0.5194	24.0022	−3.6849	0.0135	0.1733
WDR44	WD repeat-containing protein 44	Q5JSH3	−0.3446	24.8456	3.2305	0.0141	0.1761
MAP1S	Microtubule-associated protein 1S	Q66K74	0.7711	23.6662	−3.3959	0.0141	0.1761
CND3	Condensin complex subunit 3	Q9BPX3	−0.3907	24.8273	3.3933	0.0141	0.1761
FKBP5	Peptidyl-prolyl cis-trans isomerase FKBP5	Q13451	0.5371	24.5687	−3.6365	0.0143	0.1761
ROA0	Heterogeneous nuclear ribonucleoprotein A0	Q13151	0.7744	25.7337	−3.2198	0.0143	0.1761
TOM34	Translocase of outer membrane 34 kDa subunit	Q15785	0.3837	25.7636	−3.2121	0.0144	0.1765
AL7A1	Alpha-aminoadipic semialdehyde dehydrogenase	P49419	0.3651	27.0838	−3.2076	0.0145	0.1765
CDK1	Cyclin-dependent kinase 1	P06493	−0.5766	27.4934	3.1986	0.0147	0.1775
GSHR	Glutathione reductase, mitochondrial	P00390	0.3424	27.4120	−3.1853	0.0150	0.1796
SMD1	Small nuclear ribonucleoprotein Sm D1	P62314	−0.7132	26.7467	3.3308	0.0153	0.1817
ADRM1	Adhesion-regulating molecule 1	Q16186	0.4262	25.1328	−3.1310	0.0162	0.1913
CLIP1	Cytoplasmic linker protein 1	P30622	0.3419	24.6631	−3.1206	0.0164	0.1919

DHX15	Pre-mRNA-splicing factor ATP-dependent RNA helicase DHX15	O43143	−0.3820	27.4279	3.1189	0.0164	0.1919
CDC42	Cell division control protein 42 homolog	P60953	0.3159	26.4143	−3.1097	0.0167	0.1919
ANLN	Actin-binding protein anillin	Q9NQW6	1.3057	26.1082	−3.1093	0.0167	0.1919
DEK	Protein DEK	P35659	−0.3679	26.4281	3.0816	0.0173	0.1967
SMRC2	SWI/SNF complex subunit SMARCC2	Q8TAQ2	−0.3144	25.3124	3.0791	0.0174	0.1967
IPO9	Importin-9	Q96P70	−0.3479	26.0211	3.0779	0.0174	0.1967
CLCB	Clathrin light chain B	P09497	0.3618	26.3165	−3.2168	0.0176	0.1975
COPD	Archain	P48444	−0.2886	28.0656	3.0659	0.0177	0.1975
IDI1	Isopentenyl-diphosphate Delta-isomerase 1	Q13907	−0.5315	25.5491	3.0531	0.0181	0.1998
RO60	60 kDa Ro protein	P10155	−0.5663	23.5094	3.1803	0.0185	0.2006
ARP2	Actin-related protein 2	P61160	−0.4782	27.0212	3.0352	0.0185	0.2006
SDHB	Iron-sulfur subunit of complex II	P21912	−0.5473	23.7162	3.1676	0.0188	0.2006
ADPPT	L-aminoadipate-semialdehyde dehydrogenase-phosphopantetheinyl transferase	Q9NRN7	0.5225	24.4907	−3.3802	0.0189	0.2006
LONM	Lon protease homolog, mitochondrial	P36776	0.3260	24.3384	−3.0218	0.0189	0.2006
MTAP	S-methyl-5'-thioadenosine phosphorylase	Q13126	0.5257	26.1599	−3.0213	0.0189	0.2006
SGT1	Protein SGT1 homolog	Q9Y2Z0	−0.3287	26.6522	3.0196	0.0189	0.2006
DPM3	DPM synthase subunit 3	Q9P2X0	0.4667	24.7178	−3.1406	0.0194	0.2011
CDV3	Protein CDV3 homolog	Q9UKY7	0.4637	28.3426	−2.9968	0.0196	0.2011
AP1B1	AP-1 complex subunit beta-1	Q10567	0.4843	25.0174	−2.9959	0.0196	0.2011
ARP10	Actin-related protein 10	Q9NZ32	−0.5631	23.9134	3.1332	0.0196	0.2011
SDPR	Serum deprivation-response protein	O95810	1.0338	23.8068	−3.3391	0.0197	0.2011
HNRH2	Heterogeneous nuclear ribonucleoprotein H2	P55795	−0.5701	24.1983	2.9904	0.0198	0.2011
RCL1	RNA 3'-terminal phosphate cyclase-like protein	Q9Y2P8	−0.5526	24.5338	2.9882	0.0198	0.2011
SBDS	Ribosome maturation protein SBDS	Q9Y3A5	−0.3663	25.7416	2.9603	0.0206	0.2025
PUR2	Trifunctional purine biosynthetic protein adenosine-3	P22102	−0.3683	28.3275	2.9595	0.0206	0.2025
BOLA2	BolA-like protein 2	Q9H3K6	0.4247	26.4484	−2.9472	0.0210	0.2025
DOC10	Dedicator of cytokinesis protein 10	Q96BY6	−0.4971	23.3956	3.0794	0.0210	0.2025
CALR	Calreticulin	P27797	0.3828	29.7272	−2.9431	0.0211	0.2025
UB2G1	Ubiquitin-conjugating enzyme E2 G1	P62253	0.4099	23.1829	−2.9421	0.0212	0.2025
KTHY	Thymidylate kinase	P23919	−0.4587	26.0374	2.9414	0.0212	0.2025
NCBP1	Nuclear cap-binding protein subunit 1	Q09161	−0.3370	25.1884	2.9394	0.0213	0.2025
AL1A1	Aldehyde dehydrogenase family 1 member A1	P00352	−0.3165	32.2719	2.9347	0.0214	0.2025
PHF6	PHD finger protein 6	Q8IWS0	−0.6140	25.1721	2.9316	0.0215	0.2025
RM12	39S ribosomal protein L12, mitochondrial	P52815	−0.3806	27.5148	2.9304	0.0215	0.2025
HMGB1	High mobility group protein B1	P09429	0.4478	28.5718	−2.9277	0.0216	0.2025
TPPC3	Trafficking protein particle complex subunit 3	O43617	0.5335	24.6922	−3.0590	0.0216	0.2025
CD59	CD59 glycoprotein	P13987	0.3807	28.6220	−2.9212	0.0218	0.2025

NUDT5	Nucleoside diphosphate-linked moiety X motif 5	Q9UKK9	0.3813	25.2922	−2.9160	0.0220	0.2025
PEBP1	Phosphatidylethanolamine-binding protein 1	P30086	0.3391	28.1248	−2.9135	0.0221	0.2025
CSDE1	Cold shock domain-containing protein E1	O75534	−0.3423	27.6575	2.9078	0.0222	0.2025
OAT	Ornithine aminotransferase, mitochondrial	P04181	0.4476	26.4667	−2.9031	0.0224	0.2025
TR112	tRNA methyltransferase 112 homolog	Q9UI30	−0.3113	26.2741	2.9017	0.0224	0.2025
RADI	Radixin	P35241	0.3217	28.0429	−2.8979	0.0226	0.2025
SLK	Serine/threonine-protein kinase 2	Q9H2G2	0.3650	25.7792	−2.8974	0.0226	0.2025
KCY	UMP/CMP kinase	P30085	−0.3817	25.2715	2.8966	0.0226	0.2025
CC137	Coiled-coil domain-containing protein 137	Q6PK04	−0.5175	24.0902	3.0214	0.0227	0.2025
ANX11	Annexin A11	P50995	0.7468	24.8382	−3.2115	0.0228	0.2025
UBE2O	E2 ubiquitin-conjugating enzyme	Q9C0C9	0.3487	25.4194	−2.8798	0.0232	0.2047
BROX	BRO1 domain-containing protein BROX	Q5VW32	−0.5546	23.4291	2.9911	0.0236	0.2077
MAT2B	Methionine adenosyltransferase II beta	Q9NZL9	−0.3387	26.7099	2.8602	0.0238	0.2084
TM263	Transmembrane protein 263	Q8WUH6	0.6670	24.3620	−2.9804	0.0240	0.2085
FBRL	rRNA 2'-O-methyltransferase fibrillarin	P22087	−0.3956	26.0337	2.8393	0.0246	0.2118
LMNB2	Lamin-B2	Q03252	0.3045	26.9358	−2.8388	0.0246	0.2118
OPTN	Optineurin	Q96CV9	−0.4812	23.1698	3.1362	0.0248	0.2129
PDIA6	Protein disulfide-isomerase A6	Q15084	0.3267	29.4790	−2.8219	0.0252	0.2149
SMU1	WD40 repeat-containing protein SMU1	Q2TAY7	−0.3750	23.6422	2.9340	0.0255	0.2162
HSDL2	Hydroxysteroid dehydrogenase-like protein 2	Q6YN16	0.3789	25.3163	−2.8087	0.0257	0.2169
SPTN1	Spectrin alpha chain, non-erythrocytic 1	Q13813	−0.2702	28.5286	2.7909	0.0263	0.2207
ATD3B	ATPase family AAA domain-containing protein 3B	Q5T9A4	0.3636	25.0565	−2.7898	0.0264	0.2207
SH3K1	SH3 domain-containing kinase-binding protein 1	Q96B97	−0.2759	26.7945	2.7671	0.0273	0.2252
2AAB	PP2A subunit A isoform R1-beta	P30154	−0.4831	25.5909	2.8808	0.0273	0.2252
VPP1	V-ATPase 116 kDa isoform a1	Q93050	−0.6068	24.4273	3.0509	0.0274	0.2252
PFD2	Prefoldin subunit 2	Q9UHV9	0.4597	27.0032	−2.7568	0.0277	0.2252
COX2	Cytochrome c oxidase polypeptide II	P00403	0.3388	25.7742	−2.7565	0.0277	0.2252
WFS1	Wolframin	O76024	0.3745	24.0470	−2.7528	0.0278	0.2252
FDFT	Farnesyl-diphosphate farnesyltransferase	P37268	−0.6016	22.9594	3.0342	0.0279	0.2252
CHMP5	Chromatin-modifying protein 5	Q9NZZ3	−0.4238	23.0322	3.0318	0.0280	0.2252
TRM1	tRNA (guanine(26)-N(2))-dimethyltransferase	Q9NXH9	0.4052	22.7748	−2.8594	0.0281	0.2252
SRC8	Src substrate cortactin	Q14247	0.3009	28.4607	−2.7434	0.0282	0.2252
TPR	Nucleoprotein TPR	P12270	−0.2645	26.4330	2.7313	0.0287	0.2262
P66B	GATA zinc finger domain-containing protein 2B	Q8WXI9	−0.5661	23.3312	2.8415	0.0288	0.2262
XPO7	Exportin-7	Q9UIA9	−0.2543	25.5776	2.7263	0.0289	0.2262
PPAC	Adipocyte acid phosphatase	P24666	−0.4898	25.9835	2.7255	0.0290	0.2262
NPM	Nucleophosmin	P06748	0.2875	32.0296	−2.7246	0.0290	0.2262

RU2A	U2 small nuclear ribonucleoprotein A'	P09661	−0.3559	26.2070	2.7158	0.0294	0.2265
RL24	60S ribosomal protein L24	P83731	−0.3233	28.9231	2.7127	0.0295	0.2265
CSN5	COP9 signalosome complex subunit 5	Q92905	0.3349	24.5939	−2.7102	0.0296	0.2265
IMPA1	Inositol monophosphatase 1	P29218	−0.3809	24.5865	2.8182	0.0297	0.2265
AT1B3	Sodium/potassium-transporting ATPase subunit beta-3	P54709	−0.4705	26.2351	2.7008	0.0300	0.2265
RAB32	Ras-related protein Rab-32	Q13637	0.4814	23.9146	−2.8075	0.0301	0.2265
DDX3X	ATP-dependent RNA helicase DDX3X	O00571	−0.3361	28.4677	2.6978	0.0302	0.2265
DDX17	Probable ATP-dependent RNA helicase DDX17	Q92841	−0.2675	27.9516	2.6973	0.0302	0.2265
COPZ1	Coatomer subunit zeta-1	P61923	0.2777	25.9594	−2.6916	0.0304	0.2265
GTSF1	Gametocyte-specific factor 1	Q8WW33	−1.0963	24.5916	2.7956	0.0306	0.2265
PTH2	Bcl-2 inhibitor of transcription 1	Q9Y3E5	0.5903	25.3384	−2.6864	0.0307	0.2265
JIP4	JNK-interacting protein 4	O60271	0.3145	26.4621	−2.6846	0.0308	0.2265
SNX5	Sorting nexin-5	Q9Y5X3	0.3567	25.2462	−2.6843	0.0308	0.2265
NH2L1	U4/U6.U5 small nuclear ribonucleoprotein SNU13	P55769	−0.5276	27.5890	2.6811	0.0309	0.2266
ATLA3	Atlastin-3	Q6DD88	−0.2843	27.4898	2.6616	0.0318	0.2294
YBOX1	Y-box transcription factor	P67809	0.2921	29.1560	−2.6586	0.0320	0.2294
SCFD1	Sec1 family domain-containing protein 1	Q8WVM8	−0.3478	25.6950	2.6567	0.0320	0.2294
AATF	Apoptosis-antagonizing transcription factor	Q9NY61	−0.4405	24.7099	2.6566	0.0320	0.2294
RHG01	Rho GTPase-activating protein 1	Q07960	0.4374	26.5678	−2.6489	0.0324	0.2294
GT251	Collagen beta(1-O) galactosyltransferase 1	Q8NBJ5	0.2956	25.6001	−2.6488	0.0324	0.2294
HINT1	Protein kinase C inhibitor 1	P49773	−0.3862	26.5917	2.7516	0.0324	0.2294
ISG15	Interferon-induced 15 kDa protein	P05161	0.9788	25.6745	−2.7490	0.0326	0.2294
PSDE	26S proteasome non-ATPase regulatory subunit 14	O00487	0.3227	27.0822	−2.6423	0.0327	0.2294
SYFB	Phenylalanine-tRNA ligase beta subunit	Q9NSD9	−0.2617	27.5979	2.6354	0.0331	0.2294
RCC2	Protein RCC2	Q9P258	−0.3736	27.2528	2.6354	0.0331	0.2294
SYAP1	Synapse-associated protein 1	Q96A49	0.5114	24.5141	−2.6268	0.0335	0.2294
OFUT1	GDP-fucose protein O-fucosyltransferase 1	Q9H488	0.5194	25.3866	−2.6264	0.0335	0.2294
RBBP4	Retinoblastoma-binding protein 4	Q09028	0.4102	26.9084	−2.6242	0.0336	0.2294
CYC	Cytochrome c	P99999	0.3775	27.9251	−2.6234	0.0336	0.2294
RRP5	NF-kappa-B-binding protein	Q14690	−0.3006	25.6299	2.6220	0.0337	0.2294
TBL3	Transducin beta-like protein 3	Q12788	0.2800	25.9225	−2.6205	0.0338	0.2294
SRRT	Serrate RNA effector molecule homolog	Q9BXP5	−0.3026	26.6853	2.6186	0.0339	0.2294
CSTF2	Cleavage stimulation factor subunit 2	P33240	0.3172	25.7185	−2.6183	0.0339	0.2294
ODO1	2-oxoglutarate dehydrogenase, mitochondrial	Q02218	0.2994	24.5119	−2.6168	0.0340	0.2294
UT14A	U3 small nucleolar RNA-associated protein 14 homolog A	Q9BVJ6	−0.6461	23.4766	2.7109	0.0343	0.2305
NXN	Nucleoredoxin	Q6DKJ4	0.5703	24.3272	−2.6080	0.0344	0.2306
IF4E	Eukaryotic translation initiation factor 4E	P06730	0.2811	25.5828	−2.5958	0.0350	0.2336

LAP2A	Thymopentin	P42166	−0.3789	27.8383	2.5931	0.0352	0.2336
ERGI3	Endoplasmic reticulum-Golgi intermediate compartment protein 3	Q9Y282	0.8877	24.3115	−2.8342	0.0354	0.2336
PRP31	(U4/U6 snRNP 61 kDa protein	Q8WWY3	−0.3330	25.0713	2.5887	0.0354	0.2336
PPCE	Prolyl endopeptidase	P48147	0.3500	24.1236	−2.5798	0.0359	0.2354
IQGA1	Ras GTPase-activating-like protein IQGAP1	P46940	0.2715	29.1752	−2.5780	0.0360	0.2354
IDH3B	Isocitrate dehydrogenase [NAD] subunit beta, mitochondrial	O43837	−0.9358	25.1093	2.5715	0.0363	0.2364
ACACA	Acetyl-CoA carboxylase 1	Q13085	−0.4167	24.8415	2.5702	0.0364	0.2364
NSF1C	NSFL1 cofactor p47	Q9UNZ2	0.3660	28.7333	−2.5655	0.0366	0.2366
RL7A	60S ribosomal protein L7a	P62424	−0.2430	30.6398	2.5637	0.0367	0.2366
UBCP1	Ubiquitin-like domain-containing CTD phosphatase 1	Q8WVY7	0.2931	24.2548	−2.5605	0.0369	0.2366
RL32	60S ribosomal protein L32	P62910	−0.5199	28.1705	2.5592	0.0370	0.2366
ANM3	Protein arginine N-methyltransferase 3	O60678	−0.4142	25.4827	2.5547	0.0372	0.2369
MARK2	MAP/microtubule affinity-regulating kinase 2	Q7KZI7	−0.4661	25.1056	2.5498	0.0375	0.2369
ANXA6	Annexin A6	P08133	0.4199	28.4675	−2.5469	0.0377	0.2369
NUMA1	Nuclear mitotic apparatus protein 1	Q14980	−0.2994	27.0813	2.5461	0.0377	0.2369
TKT	Transketolase	P29401	0.3263	30.5112	−2.5460	0.0377	0.2369
DSRAD	Double-stranded RNA-specific adenosine deaminase	P55265	−0.4520	24.7739	2.5403	0.0380	0.2380
RFA3	Replication factor A protein 3	P35244	0.4361	25.0554	−2.5373	0.0382	0.2380
SNX6	Sorting nexin-6	Q9UNH7	−0.3564	25.8003	2.5337	0.0384	0.2380
TCOF	Treacle protein	Q13428	0.4771	26.4158	−2.5329	0.0384	0.2380
STX7	Syntaxin-7	O15400	0.2634	24.9537	−2.5255	0.0389	0.2382
VDAC1	Voltage-dependent anion-selective channel protein 1	P21796	0.2865	30.2213	−2.5249	0.0389	0.2382
ABCE1	ATP-binding cassette sub-family E member 1	P61221	0.2497	28.6760	−2.5241	0.0389	0.2382
DUS3	Dual specificity protein phosphatase 3	P51452	0.2916	27.2860	−2.5226	0.0390	0.2382
RECQ1	ATP-dependent DNA helicase Q1	P46063	0.3183	26.6168	−2.5198	0.0392	0.2383
PSPC1	Paraspeckle component 1	Q8WXF1	0.2916	25.9168	−2.5105	0.0397	0.2408
EGFR	Epidermal growth factor receptor	P00533	−0.4113	27.0828	2.5074	0.0399	0.2411
GLCM	Glucosylceramidase	P04062	0.4410	23.8829	−2.5946	0.0401	0.2411
KIME	Mevalonate kinase	Q03426	0.3631	24.8136	−2.5929	0.0402	0.2411
SPT6H	Transcription elongation factor SPT6	Q7KZ85	−0.2931	24.4474	2.4984	0.0404	0.2417
FGF2	Fibroblast growth factor 2	P09038	−0.9682	24.5644	2.4866	0.0412	0.2451
RL9	60S ribosomal protein L9	P32969	0.8286	29.5700	−2.4784	0.0417	0.2473
GNPI1	Glucosamine-6-phosphate isomerase 1	P46926	0.3227	27.4544	−2.4745	0.0419	0.2478
SCAM1	Secretory carrier-associated membrane protein 1	O15126	0.2682	27.4779	−2.4673	0.0424	0.2496
MCTS1	Malignant T-cell-amplified sequence 1	Q9ULC4	−0.5135	26.1355	2.4620	0.0427	0.2507
SYQ	Glutaminyl-tRNA synthetase	P47897	0.2676	27.5754	−2.4578	0.0430	0.2509
XPO5	Exportin-5	Q9HAV4	−0.3487	25.7286	2.4568	0.0430	0.2509
CD44	CD44 antigen	P16070	0.2762	29.7129	−2.4527	0.0433	0.2514

ENAH	Protein enabled homolog	Q8N8S7	0.3931	25.1832	−2.4510	0.0434	0.2514
RS3A	40S ribosomal protein S3a	P61247	−0.3056	30.4184	2.4412	0.0440	0.2542
BLVRB	Biliverdin reductase B	P30043	0.3276	27.8669	−2.4310	0.0447	0.2572
IF5	Eukaryotic translation initiation factor 5	P55010	−0.4044	25.7387	2.4257	0.0450	0.2584
SNUT2	U4/U6.U5 tri-snRNP-associated protein 2	Q53GS9	0.4022	25.1580	−2.4204	0.0454	0.2587
AAK1	Adaptor-associated kinase 1	Q2M2I8	−0.3825	22.8420	2.4203	0.0454	0.2587
DDX6	Probable ATP-dependent RNA helicase DDX6	P26196	0.2228	27.8688	−2.4169	0.0456	0.2591
PRP8	Pre-mRNA-processing-splicing factor 8	Q6P2Q9	−0.2495	27.6261	2.4127	0.0459	0.2596
IMA4	Importin subunit alpha-4	O00505	−0.2214	25.7239	2.4112	0.0460	0.2596
SPEE	Spermidine synthase	P19623	0.2750	27.6544	−2.4068	0.0463	0.2597
PCNP	PEST-containing nuclear protein	Q8WW12	−0.2217	26.4345	2.4033	0.0466	0.2597
CALU	Calumenin	O43852	0.2798	26.7911	−2.4031	0.0466	0.2597
FKB10	Peptidyl-prolyl cis-trans isomerase FKBP10	Q96AY3	−0.2166	25.9075	2.4023	0.0466	0.2597
COX5A	Cytochrome c oxidase subunit 5A, mitochondrial	P20674	−0.2601	26.3260	2.3980	0.0469	0.2605
OGA	Protein O-GlcNAcase	O60502	0.7530	25.1138	−2.3956	0.0471	0.2606
CEGT	Ceramide glucosyltransferase	Q16739	−0.4347	24.2483	2.3883	0.0476	0.2621
NUBP2	Nucleotide-binding protein 2	Q9Y5Y2	0.3919	24.0403	−2.4681	0.0477	0.2621
CBR1	NADPH-dependent carbonyl reductase 1	P16152	0.2482	27.6512	−2.3793	0.0482	0.2644
RFC5	Replication factor C subunit 5	P40937	−0.4547	25.5434	2.3720	0.0488	0.2664
G3BP1	GAP SH3 domain-binding protein 1	Q13283	−0.2548	28.1472	2.3614	0.0495	0.2698
EZRI	Ezrin	P15311	0.2737	27.8695	−2.3534	0.0501	0.2721
TWF2	Twinfilin-2	Q6IBS0	0.4557	26.8293	−2.3451	0.0508	0.2745
TBCB	Tubulin-folding cofactor B	Q99426	−0.2333	26.8525	2.3433	0.0509	0.2745
CLCA	Clathrin light chain A	P09496	0.3867	26.8930	−2.3384	0.0513	0.2756
FUBP2	Far upstream element-binding protein 2	Q92945	0.2799	28.0304	−2.3324	0.0517	0.2772
NENF	Neudesin	Q9UMX5	−0.4146	24.6252	2.4046	0.0520	0.2780
CAN2	Calpain-2 catalytic subunit	P17655	−0.3082	28.3678	2.3123	0.0533	0.2830
DDB1	DNA damage-binding protein 1	Q16531	−0.2568	28.0624	2.3114	0.0534	0.2830
H15	Histone H1a	P16401	−0.4099	29.0471	2.3101	0.0535	0.2830
LTOR1	Ragulator complex protein LAMTOR1	Q6IAA8	0.2686	24.9888	−2.3061	0.0538	0.2839
NOTC2	Neurogenic locus notch homolog protein 2	Q04721	0.5017	25.0475	−2.2986	0.0544	0.2862
TF65	Nuclear factor NF-kappa-B p65 subunit	Q04206	−0.4006	24.5661	2.2926	0.0549	0.2874
6PGD	6-phosphogluconate dehydrogenase, decarboxylating	P52209	0.2512	28.4987	−2.2886	0.0552	0.2874
COPA	Coatomer subunit alpha	P53621	−0.3026	27.8823	2.2885	0.0552	0.2874
NACAM	Nascent polypeptide-associated complex subunit alpha, muscle-specific form	E9PAV3	−0.2326	28.5983	2.2875	0.0553	0.2874
VAMP3	Vesicle-associated membrane protein 3	Q15836	0.5273	26.3127	−2.2845	0.0555	0.2876
IPO8	Importin-8	O15397	0.8619	24.0075	−2.2830	0.0557	0.2876
HAT1	Histone acetyltransferase type B catalytic subunit	O14929	0.2591	26.0899	−2.2769	0.0562	0.2893
TPX2	Targeting protein for Xklp2	Q9ULW0	0.3502	25.4839	−2.2736	0.0565	0.2899

1433B	Protein kinase C inhibitor protein 1	P31946	0.3187	29.9612	−2.2702	0.0567	0.2905
CAPG	Actin regulatory protein CAP-G	P40121	0.2494	26.4268	−2.2654	0.0571	0.2917
GNA11	Guanine nucleotide-binding protein subunit alpha-11	P29992	0.9877	24.9719	−2.3280	0.0578	0.2937
CD11B	Cyclin-dependent kinase 11B	P21127	0.3680	24.4411	−2.2569	0.0579	0.2937
RL26L	60S ribosomal protein L26-like 1	Q9UNX3	0.4174	28.1456	−2.2539	0.0581	0.2941
CHM4B	Chromatin-modifying protein 4b	Q9H444	−0.2726	27.7759	2.2482	0.0586	0.2957
CCDC6	Coiled-coil domain-containing protein 6	Q16204	0.5656	24.1036	−2.3162	0.0588	0.2957
RS17	40S ribosomal protein S17	P08708	0.3195	28.3930	−2.2386	0.0595	0.2976
ECHD1	Enoyl-CoA hydratase domain-containing protein 1	Q9NTX5	−0.2686	24.7809	2.2370	0.0596	0.2976
HACD3	3-hydroxyacyl-CoA dehydratase 3	Q9P035	0.5174	24.2697	−2.2360	0.0597	0.2976
CTNA1	Alpha E-catenin	P35221	−0.2212	27.6239	2.2300	0.0602	0.2982
STX12	Syntaxin-12	Q86Y82	1.2228	23.5178	−2.3991	0.0603	0.2982
MCM5	DNA replication licensing factor MCM5	P33992	0.2017	27.3447	−2.2284	0.0604	0.2982
HPBP1	Hsp70-binding protein 1	Q9NZL4	0.5254	24.2477	−2.3966	0.0605	0.2982
TPD52	Tumor protein D52	P55327	−0.2617	25.8301	2.2202	0.0611	0.2984
SNX1	Sorting nexin-1	Q13596	−0.3293	25.6027	2.2188	0.0612	0.2984
TRI47	Tripartite motif-containing protein 47	Q96LD4	0.7195	24.6997	−2.3858	0.0613	0.2984
PDIA1	Prolyl 4-hydroxylase subunit beta	P07237	0.2169	30.5083	−2.2172	0.0614	0.2984
TMX1	Thioredoxin-related transmembrane protein 1	Q9H3N1	−0.2385	26.6942	2.2168	0.0614	0.2984
API5	Apoptosis inhibitor 5	Q9BZZ5	0.2972	25.8602	−2.2151	0.0616	0.2984
BASP1	Brain acid soluble protein 1	P80723	0.3428	28.6838	−2.2115	0.0619	0.2991
CAZA1	F-actin-capping protein subunit alpha-1	P52907	−0.2591	28.1457	2.2093	0.0621	0.2992
GRN	Granulins	P28799	0.4399	24.8217	−2.2061	0.0624	0.2992
PUR1	Amidophosphoribosyltransferase (ATase)	Q06203	−0.3638	24.9631	2.2056	0.0625	0.2992
RL36	60S ribosomal protein L36	Q9Y3U8	0.2564	27.2266	−2.2641	0.0632	0.3016
SEC63	Translocation protein SEC63 homolog	Q9UGP8	−0.3801	25.4461	2.1967	0.0633	0.3016
CISY	Citrate synthase, mitochondrial	O75390	−0.2159	28.2122	2.1874	0.0642	0.3032
ROCK2	Rho-associated protein kinase 2	O75116	−0.2950	24.5200	2.1870	0.0642	0.3032
DCTP1	dCTP pyrophosphatase 1	Q9H773	0.6066	25.2597	−2.1846	0.0645	0.3032
COX17	Cytochrome c oxidase copper chaperone	Q14061	−1.0505	24.3163	2.2493	0.0645	0.3032
ERAP1	Endoplasmic reticulum aminopeptidase 1	Q9NZ08	−0.3560	25.2657	2.1826	0.0646	0.3032
EF1D	Elongation factor 1-delta	P29692	0.2626	29.8996	−2.1819	0.0647	0.3032
ARF6	ADP-ribosylation factor 6	P62330	−0.7858	26.3132	2.1749	0.0654	0.3055
PNPT1	Polyribonucleotide nucleotidyltransferase 1, mitochondrial	Q8TCS8	−0.2996	24.7825	2.1687	0.0660	0.3076
AHNK2	Protein AHNAK2	Q8IVF2	−0.2127	29.6196	2.1603	0.0668	0.3106
CIP4	Thyroid receptor-interacting protein 10	Q15642	0.4430	25.8222	−2.1552	0.0673	0.3116
SYFA	Phenylalanine-tRNA ligase alpha subunit	Q9Y285	−0.2167	25.2509	2.1545	0.0674	0.3116
DIC	Solute carrier family 25 member 10	Q9UBX3	0.3951	26.2469	−2.1517	0.0677	0.3120
SVIL	Supervillin	O95425	−0.2414	26.0590	2.1487	0.0680	0.3126
HS71A	Heat shock 70 kDa protein 1A	P08107	−0.2801	28.3616	2.1418	0.0687	0.3139

HS71B	Heat shock 70 kDa protein 1B	P08107	−0.2801	28.3616	2.1418	0.0687	0.3139
NIT1	Nitrilase homolog 1	Q86X76	0.3492	24.4021	−2.2955	0.0687	0.3139
PMM2	Phosphomannomutase 2	O15305	−0.3006	25.6986	2.2021	0.0689	0.3139
DOPD	D-dopachrome decarboxylase	P30046	0.3954	25.3406	−2.1388	0.0690	0.3139
CAPZB	F-actin-capping protein subunit beta	P47756	−0.3492	28.1353	2.1368	0.0692	0.3140
DDAH1	N(G),N(G)-dimethylarginine dimethylaminohydrolase 1	O94760	−0.4587	25.0918	2.1315	0.0698	0.3141
PCBP2	Poly(rC)-binding protein 2	Q15366	−0.4798	28.5545	2.1305	0.0699	0.3141
PUF60	FBP-interacting repressor	Q9UHX1	−0.1997	27.7785	2.1289	0.0700	0.3141
DYST	Dystonin	Q03001	−0.5899	24.6045	2.1900	0.0701	0.3141
HNRH1	Heterogeneous nuclear ribonucleoprotein H	P31943	−0.2657	27.3964	2.1261	0.0703	0.3141
EMAL4	Echinoderm microtubule-associated protein-like 4	Q9HC35	−0.2998	28.3462	2.1259	0.0703	0.3141
TXLNG	Gamma-taxilin	Q9NUQ3	0.4078	23.9779	−2.2726	0.0708	0.3152
BRE1A	RING finger protein 20	Q5VTR2	0.4392	23.3280	−2.2706	0.0710	0.3153
GSTK1	Glutathione S-transferase kappa 1	Q9Y2Q3	0.4239	24.5594	−2.1729	0.0717	0.3175
SAMH1	Deoxynucleoside triphosphate triphosphohydrolase SAMHD1 (dNTPase)	Q9Y3Z3	0.3787	24.5239	−2.1711	0.0719	0.3175
PP14B	Protein phosphatase 1 regulatory subunit 14B	Q96C90	0.5532	25.7627	−2.1097	0.0720	0.3175
HNRPD	Heterogeneous nuclear ribonucleoprotein D0	Q14103	−0.2329	29.0843	2.1058	0.0725	0.3175
SYDC	Aspartate-tRNA ligase, cytoplasmic	P14868	0.2034	28.1939	−2.1052	0.0725	0.3175
COA4	Cytochrome c oxidase assembly factor 4 homolog, mitochondrial	Q9NYJ1	−0.4150	25.3470	2.2527	0.0726	0.3175
CPNS1	Calpain small subunit 1	P04632	−0.3130	28.3026	2.0998	0.0731	0.3191
DDX24	ATP-dependent RNA helicase DDX24	Q9GZR7	0.2690	23.4153	−2.1548	0.0736	0.3194
4F2	Solute carrier family 3 member 2	P08195	0.3987	27.2221	−2.0943	0.0737	0.3194
VATC1	V-type proton ATPase subunit C 1	P21283	0.5220	23.7171	−2.0923	0.0739	0.3194
DNJC9	DnaJ homolog subfamily C member 9	Q8WXX5	−0.3547	26.1825	2.0919	0.0740	0.3194
TBC15	TBC1 domain family member 15	Q8TC07	−0.2612	24.4707	2.0907	0.0741	0.3194
HNRL2	Heterogeneous nuclear ribonucleoprotein U-like protein 2	Q1KMD3	0.2147	27.2290	−2.0813	0.0752	0.3220
THIC	Acetyl-CoA acetyltransferase, cytosolic	Q9BWD1	0.2314	27.8881	−2.0788	0.0754	0.3220
TOM70	Translocase of outer membrane 70 kDa subunit	O94826	−0.3404	24.6368	2.0781	0.0755	0.3220
GDIB	Guanosine diphosphate dissociation inhibitor 2	P50395	0.2052	29.3586	−2.0767	0.0757	0.3220
PDS5A	Cell proliferation-inducing gene 54 protein	Q29RF7	−0.2315	25.9809	2.0765	0.0757	0.3220
DYHC1	Cytoplasmic dynein 1 heavy chain 1	Q14204	0.1869	30.2345	−2.0743	0.0760	0.3220
PRPS1	Phosphoribosyl pyrophosphate synthase I	P60891	−0.2485	27.9899	2.0736	0.0760	0.3220
EFHD2	EF-hand domain-containing protein D2	Q96C19	0.2612	26.2661	−2.0712	0.0763	0.3223
RS25	40S ribosomal protein S25	P62851	−0.2359	29.1488	2.0677	0.0767	0.3232
RL29	60S ribosomal protein L29	P47914	0.4223	28.8297	−2.1229	0.0769	0.3234
MESD	Mesoderm development candidate 2	Q14696	−0.2002	25.8762	2.0586	0.0777	0.3260

GAG2B	G antigen 2B/2C	Q13066	0.2886	29.0894	−2.0547	0.0782	0.3271
PTN23	Tyrosine-protein phosphatase non-receptor type 23	Q9H3S7	0.3476	23.8911	−2.0431	0.0796	0.3314
KIF11	Kinesin-like protein KIF11	P52732	0.3000	25.7141	−2.0414	0.0798	0.3314
PIR	Pirin	O00625	0.2424	25.0089	−2.0966	0.0798	0.3314
AN32B	Acidic leucine-rich nuclear phosphoprotein 32 family member B	Q92688	−0.3715	27.4780	2.0365	0.0803	0.3322
RAB18	Ras-related protein Rab-18	Q9NP72	−0.2496	25.6432	2.0358	0.0804	0.3322
CASP8	Caspase-8	Q14790	0.5834	23.8971	−2.0344	0.0806	0.3322
AGM1	Phosphoglucomutase-3	O95394	0.3383	25.7106	−2.0288	0.0813	0.3332
GLU2B	Protein kinase C substrate 60.1 kDa protein heavy chain	P14314	−0.2507	28.7318	2.0280	0.0814	0.3332
NAT10	N-acetyltransferase 10	Q9H0A0	−0.1970	27.0964	2.0273	0.0814	0.3332
AP3M1	AP-3 complex subunit mu-1	Q9Y2T2	−0.6280	24.5704	2.0793	0.0818	0.3332
RSSA	40S ribosomal protein SA	P08865	−0.2190	30.1401	2.0244	0.0818	0.3332
MANF	Mesencephalic astrocyte-derived neurotrophic factor	P55145	−0.3641	26.7060	2.0209	0.0822	0.3336
GSH0	Glutamate-cysteine ligase modifier subunit	P48507	0.3867	25.6860	−2.0747	0.0823	0.3336
NEP	Neprilysin	P08473	−0.3234	25.6504	2.0180	0.0826	0.3340
CCNB1	G2/mitotic-specific cyclin-B1	P14635	−0.3466	25.8396	2.0137	0.0831	0.3348
P5CS	Aldehyde dehydrogenase family 18 member A1	P54886	−0.1919	26.5579	2.0133	0.0832	0.3348
PYGL	Glycogen phosphorylase, liver form	P06737	0.2114	26.8926	−2.0116	0.0834	0.3349
SYVC	Valine-tRNA ligase	P26640	−0.1802	28.2899	2.0074	0.0839	0.3362
SF3B3	Splicing factor 3B subunit 3	Q15393	−0.2448	27.3110	2.0044	0.0843	0.3369
RBM39	RNA-binding protein 39	Q14498	−0.3155	25.3909	1.9953	0.0854	0.3399
KIF4A	Chromosome-associated kinesin KIF4A	O95239	−0.3884	25.9415	1.9942	0.0856	0.3399
DYR	Dihydrofolate reductase	P00374	−0.2820	26.4151	1.9938	0.0856	0.3399
GRPE1	GrpE protein homolog 1, mitochondrial	Q9HAV7	−0.2904	26.3556	1.9916	0.0859	0.3402
PPP5	Serine/threonine-protein phosphatase 5	P53041	−0.3086	24.9068	1.9877	0.0864	0.3414
IMB1	Importin subunit beta-1	Q14974	0.2509	29.8510	−1.9843	0.0868	0.3416
SRSF3	Serine/arginine-rich splicing factor 3	P84103	−0.4573	27.2398	1.9841	0.0868	0.3416
PSA	Puromycin-sensitive aminopeptidase	P55786	0.1961	27.6084	−1.9797	0.0874	0.3431
BOREA	Cell division cycle-associated protein 8	Q53HL2	−0.3391	23.8460	2.1055	0.0876	0.3431
ADAM9	Disintegrin and metalloproteinase domain-containing protein 9	Q13443	−0.2266	25.3889	1.9746	0.0881	0.3439
MCA3	Eukaryotic translation elongation factor 1 epsilon-1	O43324	−0.2095	26.1297	1.9734	0.0882	0.3439
H2AZ	Histone H2A.Z	P0C0S5	−0.5060	26.7516	1.9711	0.0885	0.3443
RL17	60S ribosomal protein L17	P18621	−0.3072	28.0652	1.9687	0.0889	0.3448
P3H1	Prolyl 3-hydroxylase 1	Q32P28	0.2241	24.9348	−1.9665	0.0891	0.3451
SYG	Glycine-tRNA ligase	P41250	0.2169	28.4349	−1.9642	0.0895	0.3455
XRCC6	X-ray repair cross-complementing protein 6	P12956	0.2013	29.4757	−1.9621	0.0897	0.3458
EIF3C	Eukaryotic translation initiation factor 3 subunit C	Q99613	0.1938	28.9202	−1.9513	0.0912	0.3500

SUCA	Succinyl-CoA synthetase subunit alpha	P53597	0.3664	25.5295	−2.0732	0.0913	0.3500
GCN1L	GCN1-like protein 1	Q92616	−0.2463	26.5055	1.9488	0.0915	0.3500
DHE3	Glutamate dehydrogenase 1, mitochondrial	P00367	0.3884	26.2850	−1.9464	0.0918	0.3500
BASI	Basigin	P35613	−0.2890	25.0268	1.9447	0.0921	0.3500
PALLD	Palladin	Q8WX93	−0.3278	25.8096	1.9444	0.0921	0.3500
PTMA	Prothymosin alpha	P06454	0.7954	25.6121	−1.9931	0.0922	0.3500
IDHC	Isocitrate dehydrogenase [NADP] cytoplasmic	O75874	0.2140	26.5468	−1.9368	0.0932	0.3518
RL31	60S ribosomal protein L31	P62899	0.2909	28.9282	−1.9838	0.0934	0.3518
VTA1	Vacuolar protein sorting-associated protein VTA1 homolog	Q9NP79	−0.2210	25.3948	1.9328	0.0937	0.3518
HS105	Heat shock protein 105 kDa	Q92598	−0.1934	29.1067	1.9323	0.0938	0.3518
ATX2L	Ataxin-2-like protein	Q8WWM7	−0.2411	26.5888	1.9304	0.0941	0.3518
UBQL1	Ubiquilin-1	Q9UMX0	0.3131	24.9270	−1.9300	0.0941	0.3518
DCNL1	DCN1-like protein 1	Q96GG9	0.5258	23.5576	−1.9281	0.0944	0.3518
SC23B	Protein transport protein Sec23B	Q15437	0.2501	24.8595	−1.9273	0.0945	0.3518
RS4X	40S ribosomal protein S4, X isoform	P62701	−0.3531	29.9937	1.9257	0.0947	0.3518
RS23	40S ribosomal protein S23	P62266	−0.2663	27.3882	1.9252	0.0948	0.3518
DPYL3	Dihydropyrimidinase-related protein 3	Q14195	0.2451	25.0007	−1.9196	0.0956	0.3540
SC22B	Vesicle-trafficking protein SEC22b	O75396	0.2704	27.6113	−1.9178	0.0958	0.3542
UBFD1	Ubiquitin domain-containing protein UBFD1	O14562	−0.3682	25.1398	1.9627	0.0962	0.3550
ZN326	DBIRD complex subunit ZNF326	Q5BKZ1	0.2291	24.6978	−1.9127	0.0966	0.3551
MOES	Moesin	P26038	0.1932	29.4326	−1.9084	0.0972	0.3551
EIF3J	Eukaryotic translation initiation factor 3 subunit J	O75822	0.2054	27.8689	−1.9078	0.0973	0.3551
ESYT1	Extended synaptotagmin-1	Q9BSJ8	0.1769	27.2152	−1.9077	0.0973	0.3551
TTC37	TPR repeat protein 37	Q6PGP7	−0.2595	23.4098	1.9547	0.0973	0.3551
TATD1	Putative deoxyribonuclease TATDN1	Q6P1N9	−0.2481	25.2759	1.9059	0.0975	0.3551
U5S1	Elongation factor Tu GTP-binding domain-containing protein 2	Q15029	0.2171	28.3141	−1.9028	0.0980	0.3558
GNS	N-acetylglucosamine-6-sulfatase	P15586	−0.2636	25.4877	1.9000	0.0984	0.3558
OGT1	O-GlcNAc transferase subunit p110	O15294	−0.3155	24.6842	1.8994	0.0985	0.3558
SSRD	Signal sequence receptor subunit delta	P51571	0.2395	26.0935	−1.8989	0.0986	0.3558
CMC2	Solute carrier family 25 member 13	Q9UJS0	−0.4437	25.6161	1.8966	0.0989	0.3563
YAP1	Yes-associated protein 1	P46937	0.5951	26.1335	−1.8880	0.1002	0.3587
CCAR2	Cell cycle and apoptosis regulator protein 2	Q8N163	−0.2469	26.4862	1.8878	0.1002	0.3587
AGFG1	Arf-GAP domain and FG repeat-containing protein 1	P52594	0.3359	24.8559	−2.0016	0.1002	0.3587
EP15R	Epidermal growth factor receptor substrate 15-like 1	Q9UBC2	−0.1934	23.2753	1.8851	0.1006	0.3594
ZC3HF	Zinc finger CCCH domain-containing protein 15	Q8WU90	−0.6115	24.7475	1.9286	0.1009	0.3594
AB1IP	Retinoic acid-responsive proline-rich protein 1	Q7Z5R6	−0.1766	25.9204	1.8816	0.1011	0.3594
CSN2	COP9 signalosome complex subunit 2	P61201	−0.2440	25.0138	1.9265	0.1012	0.3594

TIGAR	TP53-induced glycolysis and apoptosis regulator	Q9NQ88	−0.3942	23.9020	1.9921	0.1014	0.3594
CCAR1	Cell cycle and apoptosis regulator protein 1	Q8IX12	−0.3943	24.4096	1.9224	0.1018	0.3600
COX5B	Cytochrome c oxidase subunit 5B, mitochondrial	P10606	−0.3718	26.3051	1.8738	0.1023	0.3603
DAAF5	Dynein assembly factor 5, axonemal	Q86Y56	0.3787	23.7217	−1.9853	0.1023	0.3603
SIAS	N-acetylneuraminate synthase	Q9NR45	−0.2103	25.7378	1.8714	0.1026	0.3607
IPO4	Importin-4	Q8TEX9	0.2193	25.8788	−1.8701	0.1028	0.3607
U2AF1	U2 small nuclear RNA auxiliary factor 1	Q01081	0.5615	25.4838	−1.9132	0.1031	0.3610
CDC73	Cell division cycle protein 73 homolog	Q6P1J9	−0.1858	24.9248	1.8591	0.1045	0.3651
MIMIT	Mimitin, mitochondrial	Q8N183	−0.2398	24.4427	1.8977	0.1054	0.3668
UBE4B	Ubiquitin conjugation factor E4 B	O95155	−0.4490	24.2501	1.8531	0.1055	0.3668
CSTF3	Cleavage stimulation factor subunit 3	Q12996	−0.8945	24.8252	1.9551	0.1064	0.3688
ARC1B	Actin-related protein 2/3 complex subunit 1B	O15143	0.3734	26.7114	−1.8468	0.1064	0.3688
LPPRC	Leucine-rich PPR motif-containing protein, mitochondrial	P42704	−0.1877	28.6634	1.8442	0.1069	0.3693
KAP0	cAMP-dependent protein kinase type I-alpha regulatory subunit	P10644	0.3051	26.7281	−1.8431	0.1070	0.3693
KIF23	Kinesin-like protein KIF23	Q02241	−0.2175	25.8359	1.8405	0.1074	0.3694
NHLC2	NHL repeat-containing protein 2	Q8NBF2	−0.2444	24.1560	1.8837	0.1075	0.3694
VPS29	Vacuolar protein sorting-associated protein 29	Q9UBQ0	−0.3282	27.3362	1.8365	0.1081	0.3707
RPAC1	DNA-directed RNA polymerases I and III subunit RPAC1	O15160	−0.2405	25.6611	1.8328	0.1087	0.3719
PCBP1	Poly(rC)-binding protein 1	Q15365	−0.2100	29.6529	1.8316	0.1089	0.3719
LASP1	LIM and SH3 domain protein 1	Q14847	0.1890	28.7612	−1.8245	0.1100	0.3751
CELF1	CUGBP Elav-like family member 1	Q92879	−0.4437	25.9720	1.9247	0.1107	0.3763
CHERP	Calcium homeostasis endoplasmic reticulum protein	Q8IWX8	−0.4307	23.7379	1.8611	0.1109	0.3763
PSME3	Proteasome activator complex subunit 3	P61289	0.1903	26.8209	−1.8169	0.1113	0.3763
ACOC	Cytoplasmic aconitate hydratase	P21399	0.2782	25.2071	−1.8167	0.1113	0.3763
PGRC1	Membrane-associated progesterone receptor component 1	O00264	0.2944	25.7550	−1.8567	0.1116	0.3763
NUP53	Nucleoporin Nup35	Q8NFH5	−0.2495	25.2707	1.8143	0.1117	0.3763
MAVS	Mitochondrial antiviral-signaling protein	Q7Z434	−0.1854	26.1970	1.8124	0.1120	0.3766
NIBL1	Melanoma invasion by ERK	Q96TA1	0.2556	25.8564	−1.8109	0.1122	0.3767
ACTZ	Alpha-centractin	P61163	−0.3707	26.9519	1.8055	0.1131	0.3783
HUWE1	ARF-binding protein 1	Q7Z6Z7	−0.2511	26.1177	1.8054	0.1132	0.3783
DDX1	ATP-dependent RNA helicase DDX1	Q92499	−0.1765	27.2271	1.7994	0.1142	0.3809
LARP1	La-related protein 1	Q6PKG0	−0.1794	26.6498	1.7939	0.1151	0.3832
RL23A	60S ribosomal protein L23a	P62750	−0.2379	29.6061	1.7922	0.1154	0.3834
WDR36	WD repeat-containing protein 36	Q8NI36	0.2010	24.6693	−1.7889	0.1159	0.3841
DENR	Density-regulated protein	O43583	−0.5615	25.4019	1.7885	0.1160	0.3841
LPP	Lipoma-preferred partner	Q93052	−0.2807	24.3255	1.8257	0.1166	0.3841

FXR1	Fragile X mental retardation syndrome-related protein 1	P51114	−0.3787	24.6923	1.7840	0.1168	0.3841
SYHC	Histidine-tRNA ligase, cytoplasmic	P12081	0.2124	26.9161	−1.7839	0.1168	0.3841
TRM6	tRNA(m1A58)MTase subunit TRM6	Q9UJA5	−0.2252	25.5992	1.7821	0.1171	0.3841
RS9	40S ribosomal protein S9	P46781	−0.2367	30.1481	1.7819	0.1171	0.3841
RL19	60S ribosomal protein L19	P84098	−0.3521	28.2346	1.7807	0.1173	0.3841
SYCC	Cysteinyl-tRNA synthetase	P49589	−0.3560	25.2172	1.7788	0.1177	0.3844
TIF1B	Tripartite motif-containing protein 28	Q13263	0.1761	29.7871	−1.7774	0.1179	0.3845
PDIA4	Protein disulfide-isomerase A4	P13667	−0.1652	29.0749	1.7750	0.1183	0.3851
DDX23	Probable ATP-dependent RNA helicase DDX23	Q9BUQ8	0.1981	25.4529	−1.7686	0.1195	0.3880
AT1B1	Sodium/potassium-transporting ATPase subunit beta-1	P05026	−0.2064	26.1951	1.7645	0.1202	0.3896
HGH1	Protein HGH1 homolog	P0CB43	0.2662	24.3923	−1.7622	0.1206	0.3898
ARL3	ADP-ribosylation factor-like protein 3	P36405	0.2527	26.0598	−1.7608	0.1208	0.3898
GEMI5	Gem-associated protein 5	Q8TEQ6	0.1939	26.7263	−1.7585	0.1212	0.3898
PTBP1	Polypyrimidine tract-binding protein 1	P26599	−0.2247	28.5023	1.7575	0.1214	0.3898
NASP	Nuclear autoantigenic sperm protein	P49321	0.1909	28.7027	−1.7571	0.1215	0.3898
TIM50	Mitochondrial import inner membrane translocase subunit TIM50	Q3ZCQ8	0.3009	24.8954	−1.7955	0.1216	0.3898
UBXN4	UBX domain-containing protein 4	Q92575	−0.4533	23.8404	1.7522	0.1224	0.3906
THOP1	Thimet oligopeptidase	P52888	0.2436	25.1248	−1.7508	0.1226	0.3906
CYTB	Cystatin-B	P04080	−0.3047	28.8285	1.7487	0.1230	0.3906
CH60	Heat shock protein 60	P10809	−0.1727	31.9471	1.7483	0.1231	0.3906
ARPC2	Actin-related protein 2/3 complex subunit 2	O15144	0.1939	26.5106	−1.7482	0.1231	0.3906
MAP11	Methionine aminopeptidase 1	P53582	−0.2740	25.7364	1.7475	0.1232	0.3906
RS2	40S ribosomal protein S2	P15880	−0.2080	29.9921	1.7426	0.1241	0.3922
EF1B	Elongation factor 1-beta	P24534	−0.3622	28.2857	1.7402	0.1245	0.3922
ROAA	Heterogeneous nuclear ribonucleoprotein A/B	Q99729	1.1749	24.9187	−1.8335	0.1246	0.3922
CD2B2	CD2 tail-binding protein 2	O95400	0.1955	24.0719	−1.7778	0.1246	0.3922
RNPS1	RNA-binding protein with serine-rich domain 1	Q15287	−0.2533	25.3024	1.8238	0.1262	0.3954
PSMD8	26S proteasome non-ATPase regulatory subunit 8	P48556	0.1600	27.1215	−1.7306	0.1263	0.3954
TRAP1	Heat shock protein 75 kDa, mitochondrial	Q12931	−0.1955	27.6521	1.7304	0.1263	0.3954
MCM3	DNA replication licensing factor MCM3	P25205	0.1606	28.3790	−1.7253	0.1273	0.3970
ACTG	Actin, cytoplasmic 2	P63261	0.1876	28.1256	−1.7251	0.1273	0.3970
LGUL	Glyoxalase I	Q04760	−0.4025	26.7428	1.7177	0.1287	0.4000
MTCH2	Mitochondrial carrier homolog 2	Q9Y6C9	0.2331	27.0816	−1.7175	0.1288	0.4000
UFD1	Ubiquitin fusion degradation protein 1 homolog	Q92890	−0.1824	25.9727	1.7152	0.1292	0.4001
HSPB1	Heat shock protein beta-1	P04792	0.1730	28.4297	−1.7130	0.1296	0.4001
FAHD1	Fumarylacetoacetate hydrolase domain-containing protein 1	Q6P587	0.5569	25.1928	−1.7128	0.1296	0.4001
TBK1	Serine/threonine-protein kinase TBK1	Q9UHD2	−0.4274	23.2271	1.7492	0.1297	0.4001

F10A1	Hsc70-interacting protein	P50502	0.2374	27.8868	−1.7083	0.1305	0.4018
SC16A	SEC16 homolog A	O15027	−0.3505	22.6835	1.7052	0.1311	0.4029
SLIRP	SRA stem-loop-interacting RNA-binding protein, mitochondrial	Q9GZT3	−0.5588	26.0214	1.7356	0.1322	0.4044
FIP1	Factor interacting with PAP	Q6UN15	−0.1781	25.8481	1.6994	0.1322	0.4044
HERC4	Probable E3 ubiquitin-protein ligase HERC4	Q5GLZ8	−0.2338	25.1022	1.6990	0.1323	0.4044
RS18	40S ribosomal protein S18	P62269	−0.2242	30.2322	1.6923	0.1336	0.4059
GBB1	Guanine nucleotide-binding protein G(I)/G(S)/G(T) subunit beta-1	P62873	−0.2102	27.3467	1.6920	0.1336	0.4059
SPF30	Survival motor neuron domain-containing protein 1	O75940	−0.1899	24.5180	1.7276	0.1337	0.4059
TBA1C	Tubulin alpha-1C chain	Q9BQE3	0.3092	25.4911	−1.6917	0.1337	0.4059
2AAA	PP2A subunit A isoform R1-alpha	P30153	−0.1603	29.2503	1.6885	0.1343	0.4070
UB2L3	Ubiquitin-conjugating enzyme E2 L3	P68036	0.2432	27.7879	−1.6868	0.1347	0.4072
USP9X	Ubiquitin-specific protease 9, X chromosome	Q93008	0.2494	24.7246	−1.6857	0.1349	0.4072
LAP2B	Thymopoietin-related peptide isoforms beta/gamma	P42167	−0.2090	25.8783	1.6822	0.1356	0.4086
ABI1	Abelson interactor 1	Q8IZP0	−0.3143	23.8000	1.7126	0.1365	0.4107
KINH	Kinesin-1 heavy chain	P33176	−0.1821	27.7716	1.6732	0.1374	0.4126
SMCA5	Sucrose nonfermenting protein 2 homolog	O60264	0.2931	25.4206	−1.6720	0.1376	0.4126
AHSA1	Activator of 90 kDa heat shock protein ATPase homolog 1	O95433	0.2577	27.0862	−1.6697	0.1381	0.4133
CIAO1	Probable cytosolic iron-sulfur protein assembly protein CIAO1	O76071	0.4947	24.4231	−1.6676	0.1385	0.4133
RL5	60S ribosomal protein L5	P46777	−0.1779	29.9415	1.6672	0.1386	0.4133
FHL2	Four and a half LIM domains protein 2	Q14192	−0.4312	25.5813	1.6582	0.1404	0.4176
HNRH3	Heterogeneous nuclear ribonucleoprotein H3	P31942	0.1734	26.6226	−1.6577	0.1405	0.4176
VRK1	Vaccinia-related kinase 1	Q99986	−0.2090	24.2032	1.6535	0.1414	0.4192
NUP88	Nucleoporin Nup88	Q99567	0.3962	23.9498	−1.6867	0.1415	0.4192
SAP	Prosaposin	P07602	−0.2054	28.9166	1.6482	0.1425	0.4213
SNAA	Alpha-soluble NSF attachment protein	P54920	0.1901	26.7898	−1.6453	0.1431	0.4217
MCMBP	Mini-chromosome maintenance complex-binding protein	Q9BTE3	−0.3382	24.6223	1.6447	0.1432	0.4217
ZCCHV	Zinc finger CCCH-type antiviral protein 1	Q7Z2W4	−0.2049	24.9681	1.6439	0.1434	0.4217
PCP	Prolylcarboxypeptidase	P42785	−0.2293	23.6463	1.7232	0.1439	0.4225
DFFA	DNA fragmentation factor subunit alpha	O00273	−0.3643	24.8409	1.6364	0.1449	0.4243
SCMC1	Solute carrier family 25 member 24	Q6NUK1	0.3932	23.7172	−1.6361	0.1450	0.4243
SCPDL	Saccharopine dehydrogenase-like oxidoreductase	Q8NBX0	−0.2935	23.9963	1.6667	0.1455	0.4245
GLRX5	Glutaredoxin-related protein 5, mitochondrial	Q86SX6	0.3247	23.6112	−1.6335	0.1455	0.4245
OST48	Dolichyl-diphosphooligosaccharide-protein glycosyltransferase 48 kDa subunit	P39656	0.2790	25.4484	−1.6650	0.1459	0.4247

TPD54	Tumor protein D52-like 2	O43399	0.1967	28.2150	−1.6298	0.1463	0.4251
NED4L	E3 ubiquitin-protein ligase NEDD4-like	Q96PU5	−0.3611	23.4550	1.7094	0.1465	0.4251
ECI2	Enoyl-CoA delta isomerase 2, mitochondrial	O75521	0.3998	24.6208	−1.6239	0.1476	0.4276
NSF	N-ethylmaleimide-sensitive fusion protein	P46459	0.2828	25.5263	−1.6183	0.1488	0.4303
PRS6B	Tat-binding protein 7	P43686	0.1731	28.1395	−1.6167	0.1491	0.4306
K1C18	Cytokeratin-18	P05783	0.1605	32.3138	−1.6149	0.1495	0.4310
SF3A1	Splicing factor 3A subunit 1	Q15459	−0.1498	27.7274	1.6135	0.1498	0.4312
SP16H	Facilitates chromatin transcription complex subunit SPT16	Q9Y5B9	0.1441	27.9963	−1.6120	0.1502	0.4314
NAA15	N-alpha-acetyltransferase 15, NatA auxiliary subunit	Q9BXJ9	0.1805	28.0393	−1.6078	0.1511	0.4333
DHB4	17-beta-hydroxysteroid dehydrogenase 4	P51659	0.1588	27.5546	−1.6059	0.1515	0.4336
FRIH	Ferritin heavy chain	P02794	0.3239	24.3072	−1.6354	0.1520	0.4336
LBR	Lamin-B receptor	Q14739	−0.2473	25.6369	1.6349	0.1521	0.4336
UGGG1	UDP-glucose:glycoprotein glucosyltransferase 1	Q9NYU2	0.3065	23.8432	−1.6010	0.1526	0.4336
FSCN1	Fascin	Q16658	0.3227	26.4857	−1.5985	0.1531	0.4336
C2AIL	CDKN2AIP N-terminal-like protein	Q96HQ2	0.3150	24.7316	−1.6751	0.1532	0.4336
TTL12	Tubulin--tyrosine ligase-like protein 12	Q14166	0.2184	24.9025	−1.5982	0.1532	0.4336
GSTP1	Glutathione S-transferase P	P09211	0.1466	29.8808	−1.5982	0.1532	0.4336
LA	Sjoegren syndrome type B antigen	P05455	0.2090	28.4548	−1.5961	0.1537	0.4339
CDC37	Hsp90 co-chaperone Cdc37	Q16543	−0.1602	27.2737	1.5954	0.1538	0.4339
RS16	40S ribosomal protein S16	P62249	−0.1507	29.4468	1.5937	0.1542	0.4342
PTBP2	Polypyrimidine tract-binding protein 2	Q9UKA9	−0.8900	24.3796	1.5913	0.1547	0.4350
ABCF2	ATP-binding cassette sub-family F member 2	Q9UG63	0.1527	26.4698	−1.5893	0.1552	0.4350
TOM22	Translocase of outer membrane 22 kDa subunit homolog	Q9NS69	0.2145	27.1433	−1.5891	0.1552	0.4350
CCD47	Coiled-coil domain-containing protein 47	Q96A33	−0.1986	25.9009	1.5871	0.1557	0.4356
SUMO1	Small ubiquitin-related modifier 1	P63165	−0.4250	25.3205	1.6602	0.1562	0.4362
NUDC2	NudC domain-containing protein 2	Q8WVJ2	0.2463	25.8593	−1.5822	0.1568	0.4368
PNPH	Purine nucleoside phosphorylase	P00491	0.2534	26.2797	−1.5818	0.1569	0.4368
VIGLN	HDL-binding protein	Q00341	−0.1505	27.0126	1.5764	0.1581	0.4394
PDIP3	Polymerase delta-interacting protein 3	Q9BY77	−0.1860	24.0369	1.5738	0.1587	0.4404
M2OM	Solute carrier family 25 member 11	Q02978	−0.1845	25.4566	1.5726	0.1590	0.4405
EMD	Emerin	P50402	0.2429	27.0068	−1.5677	0.1601	0.4416
ETFB	Electron transfer flavoprotein subunit beta	P38117	0.1900	26.9765	−1.5672	0.1602	0.4416
ADT3	Solute carrier family 25 member 6	P12236	−0.3918	25.1161	1.6400	0.1603	0.4416
TCEA1	Transcription elongation factor A protein 1	P23193	−0.1489	26.3660	1.5661	0.1605	0.4416
BACH	Acyl-CoA thioesterase 7	O00154	0.2122	26.7043	−1.5651	0.1607	0.4416
PGP	Phosphoglycolate phosphatase	A6NDG6	0.7419	24.7192	−1.5940	0.1609	0.4416
NDKB	Nucleoside diphosphate kinase B	P22392	−0.2892	27.2839	1.5905	0.1617	0.4430

PGK1	Phosphoglycerate kinase 1	P00558	−0.1756	31.4703	1.5558	0.1629	0.4451
GALK1	Galactokinase (EC 2.7.1.6) (Galactose kinase)	P51570	0.3023	25.5726	−1.5542	0.1633	0.4451
ZPR1	Zinc finger protein ZPR1	O75312	0.1498	25.7897	−1.5536	0.1634	0.4451
APT	Adenine phosphoribosyltransferase	P07741	0.3257	25.8355	−1.5531	0.1635	0.4451
PRP6	Pre-mRNA-processing factor 6	O94906	0.2550	25.2666	−1.5488	0.1645	0.4472
RANG	Ran-binding protein 1	P43487	0.1805	28.0516	−1.5385	0.1670	0.4531
SPT5H	Transcription elongation factor SPT5	O00267	0.2507	26.1457	−1.5337	0.1681	0.4552
RL4	60S ribosomal protein L4	P36578	−0.1676	30.5490	1.5324	0.1685	0.4552
NUFP2	Nuclear fragile X mental retardation-interacting protein 2	Q7Z417	−0.1499	25.6611	1.5321	0.1685	0.4552
DNJC5	DnaJ homolog subfamily C member 5	Q9H3Z4	−0.1942	24.8991	1.5577	0.1692	0.4563
SPCS2	Signal peptidase complex subunit 2	Q15005	−0.2709	24.9807	1.5907	0.1710	0.4603
1433E	14-3-3 protein epsilon	P62258	0.1560	30.7284	−1.5192	0.1717	0.4612
RHOG	Rho-related GTP-binding protein RhoG	P84095	0.1938	25.7360	−1.5176	0.1721	0.4612
RAB1A	Ras-related protein Rab-1A	P62820	0.1714	24.7459	−1.5175	0.1721	0.4612
SPAS2	Spermatogenesis-associated serine-rich protein 2	Q86XZ4	−0.1663	23.5374	1.5121	0.1734	0.4630
CRK	Adapter molecule crk	P46108	−0.1685	23.9034	1.5120	0.1735	0.4630
NOL9	Nucleolar protein 9	Q5SY16	0.3046	23.2971	−1.5391	0.1736	0.4630
YIF1B	Protein YIF1B	Q5BJH7	0.2854	24.1556	−1.5343	0.1747	0.4653
PSA6	Proteasome subunit alpha type-6	P60900	−0.3293	27.7098	1.5038	0.1755	0.4665
CKAP4	Cytoskeleton-associated protein 4	Q07065	0.2477	27.8971	−1.5030	0.1757	0.4665
RCC1	Regulator of chromosome condensation	P18754	0.2250	26.7850	−1.4995	0.1766	0.4670
LAS1L	Ribosomal biogenesis protein LAS1L	Q9Y4W2	−0.1854	24.8103	1.4990	0.1767	0.4670
S38A2	Solute carrier family 38 member 2	Q96QD8	−0.3219	26.0797	1.4980	0.1770	0.4670
RBPMS	RNA-binding protein with multiple splicing	Q93062	−0.2455	26.8092	1.4979	0.1770	0.4670
EIF3I	Eukaryotic translation initiation factor 3 subunit I	Q13347	0.2778	28.0802	−1.4955	0.1776	0.4673
ARPC4	Actin-related protein 2/3 complex subunit 4	P59998	0.2433	25.8616	−1.4954	0.1776	0.4673
TM9S4	Transmembrane 9 superfamily member 4	Q92544	−0.2410	25.2091	1.5210	0.1780	0.4674
C1QBP	Hyaluronan-binding protein 1	Q07021	0.3494	26.8359	−1.4891	0.1793	0.4700
ATX2	Ataxin-2	Q99700	−0.2346	23.4097	1.5126	0.1800	0.4700
PURA2	Adenylosuccinate synthetase isozyme 2	P30520	0.1483	25.9271	−1.4859	0.1801	0.4700
METK2	Methionine adenosyltransferase II	P31153	−0.2012	27.9632	1.4851	0.1803	0.4700
AL9A1	Aldehyde dehydrogenase family 9 member A1	P49189	0.2262	26.6285	−1.4849	0.1803	0.4700
SC61B	Protein transport protein Sec61 subunit beta	P60468	0.5845	25.5748	−1.5076	0.1813	0.4717
FABP5	Fatty acid-binding protein 5	Q01469	−0.6671	28.9540	1.4801	0.1816	0.4718
CPSF6	Cleavage and polyadenylation specificity factor subunit 6	Q16630	0.2499	25.2636	−1.4770	0.1824	0.4732
CYBP	Calcyclin-binding protein	Q9HB71	0.1714	28.5628	−1.4755	0.1828	0.4732
CNDP2	Cytosolic non-specific dipeptidase	Q96KP4	0.1345	27.7672	−1.4748	0.1829	0.4732
RAB6A	Ras-related protein Rab-6A	P20340	0.1776	28.3136	−1.4705	0.1841	0.4748
UBA6	Ubiquitin-activating enzyme 6	A0AVT1	−0.2451	24.1064	1.4955	0.1843	0.4748

DPP3	Dipeptidyl peptidase 3	Q9NY33	0.1738	26.1230	−1.4693	0.1844	0.4748
PABP1	Poly(A)-binding protein 1	P11940	−0.1932	29.7040	1.4661	0.1852	0.4762
RD23B	UV excision repair protein RAD23 homolog B	P54727	−0.1932	27.9724	1.4646	0.1856	0.4766
UGPA	UDP-glucose pyrophosphorylase	Q16851	−0.2735	24.8843	1.4882	0.1862	0.4767
FA49B	Protein FAM49B	Q9NUQ9	−0.2080	23.7292	1.4880	0.1862	0.4767
PP1B	Serine/threonine-protein phosphatase PP1-beta catalytic subunit	P62140	0.1854	26.0157	−1.4560	0.1879	0.4795
HEAT3	HEAT repeat-containing protein 3	Q7Z4Q2	0.1656	24.0953	−1.4554	0.1881	0.4795
UBA3	Ubiquitin-activating enzyme 3	Q8TBC4	0.3752	24.9920	−1.4550	0.1882	0.4795
XPO1	Exportin-1	O14980	0.2006	27.3077	−1.4532	0.1887	0.4798
DDX27	Probable ATP-dependent RNA helicase DDX27	Q96GQ7	−0.1462	26.2746	1.4525	0.1888	0.4798
U2AF2	U2 auxiliary factor 65 kDa subunit	P26368	0.1733	28.1816	−1.4495	0.1897	0.4812
NUDC	Nuclear distribution protein C homolog	Q9Y266	0.2027	29.3550	−1.4483	0.1900	0.4813
EHD2	EH domain-containing protein 2	Q9NZN4	0.1496	26.8442	−1.4452	0.1908	0.4827
NDKA	Nucleoside diphosphate kinase A	P15531	−0.1977	30.1799	1.4342	0.1938	0.4883
FUBP1	Far upstream element-binding protein 1	Q96AE4	−0.1557	28.8448	1.4340	0.1939	0.4883
NIT2	Nitrilase homolog 2	Q9NQR4	0.2040	24.7297	−1.4303	0.1949	0.4883
CAN1	Calcium-activated neutral proteinase 1	P07384	−0.2231	26.1736	1.4296	0.1951	0.4883
CAND1	Cullin-associated NEDD8-dissociated protein 1	Q86VP6	0.1406	28.8244	−1.4293	0.1952	0.4883
NOP56	Nucleolar protein 56	O00567	−0.2788	28.0373	1.4289	0.1953	0.4883
RBP2	Nucleoporin Nup358	P49792	0.2253	24.7995	−1.4283	0.1954	0.4883
ACTN1	Alpha-actinin-1	P12814	0.1417	29.2815	−1.4283	0.1955	0.4883
CS043	Uncharacterized protein C19orf43	Q9BQ61	−0.1947	24.7627	1.4862	0.1958	0.4883
QCR6	Ubiquinol-cytochrome c reductase complex 11 kDa protein	P07919	−0.1492	25.2305	1.4260	0.1961	0.4883
PEPD	Peptidase D	P12955	−0.3284	25.6342	1.4257	0.1962	0.4883
NOMO2	Nodal modulator 2	Q5JPE7	0.2265	24.2863	−1.4235	0.1968	0.4884
PRDX3	Peroxiredoxin III	P30048	0.2245	28.5878	−1.4223	0.1971	0.4884
BAG2	Bcl-2-associated athanogene 2	O95816	0.1881	26.1330	−1.4214	0.1974	0.4884
ZN638	Zinc finger protein 638	Q14966	−0.2426	23.5870	1.4207	0.1976	0.4884
BGH3	Transforming growth factor-beta-induced protein ig-h3	Q15582	0.1689	25.8725	−1.4201	0.1977	0.4884
GBF1	Golgi-specific brefeldin A-resistance guanine nucleotide exchange factor 1	Q92538	−0.2664	25.5911	1.4192	0.1980	0.4884
U3IP2	U3 snoRNP-associated 55 kDa protein	O43818	−0.1928	25.3577	1.4178	0.1984	0.4884
BAF	Barrier-to-autointegration factor	O75531	−0.8508	27.0205	1.4174	0.1985	0.4884
MCM6	DNA replication licensing factor MCM6	Q14566	−0.1548	27.5919	1.4123	0.1999	0.4913
P4HA1	Prolyl 4-hydroxylase subunit alpha-1	P13674	−0.1822	24.1829	1.4104	0.2005	0.4913
IPO7	Importin-7	O95373	−0.1365	28.1339	1.4102	0.2005	0.4913
RAB1B	Ras-related protein Rab-1B	Q9H0U4	0.1848	27.1133	−1.4074	0.2013	0.4925
MSH2	DNA mismatch repair protein Msh2	P43246	0.1927	26.8949	−1.4019	0.2029	0.4957
AASD1	Alanyl-tRNA synthetase domain-containing protein 1	Q9BTE6	−0.3070	23.6667	1.3999	0.2035	0.4963
DPYL2	Dihydropyrimidinase-related protein 2	Q16555	0.1425	29.5197	−1.3969	0.2043	0.4963
EF1G	Elongation factor 1-gamma	P26641	0.1443	30.6424	−1.3966	0.2044	0.4963

NOG1	Nucleolar GTP-binding protein 1	Q9BZE4	−0.2247	25.8789	1.3961	0.2046	0.4963
SAR1B	GTP-binding protein SAR1b	Q9Y6B6	0.2930	26.4420	−1.4190	0.2046	0.4963
UFM1	Ubiquitin-fold modifier 1	P61960	−0.1509	25.9024	1.3928	0.2055	0.4975
LYPA2	Lysophospholipase II	O95372	0.4338	24.2785	−1.4153	0.2057	0.4975
FUS	RNA-binding protein FUS	P35637	0.1631	27.7029	−1.3863	0.2074	0.5010
COR1B	Coronin-1B	Q9BR76	0.2179	27.0043	−1.3843	0.2080	0.5017
MXRA7	Matrix-remodeling-associated protein 7	P84157	0.2037	24.3196	−1.3813	0.2089	0.5017
COPB2	Coatomer subunit beta'	P35606	0.3013	26.8381	−1.3809	0.2090	0.5017
PIPNB	Phosphatidylinositol transfer protein beta isoform	P48739	−0.4172	26.5622	1.3799	0.2093	0.5017
SET	Protein SET	Q01105	0.3172	28.2084	−1.3792	0.2095	0.5017
PICAL	Phosphatidylinositol-binding clathrin assembly protein	Q13492	0.2890	25.5339	−1.3788	0.2096	0.5017
RBM22	RNA-binding motif protein 22	Q9NW64	−0.1749	23.9790	1.4008	0.2097	0.5017
NP1L4	Nucleosome assembly protein 1-like 4	Q99733	0.1580	28.0778	−1.3726	0.2114	0.5050
WBP2	WW domain-binding protein 2	Q969T9	0.2598	27.3608	−1.3693	0.2124	0.5058
ECHM	Enoyl-CoA hydratase 1	P30084	0.1761	27.8870	−1.3688	0.2126	0.5058
NAA50	N-alpha-acetyltransferase 50	Q9GZZ1	0.5447	25.9986	−1.3686	0.2126	0.5058
SYNE1	Synaptic nuclear envelope protein 1	Q8NF91	0.4248	26.0210	−1.3895	0.2130	0.5059
CLU	Clustered mitochondria protein homolog	O75153	−0.1545	25.5804	1.3645	0.2139	0.5073
DDX5	Probable ATP-dependent RNA helicase DDX5	P17844	0.1490	28.5539	−1.3610	0.2149	0.5091
40057	Septin-9	Q9UHD8	0.1460	28.3371	−1.3562	0.2164	0.5118
ESTD	Esterase D	P10768	−0.1934	28.9696	1.3540	0.2170	0.5127
XRN2	5'-3' exoribonuclease 2	Q9H0D6	−0.1330	26.6149	1.3528	0.2174	0.5128
ATP5H	ATPase subunit d	O75947	0.1597	27.1976	−1.3519	0.2177	0.5128
HKDC1	Hexokinase domain-containing protein 1	Q2TB90	−0.3342	26.3428	1.3707	0.2185	0.5130
VATA	V-ATPase subunit A	P38606	0.1849	26.3417	−1.3489	0.2186	0.5130
DP13A	DCC-interacting protein 13-alpha	Q9UKG1	0.1907	24.2983	−1.3700	0.2187	0.5130
GDIR1	Rho GDP-dissociation inhibitor 1	P52565	−0.2341	27.8471	1.3476	0.2190	0.5130
KAP2	cAMP-dependent protein kinase type II-alpha regulatory subunit	P13861	−0.2018	26.0531	1.3460	0.2195	0.5135
CHTOP	Chromatin target of PRMT1 protein	Q9Y3Y2	0.4132	25.4937	−1.3446	0.2199	0.5138
FA98B	Protein FAM98B	Q52LJ0	−0.1556	24.9714	1.3433	0.2203	0.5140
FLNC	Filamin-C	Q14315	−0.1887	31.9128	1.3418	0.2208	0.5141
SAC1	Suppressor of actin mutations 1-like protein	Q9NTJ5	−0.2550	24.4858	1.3624	0.2209	0.5141
NOLC1	Nucleolar and coiled-body phosphoprotein 1	Q14978	0.1948	26.8203	−1.3388	0.2217	0.5146
TBB3	Tubulin beta-3 chain	Q13509	−0.3713	27.9660	1.3386	0.2218	0.5146
EFTS	Elongation factor Ts, mitochondrial	P43897	−0.2097	25.3538	1.3561	0.2228	0.5153
DCTN2	Dynactin subunit 2	Q13561	0.1552	27.6691	−1.3345	0.2230	0.5153
DDX18	ATP-dependent RNA helicase DDX18	Q9NVP1	−0.2075	25.7948	1.3338	0.2232	0.5153
IPYR	Pyrophosphate phospho-hydrolase	Q15181	0.1779	29.4979	−1.3337	0.2233	0.5153
KLC1	Kinesin light chain 1	Q07866	−0.1583	25.2612	1.3326	0.2236	0.5153
CD027	UPF0609 protein C4orf27	Q9NWY4	0.2361	23.5708	−1.3527	0.2238	0.5153
DCTN4	Dynactin subunit 4	Q9UJW0	−0.2742	25.2725	1.3303	0.2243	0.5157

EIF2A	Eukaryotic translation initiation factor 2A	Q9BY44	−0.1616	25.9606	1.3243	0.2262	0.5194
ARF4	ADP-ribosylation factor 4	P18085	−0.2738	27.3286	1.3182	0.2282	0.5231
RL36A	60S ribosomal protein L36a	P83881	0.3862	28.1389	−1.3173	0.2284	0.5231
BIEA	Biliverdin reductase A	P53004	0.1464	25.6793	−1.3363	0.2288	0.5233
LYPA1	Lysophospholipase 1	O75608	−0.2325	26.0850	1.3142	0.2294	0.5237
AMPB	Aminopeptidase B	Q9H4A4	0.1661	24.3833	−1.3136	0.2296	0.5237
RL28	60S ribosomal protein L28	P46779	0.1935	28.1499	−1.3054	0.2322	0.5289
IF4H	Eukaryotic translation initiation factor 4H	Q15056	0.1880	28.3341	−1.3024	0.2332	0.5305
UBE2K	Ubiquitin-conjugating enzyme E2 K	P61086	0.1619	26.8316	−1.2996	0.2341	0.5318
NLTP	Sterol carrier protein 2	P22307	−0.1551	25.8039	1.2960	0.2353	0.5338
STK4	Serine/threonine-protein kinase 4	Q13043	0.3534	24.6365	−1.2933	0.2362	0.5351
PSD13	26S proteasome non-ATPase regulatory subunit 13	Q9UNM6	−0.1783	27.8471	1.2911	0.2369	0.5360
AATC	Glutamate oxaloacetate transaminase 1	P17174	−0.3335	27.1414	1.2876	0.2381	0.5373
OCAD2	Ovarian carcinoma immunoreactive antigen-like protein	Q56VL3	−0.3265	24.7500	1.2874	0.2381	0.5373
TSR1	Pre-rRNA-processing protein TSR1 homolog	Q2NL82	−0.1745	24.2187	1.2853	0.2388	0.5373
DHPR	Quinoid dihydropteridine reductase	P09417	0.1829	26.5281	−1.2826	0.2397	0.5373
ANXA7	Annexin A7	P20073	0.5197	25.2229	−1.2819	0.2399	0.5373
UBC9	Ubiquitin-conjugating enzyme E2 I	P63279	0.1448	26.3324	−1.2810	0.2402	0.5373
COMD9	COMM domain-containing protein 9	Q9P000	0.1857	24.8784	−1.2988	0.2407	0.5373
ODO2	2-oxoglutarate dehydrogenase complex component E2	P36957	−0.1535	25.1157	1.2794	0.2408	0.5373
SK2L2	Superkiller viralicidic activity 2-like 2	P42285	−0.1842	26.0546	1.2793	0.2408	0.5373
COPE	Coatomer subunit epsilon	O14579	0.2036	25.3585	−1.2790	0.2409	0.5373
PON2	Serum paraoxonase/arylesterase 2	Q15165	−0.1519	24.1901	1.2974	0.2411	0.5373
TM214	Transmembrane protein 214	Q6NUQ4	−0.2472	23.4471	1.2970	0.2412	0.5373
SMAP	Small acidic protein	O00193	0.2198	25.3675	−1.2754	0.2421	0.5376
MSH6	DNA mismatch repair protein Msh6	P52701	−0.1450	26.9003	1.2747	0.2423	0.5376
39326	Septin-7	Q16181	0.1429	26.8784	−1.2744	0.2424	0.5376
BYST	Bystin	Q13895	−0.1576	24.8060	1.2738	0.2426	0.5376
CT452	Cancer/testis antigen family 45 member A2	Q5DJT8	0.2356	24.8473	−1.2705	0.2437	0.5393
PRS10	Proteasome 26S subunit ATPase 6	P62333	0.1260	26.9847	−1.2669	0.2449	0.5413
UBP15	Ubiquitin-specific-processing protease 15	Q9Y4E8	−0.2305	25.7358	1.2595	0.2475	0.5451
GBB2	G protein subunit beta-2	P62879	0.4120	26.1795	−1.2585	0.2478	0.5451
AIFM1	Apoptosis-inducing factor 1, mitochondrial	O95831	0.1251	26.6041	−1.2584	0.2478	0.5451
MATR3	Matrin-3	P43243	0.1369	28.9662	−1.2582	0.2479	0.5451
NPM3	Nucleoplasmin-3	O75607	0.1408	24.7232	−1.2572	0.2483	0.5452
RRP12	RRP12-like protein	Q5JTH9	0.1291	25.7668	−1.2554	0.2489	0.5454
RL15	60S ribosomal protein L15	P61313	−0.1665	28.8994	1.2550	0.2490	0.5454
1433T	14-3-3 protein theta	P27348	0.1408	30.7848	−1.2473	0.2516	0.5505
ILF2	Nuclear factor of activated T-cells 45 kDa	Q12905	−0.1587	27.5501	1.2436	0.2529	0.5522

RS14	40S ribosomal protein S14	P62263	0.2279	29.1592	−1.2430	0.2531	0.5522
FEN1	Flap endonuclease 1	P39748	0.3199	27.3799	−1.2422	0.2534	0.5522
NOP2	Nucleolar protein 2 homolog	P46087	0.1274	27.7471	−1.2409	0.2539	0.5525
NRBP	Nuclear receptor-binding protein	Q9UHY1	−0.1851	26.7992	1.2357	0.2557	0.5547
MACF1	Microtubule-actin cross-linking factor 1, isoforms 1/2/3/5	Q9UPN3	−0.1305	28.4098	1.2355	0.2558	0.5547
RT35	28S ribosomal protein S35, mitochondrial	P82673	0.2026	23.5104	−1.2529	0.2558	0.5547
RS6	40S ribosomal protein S6	P62753	−0.1565	28.7760	1.2311	0.2573	0.5571
NEUL	Neurolysin, mitochondrial	Q9BYT8	0.1225	27.4092	−1.2291	0.2580	0.5575
LDHB	L-lactate dehydrogenase B chain	P07195	0.1770	30.4937	−1.2282	0.2583	0.5575
RANB3	Ran-binding protein 3	Q9H6Z4	0.2416	25.3210	−1.2278	0.2584	0.5575
LZIC	Leucine zipper and ICAT homologous domain-containing protein	Q8WZA0	0.2977	24.3450	−1.2427	0.2593	0.5587
STIP1	Stress-induced-phosphoprotein 1	P31948	−0.1313	31.0976	1.2236	0.2599	0.5590
EFTU	Elongation factor Tu, mitochondrial	P49411	0.1261	30.1755	−1.2231	0.2601	0.5590
PSMD9	26S proteasome non-ATPase regulatory subunit 9	O00233	0.2632	25.3686	−1.2378	0.2610	0.5603
SF3A3	Splicing factor 3A subunit 3	Q12874	0.1480	26.2682	−1.2173	0.2622	0.5620
ILF3	Interleukin enhancer-binding factor 3	Q12906	−0.1530	28.8596	1.2161	0.2626	0.5623
ELMO2	Engulfment and cell motility protein 2	Q96JJ3	−0.2216	23.4987	1.2322	0.2630	0.5623
PAIRB	Plasminogen activator inhibitor 1 RNA-binding protein	Q8NC51	0.1412	28.6640	−1.2122	0.2640	0.5639
PGRC2	Membrane-associated progesterone receptor component 2	O15173	−0.2195	26.1478	1.2106	0.2646	0.5644
NONO	Non-POU domain-containing octamer-binding protein	Q15233	0.1409	29.8041	−1.2067	0.2660	0.5660
PA2G4	Proliferation-associated protein 2G4	Q9UQ80	0.1146	30.4675	−1.2066	0.2660	0.5660
SAE1	SUMO-activating enzyme subunit 1	Q9UBE0	−0.1962	26.2852	1.2051	0.2666	0.5660
CSN1	G protein pathway suppressor 1	Q13098	−0.4068	24.5380	1.2049	0.2666	0.5660
COF2	Cofilin-2	Q9Y281	−0.1963	26.8230	1.2021	0.2677	0.5674
DEST	Destrin	P60981	0.1171	28.6877	−1.1998	0.2685	0.5685
MIF	Macrophage migration inhibitory factor	P14174	−0.3762	27.5676	1.1985	0.2690	0.5689
SYK	Lysine-tRNA ligase	Q15046	−0.1080	27.4650	1.1958	0.2700	0.5702
MON2	Protein MON2 homolog	Q7Z3U7	0.2264	23.7713	−1.1995	0.2746	0.5792
UTRO	Utrophin	P46939	0.2371	23.5166	−1.1963	0.2757	0.5809
EIF3H	Eukaryotic translation initiation factor 3 subunit H	O15372	0.1616	27.1740	−1.1773	0.2768	0.5823
CN37	2',3'-cyclic-nucleotide 3'-phosphodiesterase	P09543	−0.2395	25.4333	1.1761	0.2773	0.5823
BZW2	Basic leucine zipper and W2 domain-containing protein 2	Q9Y6E2	−0.2586	26.1945	1.1757	0.2774	0.5823
PHAX	Phosphorylated adapter RNA export protein	Q9H814	0.3086	24.1458	−1.1730	0.2785	0.5838
NDUA8	NADH dehydrogenase [ubiquinone] 1 alpha subcomplex subunit 8	P51970	−0.2017	23.6221	1.1861	0.2795	0.5844
RS27A	Ubiquitin-40S ribosomal protein S27a	P62979	0.1712	31.1443	−1.1697	0.2797	0.5844
G3P	Glyceraldehyde-3-phosphate dehydrogenase	P04406	0.1194	33.5314	−1.1692	0.2799	0.5844

MPCP	Solute carrier family 25 member 3	Q00325	−0.1161	30.0012	1.1686	0.2801	0.5844
BT3L4	Basic transcription factor 3-like 4	Q96K17	−0.1725	27.0724	1.1603	0.2832	0.5902
PARVA	Matrix-remodeling-associated protein 2	Q9NVD7	0.1773	24.9628	−1.1737	0.2840	0.5912
ACOT9	Acyl-CoA thioesterase 9	Q9Y305	−0.1565	25.7798	1.1557	0.2850	0.5923
FKBP2	Peptidyl-prolyl cis-trans isomerase FKBP2	P26885	0.4164	26.0223	−1.1550	0.2853	0.5923
SAHH	Adenosylhomocysteinase	P23526	0.1143	30.5377	−1.1526	0.2862	0.5926
MRE11	Meiotic recombination 11 homolog 1	P49959	−0.1143	24.6228	1.1525	0.2862	0.5926
ELAV1	Hu-antigen R	Q15717	−0.1548	26.2378	1.1503	0.2871	0.5926
PRDX6	Peroxiredoxin-6	P30041	0.1325	29.1934	−1.1502	0.2871	0.5926
IMDH2	Inosine-5'-monophosphate dehydrogenase 2	P12268	−0.1192	29.6605	1.1501	0.2872	0.5926
AP2B1	AP-2 complex subunit beta	P63010	0.1622	27.1497	−1.1492	0.2875	0.5926
MAGD2	Melanoma-associated antigen D2	Q9UNF1	0.2084	25.6152	−1.1467	0.2885	0.5932
RBM12	RNA-binding motif protein 12	Q9NTZ6	0.1514	26.0258	−1.1466	0.2885	0.5932
GSDMD	Gasdermin-D	P57764	0.1843	24.5741	−1.1609	0.2888	0.5932
TBB6	Tubulin beta-6 chain	Q9BUF5	0.1250	28.5935	−1.1450	0.2892	0.5932
SNW1	SNW domain-containing protein 1	Q13573	−0.2640	24.5907	1.1419	0.2903	0.5945
SSRA	Signal sequence receptor subunit alpha	P43307	−0.1179	26.0000	1.1416	0.2905	0.5945
SNX3	Sorting nexin-3	O60493	−0.2998	24.8969	1.1737	0.2920	0.5959
LUZP1	Leucine zipper protein 1	Q86V48	−0.1420	24.1427	1.1521	0.2922	0.5959
PLRG1	Pleiotropic regulator 1	O43660	−0.4204	24.9398	1.1727	0.2924	0.5959
NSDHL	Sterol-4-alpha-carboxylate 3-dehydrogenase, decarboxylating	Q15738	−0.3514	23.7891	1.1722	0.2926	0.5959
PLEC	Plectin	Q15149	−0.1122	31.3483	1.1344	0.2933	0.5961
AIMP1	Aminoacyl tRNA synthase complex-interacting multifunctional protein 1	Q12904	−0.2086	26.8522	1.1342	0.2934	0.5961
ATX10	Ataxin-10	Q9UBB4	0.1296	25.9382	−1.1328	0.2939	0.5965
LDHA	L-lactate dehydrogenase A chain	P00338	0.1227	31.9392	−1.1295	0.2952	0.5984
FAS	Fatty acid synthase	P49327	0.1131	30.9044	−1.1281	0.2957	0.5988
MARE1	Microtubule-associated protein RP/EB family member 1	Q15691	−0.1340	29.6466	1.1254	0.2968	0.6003
SYAC	Alanine-tRNA ligase, cytoplasmic	P49588	0.1116	29.0169	−1.1214	0.2984	0.6028
SC31A	Protein transport protein Sec31A	O94979	−0.2687	24.4013	1.1331	0.2995	0.6031
MK01	Mitogen-activated protein kinase 1	P28482	0.1544	25.8558	−1.1185	0.2996	0.6031
PSA4	Proteasome subunit alpha type-4	P25789	−0.1923	26.9921	1.1184	0.2996	0.6031
PSMD2	26S proteasome non-ATPase regulatory subunit 2	Q13200	0.1177	29.0011	−1.1151	0.3009	0.6050
AT1A1	Sodium pump subunit alpha-1	P05023	0.1351	28.4016	−1.1133	0.3017	0.6058
PSD12	26S proteasome non-ATPase regulatory subunit 12	O00232	0.1447	27.3738	−1.1115	0.3024	0.6066
FERM2	Fermitin family homolog 2	Q96AC1	0.1373	25.2749	−1.1088	0.3035	0.6080
TADBP	TAR DNA-binding protein 43	Q13148	0.1158	27.2827	−1.1067	0.3043	0.6089
ATPB	ATP synthase subunit beta, mitochondrial	P06576	−0.1015	30.8116	1.1059	0.3046	0.6089
CSN6	COP9 signalosome complex subunit 6	Q7L5N1	−0.2038	25.3038	1.1047	0.3051	0.6092
CXAR	Coxsackievirus and adenovirus receptor	P78310	−0.1465	23.9768	1.1035	0.3056	0.6094
RL13	60S ribosomal protein L13	P26373	−0.1153	29.7756	1.0986	0.3076	0.6125

TIM44	Mitochondrial import inner membrane translocase subunit TIM44	O43615	−0.3220	24.3127	1.1118	0.3079	0.6125
IF4B	Eukaryotic translation initiation factor 4B	P23588	0.1523	28.2809	−1.0955	0.3089	0.6138
RRBP1	Ribosome-binding protein 1	Q9P2E9	0.1073	28.4263	−1.0944	0.3093	0.6140
SRP72	Signal recognition particle 72 kDa protein	O76094	−0.1415	26.8573	1.0890	0.3116	0.6165
NUBP1	Nucleotide-binding protein 1	P53384	−0.4729	23.6308	1.1025	0.3116	0.6165
UBE2S	Ubiquitin-conjugating enzyme E2 S	Q16763	−0.2873	25.0125	1.0877	0.3121	0.6165
ANM1	Protein arginine N-methyltransferase 1	Q99873	−0.1105	28.5681	1.0877	0.3121	0.6165
RS13	40S ribosomal protein S13	P62277	0.1786	28.6326	−1.0870	0.3124	0.6165
BPNT1	Bisphosphate 3'-nucleotidase 1	O95861	−0.2373	24.1062	1.0996	0.3128	0.6165
AT5F1	ATPase subunit b	P24539	0.5969	25.7621	−1.0981	0.3134	0.6170
PAK2	p21-activated kinase 2	Q13177	−0.1505	26.5971	1.0834	0.3139	0.6173
LSM4	U6 snRNA-associated Sm-like protein LSm4	Q9Y4Z0	−0.1684	26.1862	1.0821	0.3144	0.6176
SNR40	U5 snRNP 40 kDa protein	Q96DI7	0.2405	26.0765	−1.0805	0.3151	0.6182
RAI3	G-protein coupled receptor family C group 5 member A	Q8NFJ5	0.3220	26.0123	−1.0788	0.3158	0.6189
IPO5	Importin-5	O00410	−0.1484	27.0836	1.0770	0.3165	0.6195
RIC8A	Synembryn-A	Q9NPQ8	0.1895	24.4644	−1.0764	0.3168	0.6195
PHF5A	PHD finger-like domain-containing protein 5A	Q7RTV0	−0.1885	24.1301	1.0859	0.3183	0.6208
GHC1	Solute carrier family 25 member 22	Q9H936	−0.1362	25.3342	1.0722	0.3185	0.6208
RS5	40S ribosomal protein S5	P46782	−0.2087	29.7489	1.0715	0.3188	0.6208
C1TC	C-1-tetrahydrofolate synthase, cytoplasmic	P11586	0.1080	29.5998	−1.0713	0.3189	0.6208
ARHL2	Poly(ADP-ribose) glycohydrolase ARH3	Q9NX46	−0.2184	24.6264	1.0827	0.3196	0.6215
NRDC	Nardilysin	O43847	−0.1686	25.4868	1.0672	0.3206	0.6221
SCAM3	Secretory carrier-associated membrane protein 3	O14828	0.2779	25.3702	−1.0671	0.3207	0.6221
BOP1	Block of proliferation 1 protein	Q14137	0.0975	25.7611	−1.0646	0.3217	0.6228
PRS7	Proteasome 26S subunit ATPase 2	P35998	−0.1045	28.3809	1.0645	0.3218	0.6228
MYH10	Myosin-10	P35580	0.1165	25.9203	−1.0619	0.3229	0.6240
CPSF5	Nudix motif 21	O43809	0.1885	25.6029	−1.0742	0.3231	0.6240
PAWR	PRKC apoptosis WT1 regulator protein	Q96IZ0	0.1804	24.8665	−1.0602	0.3236	0.6242
NSUN2	Substrate of AIM1/Aurora kinase B	Q08J23	−0.1456	25.7858	1.0563	0.3252	0.6258
DHX29	ATP-dependent RNA helicase DHX29	Q7Z478	−0.1338	24.9941	1.0563	0.3252	0.6258
CLH1	Clathrin heavy chain 1	Q00610	0.1083	31.4002	−1.0557	0.3255	0.6258
MYO1B	Unconventional myosin-Ib	O43795	−0.2409	24.6449	1.0530	0.3267	0.6258
THIK	Acetyl-CoA acyltransferase	P09110	0.2289	23.9966	−1.0527	0.3268	0.6258
PWP2	Periodic tryptophan protein 2 homolog	Q15269	0.3202	23.8951	−1.0526	0.3268	0.6258
ATPA	ATP synthase subunit alpha, mitochondrial	P25705	0.1040	30.3742	−1.0523	0.3270	0.6258
NUCL	Nucleolin	P19338	0.1142	30.1995	−1.0515	0.3273	0.6258
GPKOW	G patch domain and KOW motifs-containing protein	Q92917	0.1200	24.7655	−1.0479	0.3289	0.6280

ARPC5	Actin-related protein 2/3 complex subunit 5	O15511	0.1902	26.0451	−1.0463	0.3296	0.6284
SF3B2	Splicing factor 3B subunit 2	Q13435	0.1137	29.0564	−1.0457	0.3298	0.6284
SC23A	Protein transport protein Sec23A	Q15436	0.1955	25.1516	−1.0444	0.3304	0.6284
PELP1	Proline-, glutamic acid- and leucine-rich protein 1	Q8IZL8	0.1418	24.4827	−1.0436	0.3307	0.6284
EI2BB	Translation initiation factor eIF-2B subunit beta	P49770	0.2221	24.1763	−1.0735	0.3309	0.6284
RL18	60S ribosomal protein L18	Q07020	0.1938	29.7128	−1.0398	0.3323	0.6305
NQO1	NAD(P)H dehydrogenase	P15559	0.2455	27.0073	−1.0376	0.3333	0.6317
AN32E	Acidic leucine-rich nuclear phosphoprotein 32 family member E	Q9BTT0	−0.1997	24.7399	1.0359	0.3341	0.6324
AK1A1	Aldo-keto reductase family 1 member A1	P14550	−0.1874	26.0573	1.0350	0.3345	0.6324
GANAB	Neutral alpha-glucosidase AB	Q14697	0.2100	28.7010	−1.0332	0.3352	0.6327
MAP4	Microtubule-associated protein 4	P27816	−0.1037	30.0891	1.0319	0.3358	0.6327
ATPG	ATP synthase subunit gamma, mitochondrial	P36542	−0.1631	27.7233	1.0314	0.3360	0.6327
RAP1B	Ras-related protein Rap-1b	P61224	0.2404	25.8211	−1.0312	0.3361	0.6327
IF4A1	Eukaryotic initiation factor 4A-I	P60842	0.1359	30.2702	−1.0294	0.3369	0.6331
1433Z	14-3-3 protein zeta/delta	P63104	−0.1000	31.3834	1.0291	0.3370	0.6331
ARPC3	Actin-related protein 2/3 complex subunit 3	O15145	−0.3159	26.7927	1.0280	0.3375	0.6333
PP1A	Serine/threonine-protein phosphatase PP1-alpha catalytic subunit	P62136	0.2160	26.8828	−1.0259	0.3385	0.6338
SYMC	Methionine-tRNA ligase, cytoplasmic	P56192	−0.1149	27.9545	1.0257	0.3385	0.6338
CD123	Cell division cycle protein 123 homolog	O75794	0.2254	23.2512	−1.0510	0.3402	0.6355
CPNE3	Copine-3	O75131	0.4399	24.6351	−1.0339	0.3402	0.6355
BAZ1B	Bromodomain adjacent to zinc finger domain protein 1B	Q9UIG0	−0.1733	23.2797	1.0194	0.3413	0.6366
RFC4	Replication factor C subunit 4	P35249	−0.2301	24.3906	1.0478	0.3415	0.6366
RLA0	60S acidic ribosomal protein P0	P05388	−0.1271	29.6186	1.0174	0.3422	0.6366
EIF3K	Eukaryotic translation initiation factor 3 subunit K	Q9UBQ5	0.1699	26.9495	−1.0173	0.3422	0.6366
ITA5	Integrin alpha-5	P08648	−0.1288	24.2668	1.0159	0.3429	0.6366
NOP58	Nucleolar protein 58	Q9Y2X3	−0.1034	27.2701	1.0152	0.3432	0.6366
PABP4	Polyadenylate-binding protein 4	Q13310	0.1375	26.5548	−1.0145	0.3435	0.6366
IPO11	Importin-11	Q9UI26	0.3565	23.3070	−1.0116	0.3448	0.6366
RL35A	60S ribosomal protein L35a	P18077	0.1482	28.1750	−1.0113	0.3449	0.6366
RBP56	TATA-binding protein-associated factor 2N	Q92804	0.3971	25.0013	−1.0211	0.3457	0.6366
FAK1	Protein-tyrosine kinase 2	Q05397	0.1210	25.2519	−1.0094	0.3458	0.6366
RSMB	Small nuclear ribonucleoprotein-associated proteins B and B'	P14678	−0.1216	26.0459	1.0084	0.3462	0.6366
FTO	Fat mass and obesity-associated protein	Q9C0B1	0.1021	25.0679	−1.0081	0.3464	0.6366
PHLB1	Pleckstrin homology-like domain family B member 1	Q86UU1	−0.2097	24.1788	1.0196	0.3464	0.6366
CPT1A	Carnitine palmitoyltransferase 1A	P50416	0.0912	26.3152	−1.0076	0.3466	0.6366
1433F	14-3-3 protein eta (Protein AS1)	Q04917	0.1057	28.3465	−1.0063	0.3472	0.6366

GRHPR	Glyoxylate reductase/hydroxypyruvate reductase	Q9UBQ7	0.1183	25.4946	−1.0062	0.3472	0.6366
SMC4	Structural maintenance of chromosomes protein 4	Q9NTJ3	−0.2021	25.1469	1.0056	0.3475	0.6366
PSB1	Proteasome subunit beta type-1	P20618	−0.1799	26.4648	1.0033	0.3485	0.6379
SYSC	Seryl-tRNA synthetase	P49591	0.1614	27.1562	−1.0018	0.3492	0.6381
SYTC	Threonyl-tRNA synthetase	P26639	−0.1023	28.0454	1.0013	0.3494	0.6381
PWP1	Periodic tryptophan protein 1 homolog	Q13610	−0.3372	24.6470	1.0002	0.3499	0.6384
ADA	Adenosine deaminase	P00813	0.1333	26.9481	−0.9967	0.3515	0.6399
HNRPR	Heterogeneous nuclear ribonucleoprotein R	O43390	−0.1012	27.8639	0.9956	0.3520	0.6399
GRP78	Heat shock 70 kDa protein 5	P11021	0.1111	30.8800	−0.9955	0.3520	0.6399
CN142	Uncharacterized protein C14orf142	Q9BXV9	0.3369	23.2158	−0.9950	0.3522	0.6399
GUAA	GMP synthetase	P49915	−0.1137	26.8172	0.9934	0.3530	0.6401
WDR61	WD repeat-containing protein 61	Q9GZS3	0.1609	24.7174	−0.9932	0.3531	0.6401
TTC1	TPR repeat protein 1	Q99614	0.2429	25.3547	−0.9914	0.3539	0.6409
HDGF	Hepatoma-derived growth factor	P51858	0.1087	27.5403	−0.9894	0.3548	0.6413
RT27	28S ribosomal protein S27, mitochondrial	Q92552	0.2402	23.8838	−0.9893	0.3549	0.6413
HS90B	Heat shock protein HSP 90-beta	P08238	0.1128	31.9007	−0.9849	0.3569	0.6440
EI2BG	Translation initiation factor eIF-2B subunit gamma	Q9NR50	−0.1434	25.3613	0.9954	0.3571	0.6440
PROF1	Profilin-1	P07737	0.1360	30.3248	−0.9825	0.3580	0.6449
DBNL	Drebrin-like protein	Q9UJU6	−0.1102	26.2617	0.9788	0.3597	0.6471
VAPA	Vesicle-associated membrane protein-associated protein A	Q9P0L0	0.2879	26.9078	−0.9779	0.3601	0.6471
SF3B1	Splicing factor 3B subunit 1	O75533	−0.0956	27.3162	0.9773	0.3603	0.6471
HINT2	Histidine triad nucleotide-binding protein 2, mitochondrial	Q9BX68	−0.1784	23.6261	1.0017	0.3613	0.6472
DIAP1	Protein diaphanous homolog 1	O60610	0.1180	26.1796	−0.9748	0.3615	0.6472
TB182	182 kDa tankyrase-1-binding protein	Q9C0C2	0.1570	23.5090	−0.9850	0.3618	0.6472
PTN11	Tyrosine-protein phosphatase non-receptor type 11	Q06124	0.1386	24.6168	−0.9740	0.3619	0.6472
PPIL1	Peptidyl-prolyl cis-trans isomerase-like 1	Q9Y3C6	0.2688	26.9472	−0.9834	0.3626	0.6477
SPB1	pre-rRNA processing protein FTSJ3	Q8IY81	0.1152	26.5270	−0.9703	0.3636	0.6489
RAP1A	Ras-related protein Rap-1A	P62834	−0.1581	23.9684	0.9695	0.3640	0.6489
TIM13	Mitochondrial import inner membrane translocase subunit Tim13	Q9Y5L4	0.2882	25.5790	−0.9681	0.3646	0.6494
GORS2	Golgi reassembly-stacking protein 2	Q9H8Y8	−0.1184	24.4815	0.9774	0.3653	0.6499
RBM28	RNA-binding motif protein 28	Q9NW13	−0.1345	24.1468	0.9754	0.3662	0.6503
GLGB	Glycogen-branching enzyme	Q04446	−0.1254	24.4388	0.9751	0.3664	0.6503
QORX	Tumor protein p53-inducible protein 3	Q53FA7	0.1537	25.0849	−0.9634	0.3668	0.6503
WBP11	WW domain-binding protein 11	Q9Y2W2	−0.1462	25.3537	0.9621	0.3674	0.6503
TIM8A	Mitochondrial import inner membrane translocase subunit Tim8 A	O60220	−0.1873	25.3284	0.9621	0.3674	0.6503
2ABA	PP2A subunit B isoform PR55-alpha	P63151	−0.1257	26.0563	0.9567	0.3700	0.6541
RPAP3	RNA polymerase II-associated protein 3	Q9H6T3	0.0852	25.2592	−0.9537	0.3714	0.6554
SUV3	Suppressor of var1 3-like protein 1	Q8IYB8	−0.1213	22.9481	0.9634	0.3718	0.6554

UBP2L	Ubiquitin-associated protein 2-like	Q14157	−0.1061	26.8417	0.9523	0.3721	0.6554
ERF1	Eukaryotic peptide chain release factor subunit 1	P62495	0.1052	27.5244	−0.9512	0.3726	0.6554
TFG	TRK-fused gene protein	Q92734	−0.1422	26.2456	0.9511	0.3726	0.6554
FKBP4	Peptidyl-prolyl cis-trans isomerase FKBP4	Q02790	0.1329	28.8558	−0.9481	0.3741	0.6569
EIF3L	Eukaryotic translation initiation factor 3 subunit L	Q9Y262	0.0921	27.2734	−0.9477	0.3742	0.6569
COX41	Cytochrome c oxidase subunit 4 isoform 1, mitochondrial	P13073	0.1409	27.0239	−0.9448	0.3756	0.6587
EHD1	EH domain-containing protein 1	Q9H4M9	0.0873	26.8341	−0.9425	0.3767	0.6599
ACSL3	Long-chain acyl-CoA synthetase 3	O95573	−0.1387	25.5377	0.9417	0.3771	0.6600
FLNB	Filamin-B	O75369	−0.0921	31.4900	0.9405	0.3777	0.6603
NOL11	Nucleolar protein 11	Q9H8H0	−0.1001	24.1354	0.9395	0.3781	0.6604
CH10	10 kDa heat shock protein, mitochondrial	P61604	−0.1001	30.5364	0.9369	0.3794	0.6619
HMGA1	High mobility group protein A1	P17096	−0.1794	27.4311	0.9349	0.3804	0.6627
SFXN3	Sideroflexin-3	Q9BWM7	0.1813	25.7563	−0.9344	0.3806	0.6627
ANXA1	Annexin A1	P04083	0.1153	29.8076	−0.9335	0.3810	0.6628
MAP2	Methionine aminopeptidase 2	P50579	0.1060	26.9652	−0.9299	0.3828	0.6652
PDIA3	Protein disulfide-isomerase A3	P30101	0.0881	30.3016	−0.9261	0.3846	0.6672
RS28	40S ribosomal protein S28	P62857	0.1131	27.8773	−0.9259	0.3847	0.6672
TPM4	Tropomyosin alpha-4 chain	P67936	0.1366	27.3235	−0.9219	0.3867	0.6698
ADHX	Alcohol dehydrogenase 5	P11766	−0.1128	28.0185	0.9196	0.3878	0.6711
ALDOA	Fructose-bisphosphate aldolase A	P04075	0.1108	31.6476	−0.9188	0.3882	0.6711
KPRB	Phosphoribosyl pyrophosphate synthase-associated protein 2	O60256	0.0948	23.5202	−0.9155	0.3898	0.6732
NUP62	Nucleoporin Nup62	P37198	0.1511	24.2986	−0.9227	0.3910	0.6746
H2AY	Core histone macro-H2A.1	O75367	−0.0974	27.6057	0.9111	0.3920	0.6756
ITPA	Inosine triphosphatase	Q9BY32	−0.1164	26.4217	0.9086	0.3932	0.6770
VP26B	Vacuolar protein sorting-associated protein 26B	Q4G0F5	0.1333	24.6921	−0.9160	0.3942	0.6774
NGDN	Neuroguidin	Q8NEJ9	0.1932	24.8605	−0.9159	0.3943	0.6774
PPIH	Peptidyl-prolyl cis-trans isomerase H	O43447	0.1571	25.0655	−0.9153	0.3946	0.6774
TCPB	T-complex protein 1 subunit beta	P78371	0.0838	30.6869	−0.9034	0.3958	0.6787
PHB2	Prohibitin-2	Q99623	−0.1328	28.9791	0.9024	0.3963	0.6789
RAB2A	Ras-related protein Rab-2A	P61019	−0.0874	27.3426	0.9009	0.3970	0.6795
ADRO	Ferredoxin reductase	P22570	0.2703	22.6838	−0.9188	0.3993	0.6822
PPID	Peptidyl-prolyl cis-trans isomerase D	Q08752	0.1698	25.0604	−0.8954	0.3998	0.6822
MTDC	Bifunctional methylenetetrahydrofolate dehydrogenase/cyclohydrolase	P13995	0.2018	23.9977	−0.8953	0.3998	0.6822
NNRE	Apolipoprotein A-I-binding protein	Q8NCW5	0.1227	24.5770	−0.8940	0.4004	0.6825
CLPT1	Cleft lip and palate transmembrane protein 1	O96005	0.2038	25.6479	−0.8928	0.4011	0.6825
ACTN4	Alpha-actinin-4	O43707	0.0874	29.8967	−0.8926	0.4011	0.6825
RAD21	Double-strand-break repair protein rad21 homolog	O60216	−0.1223	23.3578	0.8909	0.4020	0.6833

GFPT1	Glutamine:fructose-6-phosphate amidotransferase 1	Q06210	−0.1294	27.2043	0.8899	0.4025	0.6833
AP2A1	Adaptor protein complex AP-2 subunit alpha-1	O95782	−0.1267	26.4473	0.8893	0.4028	0.6833
HNRPC	Heterogeneous nuclear ribonucleoproteins C1/C2	P07910	−0.1344	29.7182	0.8877	0.4036	0.6836
SARNP	SAP domain-containing ribonucleoprotein	P82979	0.1049	27.6102	−0.8869	0.4040	0.6836
DHX30	Putative ATP-dependent RNA helicase DHX30	Q7L2E3	−0.2441	24.3949	0.9066	0.4051	0.6836
H4	Histone H4	P62805	−0.1069	31.9679	0.8841	0.4054	0.6836
NMT1	Peptide N-myristoyltransferase 1	P30419	−0.3741	25.7063	0.8825	0.4063	0.6836
UBQL2	Ubiquilin-2	Q9UHD9	0.1771	24.8789	−0.8915	0.4063	0.6836
RBM14	RNA-binding protein 14	Q96PK6	0.0933	27.5280	−0.8820	0.4065	0.6836
LTOR5	Late endosomal/lysosomal adaptor and MAPK and MTOR activator 5	O43504	−0.1238	25.0385	0.8803	0.4074	0.6836
QCR2	Ubiquinol-cytochrome-c reductase complex core protein 2	P22695	−0.1010	27.1222	0.8796	0.4077	0.6836
SODC	Superoxide dismutase 1	P00441	−0.0952	29.5025	0.8791	0.4080	0.6836
RUVB2	RuvB-like 2	Q9Y230	0.1344	27.8824	−0.8791	0.4080	0.6836
CSN4	COP9 signalosome complex subunit 4	Q9BT78	0.1227	25.9121	−0.8788	0.4081	0.6836
RBM8A	RNA-binding motif protein 8A	Q9Y5S9	0.1585	25.9458	−0.8787	0.4081	0.6836
RLA1	60S acidic ribosomal protein P1	P05386	0.0952	29.6898	−0.8766	0.4093	0.6848
NAA10	N-alpha-acetyltransferase 10	P41227	−0.1466	26.4571	0.8736	0.4108	0.6855
RT29	Death-associated protein 3	P51398	0.3448	24.5768	−0.8822	0.4109	0.6855
NFU1	NFU1 iron-sulfur cluster scaffold homolog, mitochondrial	Q9UMS0	−0.1185	25.5514	0.8733	0.4109	0.6855
ADAS	Alkylglycerone-phosphate synthase	O00116	0.1730	25.9378	−0.8715	0.4118	0.6855
PSME1	Proteasome activator complex subunit 1	Q06323	−0.0824	26.4165	0.8714	0.4119	0.6855
CALX	Calnexin	P27824	−0.1084	30.4944	0.8709	0.4121	0.6855
PSMD6	26S proteasome non-ATPase regulatory subunit 6	Q15008	0.0954	27.7598	−0.8702	0.4125	0.6855
MCM2	Minichromosome maintenance protein 2 homolog	P49736	0.0779	28.4296	−0.8681	0.4136	0.6856
NUDC1	NudC domain-containing protein 1	Q96RS6	−0.1064	26.0985	0.8679	0.4137	0.6856
RFC2	Replication factor C subunit 2	P35250	−0.0769	25.8552	0.8677	0.4138	0.6856
RS10	40S ribosomal protein S10	P46783	−0.1859	24.3517	0.8728	0.4156	0.6880
UBXN1	UBX domain-containing protein 1	Q04323	0.2237	25.9050	−0.8624	0.4165	0.6888
STMN1	Stathmin	P16949	0.1869	27.5676	−0.8561	0.4198	0.6933
EIF3D	Eukaryotic translation initiation factor 3 subunit D	O15371	0.0806	27.3520	−0.8555	0.4201	0.6933
IMA3	Importin subunit alpha-3	O00629	−0.1839	24.8475	0.8539	0.4209	0.6940
HP1B3	Heterochromatin protein 1-binding protein 3	Q5SSJ5	−0.1134	26.0270	0.8514	0.4222	0.6951
CSK2B	Casein kinase II subunit beta	P67870	0.1538	27.2062	−0.8511	0.4223	0.6951
GLYC	Serine hydroxymethyltransferase, cytosolic	P34896	0.2153	25.4736	−0.8489	0.4235	0.6954
TCPA	T-complex protein 1 subunit alpha	P17987	−0.0790	30.2040	0.8485	0.4237	0.6954

VAPB	Vesicle-associated membrane protein-associated protein B/C	O95292	−0.2411	26.3338	0.8482	0.4239	0.6954
NCEH1	Neutral cholesterol ester hydrolase 1	Q6PIU2	−0.0927	26.0027	0.8469	0.4245	0.6954
UBP5	Ubiquitin-specific-processing protease 5	P45974	0.1106	27.3568	−0.8469	0.4245	0.6954
RM49	39S ribosomal protein L49, mitochondrial	Q13405	−0.1460	24.0480	0.8514	0.4265	0.6964
SYLC	Leucyl-tRNA synthetase	Q9P2J5	−0.0966	28.0242	0.8429	0.4266	0.6964
UPP1	Uridine phosphorylase 1	Q16831	−0.0904	26.0162	0.8429	0.4266	0.6964
LCAP	Leucyl-cystinyl aminopeptidase	Q9UIQ6	−0.0787	25.9641	0.8415	0.4274	0.6964
UAP1	UDP-N-acetylhexosamine pyrophosphorylase	Q16222	−0.0837	28.5667	0.8412	0.4275	0.6964
PRDX5	Peroxiredoxin-5, mitochondrial	P30044	−0.0904	28.1198	0.8411	0.4276	0.6964
RAB7A	Ras-related protein Rab-7a	P51149	−0.1056	28.2443	0.8389	0.4287	0.6972
BUB3	Mitotic checkpoint protein BUB3	O43684	−0.1030	27.5328	0.8382	0.4291	0.6972
PIN4	Peptidyl-prolyl cis-trans isomerase NIMA-interacting 4	Q9Y237	0.1435	25.0368	−0.8372	0.4296	0.6972
PHB	Prohibitin	P35232	0.0752	29.0149	−0.8370	0.4297	0.6972
DYN2	Dynamin-2	P50570	−0.1348	24.4922	0.8354	0.4305	0.6972
HSBP1	Heat shock factor-binding protein 1	O75506	−0.1505	26.3843	0.8348	0.4309	0.6972
DSG2	Desmoglein-2	Q14126	−0.1517	23.9607	0.8347	0.4309	0.6972
RL27A	60S ribosomal protein L27a	P46776	−0.1292	29.3801	0.8321	0.4323	0.6982
RBX1	RING-box protein 1	P62877	0.1960	25.0808	−0.8398	0.4325	0.6982
DDX47	Probable ATP-dependent RNA helicase DDX47	Q9H0S4	−0.1783	25.8595	0.8306	0.4331	0.6982
VP26A	Vesicle protein sorting 26A	O75436	0.1157	26.6569	−0.8294	0.4337	0.6982
GMPPA	GDP-mannose pyrophosphorylase A	Q96IJ6	−0.1253	23.2064	0.8285	0.4342	0.6982
PPM1G	Protein phosphatase 1G	O15355	−0.1160	27.6616	0.8283	0.4343	0.6982
ICAL	Calpastatin	P20810	0.0755	28.0999	−0.8279	0.4345	0.6982
RAE1L	mRNA export factor	P78406	−0.1385	24.8671	0.8273	0.4348	0.6982
HCD2	17-beta-hydroxysteroid dehydrogenase 10	Q99714	−0.1206	28.2700	0.8262	0.4355	0.6986
MBB1A	Myb-binding protein 1A	Q9BQG0	−0.0954	25.4765	0.8238	0.4367	0.6990
2A5D	PP2A B subunit isoform R5-delta	Q14738	−0.2309	24.9197	0.8309	0.4372	0.6990
BAG3	Bcl-2-associated athanogene 3	O95817	0.0908	26.2720	−0.8228	0.4373	0.6990
RPN2	Ribophorin-2	P04844	−0.1187	27.8159	0.8226	0.4374	0.6990
GRWD1	Glutamate-rich WD repeat-containing protein 1	Q9BQ67	0.1597	25.9050	−0.8215	0.4379	0.6990
SRPRB	Signal recognition particle receptor subunit beta	Q9Y5M8	0.1167	26.2016	−0.8207	0.4383	0.6990
TERA	Valosin-containing protein	P55072	0.0839	29.8644	−0.8182	0.4397	0.6990
RS21	40S ribosomal protein S21	P63220	0.1290	28.6169	−0.8181	0.4397	0.6990
RS20	40S ribosomal protein S20	P60866	0.1743	29.2291	−0.8180	0.4398	0.6990
IF6	Eukaryotic translation initiation factor 6	P56537	0.1327	28.9529	−0.8180	0.4398	0.6990
KBP	KIF1-binding protein	Q96EK5	0.0937	25.0812	−0.8145	0.4417	0.7013
PPIB	Peptidyl-prolyl cis-trans isomerase B	P23284	0.1041	29.8896	−0.8093	0.4445	0.7048
RPP30	Ribonuclease P protein subunit p30	P78346	−0.2481	23.5116	0.8276	0.4446	0.7048
ATP5J	ATP synthase-coupling factor 6, mitochondrial	P18859	0.2386	26.3567	−0.8074	0.4455	0.7055

VDAC2	Voltage-dependent anion-selective channel protein 2	P45880	0.0904	28.9597	−0.8051	0.4467	0.7065
DNJA3	DnaJ homolog subfamily A member 3, mitochondrial	Q96EY1	−0.1284	24.4310	0.8044	0.4471	0.7065
JAK1	Janus kinase 1	P23458	−0.1491	24.4145	0.8115	0.4474	0.7065
SNRPA	U1 small nuclear ribonucleoprotein A	P09012	−0.1254	26.9607	0.8013	0.4488	0.7081
FLII	Protein flightless-1 homolog	Q13045	0.0967	25.2903	−0.8004	0.4493	0.7082
BAX	Apoptosis regulator BAX	Q07812	0.1189	25.4765	−0.7992	0.4499	0.7086
TRXR1	Thioredoxin reductase 1, cytoplasmic	Q16881	0.1037	27.3816	−0.7978	0.4507	0.7091
PDC6I	Programmed cell death 6-interacting protein	Q8WUM4	0.0876	28.7408	−0.7938	0.4529	0.7115
ROA2	Heterogeneous nuclear ribonucleoproteins A2/B1	P22626	−0.0969	30.0334	0.7933	0.4531	0.7115
CPNE1	Copine-1	Q99829	0.1734	26.2893	−0.7927	0.4535	0.7115
FCL	Short-chain dehydrogenase/reductase family 4E member 1	Q13630	0.1148	24.6074	−0.7968	0.4553	0.7137
WDR43	WD repeat-containing protein 43	Q15061	0.2004	24.7505	−0.7867	0.4568	0.7154
TALDO	Transaldolase	P37837	0.0991	28.4662	−0.7847	0.4579	0.7165
RL21	60S ribosomal protein L21	P46778	0.1471	28.2826	−0.7779	0.4616	0.7203
LAMC1	Laminin subunit gamma-1	P11047	−0.0936	26.3805	0.7777	0.4617	0.7203
NDUS1	NADH-ubiquinone oxidoreductase 75 kDa subunit, mitochondrial	P28331	0.2515	24.2272	−0.7847	0.4618	0.7203
TCPQ	T-complex protein 1 subunit theta	P50990	−0.0725	30.7500	0.7772	0.4620	0.7203
ARF3	ADP-ribosylation factor 3	P61204	0.1197	27.4657	−0.7756	0.4629	0.7211
T22D2	TSC22 domain family protein 2	O75157	−0.1552	24.9500	0.7726	0.4646	0.7230
ABLM1	Actin-binding LIM protein 1	O14639	−0.1118	23.2313	0.7761	0.4665	0.7254
TCPG	T-complex protein 1 subunit gamma	P49368	−0.0873	30.2713	0.7608	0.4712	0.7317
KTN1	Kinectin	Q86UP2	−0.0935	26.9736	0.7601	0.4715	0.7317
GLRX3	Glutaredoxin-3	O76003	−0.0694	29.3185	0.7587	0.4723	0.7317
RAB8A	Ras-related protein Rab-8A	P61006	−0.2057	24.4621	0.7587	0.4723	0.7317
EIF3A	Eukaryotic translation initiation factor 3 subunit A	Q14152	0.0737	28.9862	−0.7518	0.4762	0.7367
HMGB2	High mobility group protein B2	P26583	0.1536	28.5114	−0.7515	0.4764	0.7367
SSA27	Sjoegren syndrome/scleroderma autoantigen 1	O60232	−0.1198	25.7315	0.7508	0.4768	0.7367
PIMT	L-isoaspartyl protein carboxyl methyltransferase	P22061	−0.1123	25.2511	0.7498	0.4774	0.7368
MK67I	Nucleolar protein interacting with the FHA domain of pKI-67	Q9BYG3	−0.1378	25.1678	0.7491	0.4778	0.7368
SYWC	Interferon-induced protein 53	P23381	0.2075	26.3415	−0.7469	0.4790	0.7381
MTA2	Metastasis-associated protein MTA2	O94776	−0.0856	24.9703	0.7452	0.4799	0.7386
PSA5	Proteasome subunit alpha type-5	P28066	−0.1335	28.1936	0.7448	0.4802	0.7386
RM11	39S ribosomal protein L11, mitochondrial	Q9Y3B7	−0.1883	24.0401	0.7437	0.4808	0.7389
TOPK	PDZ-binding kinase	Q96KB5	−0.0946	26.1377	0.7415	0.4821	0.7400
EPS15	Epidermal growth factor receptor substrate 15	P42566	0.1061	23.1262	−0.7404	0.4827	0.7400
PHOCN	MOB-like protein phocein	Q9Y3A3	−0.1243	24.1615	0.7467	0.4828	0.7400
STAM2	Signal transducing adapter molecule 2	O75886	−0.0917	23.8300	0.7333	0.4867	0.7452

EF2	Elongation factor 2	P13639	−0.0699	32.1131	0.7326	0.4871	0.7452
PSMD4	26S proteasome non-ATPase regulatory subunit 4	P55036	0.1278	27.8578	−0.7320	0.4875	0.7452
TCP4	Positive cofactor 4	P53999	0.1097	27.0859	−0.7305	0.4884	0.7455
CNN2	Calponin-2	Q99439	0.1833	26.6161	−0.7299	0.4887	0.7455
RPN1	Ribophorin 1	P04843	0.0921	28.0848	−0.7283	0.4896	0.7455
SRP54	Signal recognition particle 54 kDa protein	P61011	−0.1447	24.6943	0.7280	0.4898	0.7455
UBP47	Ubiquitin-specific-processing protease 47	Q96K76	−0.1032	23.2952	0.7279	0.4899	0.7455
ZYX	Zyxin	Q15942	−0.1153	27.2738	0.7243	0.4919	0.7479
USO1	General vesicular transport factor p115	O60763	−0.0995	26.0891	0.7203	0.4942	0.7507
SMD3	Small nuclear ribonucleoprotein Sm D3	P62318	0.1038	26.3710	−0.7194	0.4948	0.7509
LMNA	Prelamin-A/C	P02545	−0.0705	31.2302	0.7180	0.4956	0.7515
CD2AP	CD2-associated protein	Q9Y5K6	−0.1040	25.9681	0.7132	0.4983	0.7534
DC1I2	Cytoplasmic dynein 1 intermediate chain 2	Q13409	0.1138	26.6046	−0.7128	0.4986	0.7534
ELOB	Transcription elongation factor B polypeptide 2	Q15370	0.1644	28.0100	−0.7122	0.4990	0.7534
GSTM3	Glutathione S-transferase Mu 3	P21266	−0.1526	28.1037	0.7121	0.4990	0.7534
LIS1	Platelet-activating factor acetylhydrolase IB subunit alpha	P43034	0.0764	26.2746	−0.7113	0.4995	0.7534
DREB	Drebrin	Q16643	−0.0718	28.1373	0.7113	0.4995	0.7534
ARFG1	ADP-ribosylation factor GTPase-activating protein 1	Q8N6T3	0.1326	24.3533	−0.7245	0.5004	0.7538
RL23	60S ribosomal protein L23	P62829	−0.0852	29.4813	0.7093	0.5006	0.7538
HNRPL	Heterogeneous nuclear ribonucleoprotein L	P14866	0.0756	29.2854	−0.7062	0.5024	0.7544
RHOC	Rho-related GTP-binding protein RhoC	P08134	−0.2203	24.4057	0.7200	0.5029	0.7544
TOM6	Translocase of outer membrane 6 kDa subunit homolog	Q96B49	0.0816	25.0989	−0.7052	0.5030	0.7544
GCR	Nuclear receptor subfamily 3 group C member 1	P04150	−0.1307	24.6703	0.7041	0.5037	0.7544
ROA1	Heterogeneous nuclear ribonucleoprotein A1	P09651	0.1709	28.9308	−0.7028	0.5045	0.7544
WDR1	WD repeat-containing protein 1	O75083	0.0787	29.2404	−0.7028	0.5045	0.7544
REEP5	Receptor expression-enhancing protein 5	Q00765	−0.1773	25.9123	0.7027	0.5045	0.7544
DJB11	APOBEC1-binding protein 2	Q9UBS4	−0.1383	25.7761	0.7026	0.5046	0.7544
SRSF9	Serine/arginine-rich splicing factor 9	Q13242	−0.0774	25.5300	0.7010	0.5055	0.7548
PERQ2	GRB10-interacting GYF protein 2	Q6Y7W6	0.1565	24.0380	−0.7066	0.5057	0.7548
HSP7C	Heat shock 70 kDa protein 8	P11142	0.0671	32.3243	−0.6987	0.5068	0.7558
KIF2C	Kinesin-like protein KIF2C	Q99661	0.2119	24.5946	−0.6955	0.5088	0.7580
CNN3	Calponin-3	Q15417	−0.0843	28.0812	0.6944	0.5094	0.7580
RRP1	Ribosomal RNA processing protein 1 homolog A	P56182	0.0979	24.5711	−0.7083	0.5096	0.7580
40787	Septin-11	Q9NVA2	0.1923	26.3399	−0.6969	0.5114	0.7600
RHOA	Transforming protein RhoA	P61586	0.1463	24.9919	−0.6878	0.5133	0.7622
PML	Promyelocytic leukemia protein	P29590	0.2495	24.5755	−0.6923	0.5141	0.7624

NXF1	mRNA export factor TAP	Q9UBU9	0.0747	24.2979	−0.6858	0.5145	0.7624
ACINU	Apoptotic chromatin condensation inducer in the nucleus	Q9UKV3	−0.1295	24.5129	0.6908	0.5150	0.7624
RL30	60S ribosomal protein L30	P62888	−0.1576	28.4609	0.6846	0.5152	0.7624
PFD5	Myc modulator 1	Q99471	−0.0948	27.6929	0.6836	0.5158	0.7626
NP1L1	Nucleosome assembly protein 1-like 1	P55209	0.0754	29.8497	−0.6828	0.5163	0.7626
TIPRL	TIP41-like protein	O75663	0.0705	24.9974	−0.6807	0.5175	0.7626
HMCS1	HMG-CoA synthase	Q01581	−0.1297	24.3929	0.6849	0.5184	0.7626
AUP1	Ancient ubiquitous protein 1	Q9Y679	−0.1635	22.3507	0.6927	0.5186	0.7626
ADK	Adenosine kinase	P55263	0.1048	26.5524	−0.6785	0.5188	0.7626
TCRG1	Transcription elongation regulator 1	O14776	0.0952	24.5135	−0.6782	0.5190	0.7626
UGDH	UDP-Glc dehydrogenase	O60701	−0.0708	27.2764	0.6774	0.5195	0.7626
QCR7	Ubiquinol-cytochrome c reductase complex 14 kDa protein	P14927	0.1805	23.8942	−0.6772	0.5196	0.7626
TYSY	Thymidylate synthase	P04818	0.1365	26.5816	−0.6769	0.5198	0.7626
UCHL3	Ubiquitin carboxyl-terminal hydrolase isozyme L3	P15374	0.1012	24.9335	−0.6744	0.5213	0.7641
ERO1A	Endoplasmic reticulum oxidoreductase alpha	Q96HE7	−0.1294	24.8813	0.6723	0.5225	0.7653
LMNB1	Lamin-B1	P20700	0.0622	29.2924	−0.6702	0.5238	0.7661
PLIN3	Perilipin-3	O60664	−0.0768	29.0635	0.6699	0.5240	0.7661
COTL1	Coactosin-like protein	Q14019	−0.0985	29.1157	0.6672	0.5256	0.7678
PFD3	Von Hippel-Lindau-binding protein 1	P61758	0.0905	26.6962	−0.6665	0.5260	0.7678
AP1G1	Adaptor protein complex AP-1 subunit gamma-1	O43747	−0.1138	25.8333	0.6651	0.5269	0.7684
DX39B	Spliceosome RNA helicase DDX39B	Q13838	0.0708	27.0232	−0.6641	0.5275	0.7685
PSA1	Proteasome subunit alpha type-1	P25786	−0.1414	27.0235	0.6627	0.5283	0.7685
TEBP	Prostaglandin E synthase 3	Q15185	−0.1041	28.8749	0.6622	0.5287	0.7685
POP1	Ribonucleases P/MRP protein subunit POP1	Q99575	0.1188	25.0777	−0.6619	0.5288	0.7685
ERGI1	Endoplasmic reticulum-Golgi intermediate compartment protein 1	Q969X5	0.2107	25.1433	−0.6588	0.5307	0.7698
RAB9A	Ras-related protein Rab-9A	P51151	0.1716	24.8526	−0.6582	0.5310	0.7698
PFD6	Prefoldin subunit 6	O15212	−0.1365	26.0159	0.6559	0.5325	0.7698
SMRC1	SWI/SNF complex subunit SMARCC1	Q92922	0.1184	23.2286	−0.6557	0.5326	0.7698
GNL3L	Guanine nucleotide-binding protein-like 3-like protein	Q9NVN8	−0.1488	23.7938	0.6675	0.5332	0.7698
HYOU1	Hypoxia up-regulated protein 1	Q9Y4L1	−0.0686	28.5074	0.6541	0.5335	0.7698
HN1L	Hematological and neurological expressed 1-like protein	Q9H910	0.0671	27.3556	−0.6539	0.5337	0.7698
EMC2	ER membrane protein complex subunit 2	Q15006	0.0948	24.1323	−0.6583	0.5342	0.7698
OXSR1	Oxidative stress-responsive 1 protein	O95747	−0.1079	24.4004	0.6526	0.5345	0.7698
OSBP1	Oxysterol-binding protein 1	P22059	−0.1990	24.8781	0.6577	0.5346	0.7698
SCOT1	3-oxoacid CoA-transferase 1	P55809	−0.0952	23.8972	0.6577	0.5346	0.7698
TOLIP	Toll-interacting protein	Q9H0E2	−0.1701	23.2157	0.6631	0.5358	0.7705
HDAC2	Histone deacetylase 2	Q92769	−0.0852	24.6496	0.6501	0.5360	0.7705
SEC13	SEC13-like protein 1	P55735	0.1048	27.0212	−0.6487	0.5369	0.7711
GAGE1	G antigen 1	Q13065	−0.0601	28.1287	0.6439	0.5397	0.7746

THOC4	Aly/REF export factor	Q86V81	0.0785	26.5409	−0.6419	0.5410	0.7753
HNRPU	Heterogeneous nuclear ribonucleoprotein U	Q00839	0.0627	30.3084	−0.6415	0.5412	0.7753
RN213	RING finger protein 213	Q63HN8	0.1800	24.0942	−0.6461	0.5416	0.7753
RAN	GTPase Ran	P62826	−0.0603	29.8480	0.6373	0.5438	0.7779
AP3B1	Adaptor-related protein complex 3 subunit beta-1	O00203	0.0943	25.6016	−0.6353	0.5451	0.7779
KPYM	Pyruvate kinase PKM	P14618	−0.0594	31.8857	0.6350	0.5453	0.7779
NNMT	Nicotinamide N-methyltransferase	P40261	0.2412	25.5928	−0.6472	0.5453	0.7779
DNJA2	DnaJ homolog subfamily A member 2	O60884	−0.0888	27.0832	0.6344	0.5456	0.7779
PA1B3	Platelet-activating factor acetylhydrolase IB subunit gamma	Q15102	0.0767	26.7782	−0.6335	0.5462	0.7780
EMC1	ER membrane protein complex subunit 1	Q8N766	−0.0833	24.6268	0.6313	0.5475	0.7791
PAXI	Paxillin	P49023	0.1241	23.8649	−0.6357	0.5479	0.7791
RL1D1	Ribosomal L1 domain-containing protein 1	O76021	−0.0845	27.3947	0.6296	0.5486	0.7795
GBG12	Guanine nucleotide-binding protein G(I)/G(S)/G(O) subunit gamma-12	Q9UBI6	−0.1198	26.2778	0.6330	0.5496	0.7802
YBOX3	Y-box-binding protein 3	P16989	0.1095	26.8324	−0.6269	0.5502	0.7802
PRP19	Pre-mRNA-processing factor 19	Q9UMS4	0.1214	27.6564	−0.6265	0.5505	0.7802
CATB	Cathepsin B	P07858	0.1238	26.7459	−0.6253	0.5512	0.7807
SQRD	Sulfide:quinone oxidoreductase, mitochondrial	Q9Y6N5	−0.0681	28.3832	0.6217	0.5535	0.7832
PLXB2	Plexin-B2	O15031	0.1901	23.7466	−0.6196	0.5548	0.7841
HMGA2	High mobility group AT-hook protein 2	P52926	0.2031	27.6770	−0.6185	0.5555	0.7841
MYH9	Myosin-9	P35579	−0.0575	31.4714	0.6185	0.5555	0.7841
CSK21	Casein kinase II subunit alpha	P68400	0.2316	24.7556	−0.6144	0.5580	0.7870
UBP14	Ubiquitin-specific-processing protease 14	P54578	0.0575	27.7543	−0.6122	0.5594	0.7878
TBCE	Tubulin-specific chaperone E	Q15813	−0.1987	23.3113	0.6237	0.5595	0.7878
KAD6	Adenylate kinase isoenzyme 6	Q9Y3D8	0.1342	25.8907	−0.6104	0.5605	0.7881
DNJB1	DnaJ homolog subfamily B member 1	P25685	−0.1593	25.1971	0.6097	0.5609	0.7881
NAMPT	Nicotinamide phosphoribosyltransferase	P43490	0.1177	25.5538	−0.6095	0.5611	0.7881
KPRA	Phosphoribosyl pyrophosphate synthase-associated protein 1	Q14558	0.0761	26.3745	−0.6077	0.5622	0.7884
PRS4	Proteasome 26S subunit ATPase 1	P62191	−0.0610	28.3331	0.6075	0.5624	0.7884
NXP20	Nervous system overexpressed protein 20	Q8IWE2	0.1142	25.5280	−0.6069	0.5627	0.7884
PSA7	Proteasome subunit alpha type-7	O14818	−0.0905	27.5832	0.6050	0.5639	0.7894
ACON	Aconitase	Q99798	0.0912	25.7620	−0.6023	0.5656	0.7908
STOM	Stomatin	P27105	0.1076	25.3681	−0.6068	0.5657	0.7908
E41L2	Generally expressed protein 4.1	O43491	0.0719	26.8922	−0.5990	0.5677	0.7925
DLRB1	Dynein light chain roadblock-type 1	Q9NP97	−0.1486	26.4333	0.6033	0.5679	0.7925
RN114	RING finger protein 114	Q9Y508	0.1284	25.1322	−0.5979	0.5684	0.7925
IF2B	Eukaryotic translation initiation factor 2 subunit 2	P20042	−0.0663	27.5198	0.5959	0.5697	0.7937
CDK2	Cyclin-dependent kinase 2	P24941	0.1101	25.6620	−0.5916	0.5724	0.7966
HSP74	Heat shock 70 kDa protein 4	P34932	−0.0588	30.5812	0.5908	0.5729	0.7966

PRC2A	Proline-rich and coiled-coil-containing protein 2A	P48634	0.0802	24.2087	−0.5897	0.5736	0.7966
AF1L2	Actin filament-associated protein 1-like 2	Q8N4X5	0.0590	25.6506	−0.5896	0.5737	0.7966
DKC1	Dyskerin	O60832	−0.0767	25.5957	0.5885	0.5743	0.7969
SRP14	Signal recognition particle 14 kDa protein	P37108	−0.1570	27.3931	0.5861	0.5759	0.7984
ACSL1	Acyl-CoA synthetase 1	P33121	0.1196	23.7153	−0.5880	0.5775	0.7984
FUBP3	Far upstream element-binding protein 3	Q96I24	−0.0645	24.7506	0.5831	0.5778	0.7984
RENT1	Up-frameshift suppressor 1 homolog	Q92900	0.0604	27.0204	−0.5825	0.5782	0.7984
RPAB3	DNA-directed RNA polymerase II subunit H	P52434	0.1187	24.2636	−0.5821	0.5784	0.7984
ATOX1	Copper transport protein ATOX1	O00244	0.1688	25.5408	−0.5860	0.5787	0.7984
HNRPQ	Synaptotagmin-binding, cytoplasmic RNA-interacting protein	O60506	−0.0561	28.7907	0.5811	0.5790	0.7984
ASNA	Arsenite-stimulated ATPase	O43681	−0.1225	26.0358	0.5810	0.5791	0.7984
AAR2	AAR2 splicing factor homolog	Q9Y312	0.2577	23.7059	−0.5769	0.5817	0.8013
PUR8	Adenylosuccinate lyase	P30566	0.1082	26.7952	−0.5763	0.5821	0.8013
PSB5	Proteasome subunit beta type-5	P28074	−0.0615	27.1890	0.5730	0.5842	0.8029
CPIN1	Cytokine-induced apoptosis inhibitor 1	Q6FI81	−0.0672	26.8902	0.5730	0.5842	0.8029
IF4A3	Eukaryotic initiation factor 4A-III	P38919	0.0661	29.0705	−0.5707	0.5857	0.8040
MYG1	UPF0160 protein MYG1, mitochondrial	Q9HB07	−0.0591	26.0905	0.5702	0.5860	0.8040
DAD1	Defender against cell death 1	P61803	−0.0876	25.8102	0.5695	0.5865	0.8040
ADDA	Alpha-adducin	P35611	0.1049	24.1027	−0.5702	0.5888	0.8065
GELS	Gelsolin	P06396	0.0793	25.5427	−0.5607	0.5921	0.8105
ACL6A	Actin-like protein 6A	O96019	0.0714	25.6172	−0.5590	0.5933	0.8114
ENPL	Heat shock protein 90 kDa beta member 1	P14625	−0.0620	31.0484	0.5580	0.5939	0.8116
RALY	hnRNP associated with lethal yellow protein homolog	Q9UKM9	0.0640	27.3070	−0.5563	0.5950	0.8124
SNX2	Sorting nexin-2	O60749	−0.0852	27.6610	0.5539	0.5965	0.8135
PFD1	Prefoldin subunit 1	O60925	0.0536	27.3281	−0.5537	0.5967	0.8135
BCLF1	Bcl-2-associated transcription factor 1	Q9NYF8	−0.0553	25.9224	0.5524	0.5975	0.8140
SSRP1	Structure-specific recognition protein 1	Q08945	0.0551	26.9726	−0.5509	0.5985	0.8146
PSMD7	26S proteasome non-ATPase regulatory subunit 7	P51665	0.1252	25.5204	−0.5503	0.5989	0.8146
COF1	Cofilin-1	P23528	0.0590	31.2665	−0.5448	0.6025	0.8181
REQU	D4, zinc and double PHD fingers family 2	Q92785	−0.0825	23.0115	0.5545	0.6025	0.8181
PPWD1	Peptidylprolyl isomerase domain and WD repeat-containing protein 1	Q96BP3	0.0957	23.9273	−0.5478	0.6032	0.8184
S29A1	Solute carrier family 29 member 1	Q99808	0.1965	26.7445	−0.5459	0.6044	0.8192
CAP2	Adenylyl cyclase-associated protein 2	P40123	0.0651	25.2772	−0.5414	0.6047	0.8192
CBX3	Chromobox protein homolog 3	Q13185	0.0602	27.4376	−0.5400	0.6056	0.8197
SON	Negative regulatory element-binding protein	P18583	−0.0621	24.8046	0.5366	0.6079	0.8212
HN1	Hematological and neurological expressed 1 protein	Q9UK76	−0.0803	28.5573	0.5358	0.6084	0.8212
PGM1	Phosphoglucomutase-1	P36871	0.0866	26.2668	−0.5357	0.6084	0.8212

TIM9	Mitochondrial import inner membrane translocase subunit Tim9	Q9Y5J7	−0.0834	24.0675	0.5443	0.6090	0.8212
GBLP	Guanine nucleotide-binding protein subunit beta-2-like 1	P63244	0.0625	30.2573	−0.5347	0.6091	0.8212
SMC1A	Structural maintenance of chromosomes protein 1A	Q14683	−0.0905	25.5143	0.5336	0.6098	0.8216
CHRD1	Cysteine and histidine-rich domain-containing protein 1	Q9UHD1	0.0838	27.6075	−0.5324	0.6106	0.8220
ELOC	Transcription elongation factor B polypeptide 1	Q15369	0.1330	28.0558	−0.5307	0.6118	0.8229
NTF2	Nuclear transport factor 2	P61970	0.0789	27.0072	−0.5298	0.6123	0.8230
AP2A2	Adaptor protein complex AP-2 subunit alpha-2	O94973	−0.1237	23.4921	0.5286	0.6131	0.8234
NDUS3	NADH dehydrogenase [ubiquinone] iron-sulfur protein 3, mitochondrial	O75489	−0.1021	23.5562	0.5365	0.6140	0.8240
RL10A	60S ribosomal protein L10a	P62906	−0.0535	29.5905	0.5230	0.6168	0.8268
MEA1	Male-enhanced antigen 1	Q16626	0.0848	23.5526	−0.5263	0.6171	0.8268
OLA1	Obg-like ATPase 1	Q9NTK5	−0.0879	27.2094	0.5179	0.6202	0.8297
SPCS3	Signal peptidase complex subunit 3	P61009	−0.0864	25.7037	0.5216	0.6202	0.8297
TNPO1	Importin beta-2	Q92973	0.0800	27.9120	−0.5165	0.6211	0.8300
SPG20	Spastic paraplegia 20 protein	Q8N0X7	−0.2112	24.1452	0.5251	0.6214	0.8300
UCHL1	Ubiquitin carboxyl-terminal hydrolase isozyme L1	P09936	0.0663	31.4467	−0.5097	0.6256	0.8348
LRWD1	Leucine-rich repeat and WD repeat-containing protein 1	Q9UFC0	0.0918	22.5848	−0.5129	0.6260	0.8348
DAG1	Dystrophin-associated glycoprotein 1	Q14118	0.1093	24.1246	−0.5066	0.6277	0.8359
IF2B1	Insulin-like growth factor 2 mRNA-binding protein 1	Q9NZI8	0.0496	28.4491	−0.5065	0.6278	0.8359
PAIP1	Poly(A)-binding protein-interacting protein 1	Q9H074	−0.0779	23.9145	0.5024	0.6305	0.8389
STML2	Stomatin-like protein 2, mitochondrial	Q9UJZ1	0.0691	26.4160	−0.5006	0.6317	0.8398
ADT2	Solute carrier family 25 member 5	P05141	0.1013	27.6894	−0.4983	0.6333	0.8403
EIF3F	Eukaryotic translation initiation factor 3 subunit F	O00303	0.0631	27.9407	−0.4976	0.6337	0.8403
TPM1	Tropomyosin-1	P09493	−0.1750	27.0061	0.5007	0.6340	0.8403
NU214	Nucleoporin Nup214	P35658	0.1091	24.6053	−0.5056	0.6341	0.8403
PRI1	DNA primase small subunit	P49642	−0.0794	24.5716	0.4999	0.6345	0.8403
IF2G	Eukaryotic translation initiation factor 2 subunit 3	P41091	−0.0568	29.4525	0.4923	0.6373	0.8427
PDLI1	PDZ and LIM domain protein 1	O00151	0.0578	27.7201	−0.4919	0.6376	0.8427
E2AK2	Eukaryotic translation initiation factor 2-alpha kinase 2	P19525	−0.0510	24.5102	0.4915	0.6379	0.8427
BAG6	Bcl2-associated athanogene 6	P46379	−0.0846	25.5499	0.4908	0.6383	0.8427
BCAT1	Branched-chain-amino-acid aminotransferase, cytosolic	P54687	0.2127	23.7057	−0.4960	0.6404	0.8448
AP2M1	Adaptor protein complex AP-2 subunit mu	Q96CW1	−0.0463	26.5565	0.4852	0.6420	0.8464
BTF3	RNA polymerase B transcription factor 3	P20290	0.0643	26.8220	−0.4833	0.6434	0.8466
NCPR	NADPH--cytochrome P450 reductase	P16435	−0.0813	24.4638	0.4852	0.6444	0.8466

TGM2	Transglutaminase-2	P21980	−0.0745	25.2052	0.4817	0.6444	0.8466
PLSI	Plastin-1	Q14651	−0.0804	25.5671	0.4810	0.6449	0.8466
U520	U5 snRNP-specific 200 kDa protein	O75643	−0.0530	26.9373	0.4810	0.6449	0.8466
DHX9	ATP-dependent RNA helicase A (RHA)	Q08211	−0.0568	29.1078	0.4805	0.6452	0.8466
FACE1	Zinc metalloproteinase Ste24 homolog	O75844	0.1002	23.8683	−0.4816	0.6468	0.8480
OGFR	Opioid growth factor receptor	Q9NZT2	0.1030	24.4144	−0.4758	0.6484	0.8496
NDRG3	N-myc downstream-regulated gene 3 protein	Q9UGV2	0.0812	25.5733	−0.4732	0.6502	0.8510
FLNA	Filamin-A	P21333	0.0568	31.9566	−0.4721	0.6509	0.8510
APMAP	Adipocyte plasma membrane-associated protein	Q9HDC9	−0.0528	27.7254	0.4719	0.6511	0.8510
EIF3M	Eukaryotic translation initiation factor 3 subunit M	Q7L2H7	0.1562	26.0031	−0.4716	0.6535	0.8530
SFXN1	Sideroflexin-1	Q9H9B4	0.0511	27.1121	−0.4682	0.6536	0.8530
RL7	60S ribosomal protein L7	P18124	0.0514	30.1079	−0.4676	0.6540	0.8530
RS15	40S ribosomal protein S15	P62841	−0.0931	27.3476	0.4657	0.6553	0.8540
TRMB	Methyltransferase-like protein 1	Q9UBP6	0.0736	24.4541	−0.4647	0.6560	0.8542
LAP2	Erbb2-interacting protein	Q96RT1	−0.0695	26.2848	0.4610	0.6585	0.8555
PSB7	Proteasome subunit beta type-7	Q99436	0.1965	25.8616	−0.4596	0.6594	0.8555
SPS1	Selenophosphate synthase 1	P49903	−0.0789	23.9303	0.4627	0.6595	0.8555
RAB14	Ras-related protein Rab-14	P61106	−0.0484	25.7811	0.4593	0.6597	0.8555
SND1	Staphylococcal nuclease domain-containing protein 1	Q7KZF4	0.0485	27.8224	−0.4590	0.6599	0.8555
STAT3	Signal transducer and activator of transcription 3	P40763	−0.0461	26.0437	0.4589	0.6599	0.8555
SYEP	Bifunctional aminoacyl-tRNA synthetase	P07814	−0.0442	29.4361	0.4571	0.6612	0.8560
UBP10	Ubiquitin-specific-processing protease 10	Q14694	0.0518	24.0314	−0.4566	0.6616	0.8560
HS90A	Heat shock protein HSP 90-alpha	P07900	0.0472	30.3626	−0.4561	0.6618	0.8560
CAPR1	Cytoplasmic activation- and proliferation-associated protein 1	Q14444	0.0503	28.9042	−0.4519	0.6647	0.8577
CBPA4	Carboxypeptidase A4	Q9UI42	−0.0886	26.3050	0.4515	0.6650	0.8577
C43BP	Collagen type IV alpha-3-binding protein	Q9Y5P4	0.0824	24.3795	−0.4504	0.6658	0.8577
STXB3	Syntaxin-binding protein 3	O00186	0.0549	23.5878	−0.4516	0.6671	0.8577
STRAP	Serine-threonine kinase receptor-associated protein	Q9Y3F4	−0.0416	30.4594	0.4464	0.6685	0.8577
AAKG1	5'-AMP-activated protein kinase subunit gamma-1	P54619	0.1018	23.9879	−0.4488	0.6690	0.8577
PSMF1	Proteasome inhibitor PI31 subunit	Q92530	0.1538	26.3950	−0.4458	0.6690	0.8577
ACPM	NADH-ubiquinone oxidoreductase 9.6 kDa subunit	O14561	0.1576	23.2129	−0.4454	0.6692	0.8577
AT2A2	Sarcoplasmic/endoplasmic reticulum calcium ATPase 2	P16615	−0.0453	28.7137	0.4438	0.6703	0.8577
MP2K3	MAPK/ERK kinase 3	P46734	0.0562	26.2771	−0.4435	0.6705	0.8577
GNAS1	Guanine nucleotide-binding protein G(s) subunit alpha isoforms XLas	Q5JWF2	−0.0645	25.3233	0.4421	0.6715	0.8577

ANXA2	Annexin A2	P07355	0.1365	31.2755	−0.4417	0.6718	0.8577
ANXA5	Annexin A5	P08758	0.0411	28.9187	−0.4415	0.6719	0.8577
TCPZ	T-complex protein 1 subunit zeta	P40227	−0.0504	29.9937	0.4414	0.6720	0.8577
UBA1	Ubiquitin-like modifier-activating enzyme 1	P22314	−0.0408	29.8651	0.4413	0.6721	0.8577
SYYC	Tyrosyl-tRNA synthetase	P54577	0.0512	28.4216	−0.4411	0.6722	0.8577
UCHL5	Ubiquitin carboxyl-terminal hydrolase isozyme L5	Q9Y5K5	0.0896	25.4854	−0.4406	0.6725	0.8577
BRE1B	RING finger protein 40	O75150	−0.0745	23.8201	0.4435	0.6726	0.8577
CUL4B	Cullin-4B (CUL-4B)	Q13620	−0.0539	25.3626	0.4402	0.6728	0.8577
IF2P	Eukaryotic translation initiation factor 5B	O60841	−0.0721	26.9909	0.4394	0.6734	0.8577
MARCS	Myristoylated alanine-rich C-kinase substrate	P29966	0.0823	28.9929	−0.4374	0.6747	0.8577
UCRI	Ubiquinol-cytochrome c reductase iron-sulfur subunit	P47985	−0.0943	24.3339	0.4365	0.6754	0.8577
PYR1	CAD protein	P27708	−0.0474	28.7375	0.4362	0.6756	0.8577
QCR1	Ubiquinol-cytochrome-c reductase complex core protein 1	P31930	0.0619	27.6788	−0.4356	0.6760	0.8577
NB5R3	NADH-cytochrome b5 reductase 3	P00387	−0.0438	27.0139	0.4353	0.6762	0.8577
TYB4	Thymosin beta-4	P62328	0.2179	30.1207	−0.4412	0.6770	0.8577
PPIF	Peptidyl-prolyl cis-trans isomerase F, mitochondrial	P30405	0.0676	25.7178	−0.4410	0.6772	0.8577
VATB2	V-type proton ATPase subunit B, brain isoform	P21281	0.0803	25.2554	−0.4328	0.6780	0.8577
RUXF	Small nuclear ribonucleoprotein F	P62306	0.0542	27.0752	−0.4319	0.6785	0.8577
CDC5L	Cell division cycle 5-like protein	Q99459	−0.0713	26.2531	0.4318	0.6786	0.8577
PLST	Plastin-3	P13797	0.0428	28.0522	−0.4318	0.6786	0.8577
ETFA	Electron transfer flavoprotein subunit alpha, mitochondrial	P13804	−0.0566	27.7854	0.4285	0.6809	0.8595
ABCF1	ATP-binding cassette sub-family F member 1	Q8NE71	−0.0463	27.4269	0.4283	0.6811	0.8595
MYO1C	Unconventional myosin-Ic	O00159	0.0448	27.7072	−0.4266	0.6822	0.8601
DNMT1	DNA (cytosine-5)-methyltransferase 1	P26358	−0.0437	25.7010	0.4261	0.6826	0.8601
S35F2	Solute carrier family 35 member F2	Q8IXU6	0.0795	24.4193	−0.4272	0.6838	0.8604
MA7D1	MAP7 domain-containing protein 1	Q3KQU3	−0.0567	25.0443	0.4244	0.6838	0.8604
TCPH	T-complex protein 1 subunit eta	Q99832	−0.0420	29.8546	0.4235	0.6844	0.8605
S10AA	S100 calcium-binding protein A10	P60903	0.2883	25.4642	−0.4252	0.6852	0.8606
KIF2A	Kinesin-like protein KIF2A	O00139	−0.1044	24.3788	0.4204	0.6866	0.8606
COR1C	Coronin-1C	Q9ULV4	0.0451	28.5327	−0.4203	0.6866	0.8606
RS8	40S ribosomal protein S8	P62241	−0.0425	30.4868	0.4202	0.6867	0.8606
S10A6	S100 calcium-binding protein A6	P06703	−0.0672	30.1760	0.4227	0.6869	0.8606
ARP3	Actin-related protein 3	P61158	−0.0469	27.9036	0.4186	0.6878	0.8610
EIF3E	Eukaryotic translation initiation factor 3 subunit E	P60228	−0.0554	27.7512	0.4173	0.6887	0.8615
SYNC	Asparaginyl-tRNA synthetase	O43776	−0.0478	27.8966	0.4142	0.6909	0.8624
ACADM	Medium-chain specific acyl-CoA dehydrogenase, mitochondrial	P11310	−0.0611	24.9965	0.4142	0.6909	0.8624
RINI	Ribonuclease inhibitor	P13489	0.0411	29.2989	−0.4135	0.6914	0.8624

PSA3	Proteasome subunit alpha type-3	P25788	0.1352	25.3405	−0.4162	0.6914	0.8624
SRSF7	Serine/arginine-rich splicing factor 7	Q16629	0.0668	26.4035	−0.4125	0.6921	0.8626
THIM	Acetyl-CoA acyltransferase	P42765	−0.0983	24.4894	0.4111	0.6931	0.8632
TR150	Thyroid hormone receptor-associated protein 3	Q9Y2W1	0.0775	26.1686	−0.4065	0.6963	0.8661
RTCB	tRNA-splicing ligase RtcB homolog	Q9Y3I0	−0.0638	28.1986	0.4063	0.6964	0.8661
RFA1	Replication factor A protein 1	P27694	0.0621	26.9170	−0.4044	0.6978	0.8671
SDHA	Succinate dehydrogenase [ubiquinone] flavoprotein subunit, mitochondrial	P31040	−0.0775	26.0722	0.4034	0.6984	0.8674
LIMA1	LIM domain and actin-binding protein 1	Q9UHB6	−0.0414	25.1435	0.4024	0.6992	0.8676
RAI14	Retinoic acid-induced protein 14	Q9P0K7	−0.0753	23.6506	0.4017	0.6997	0.8676
TRI25	Tripartite motif-containing protein 25	Q14258	−0.0970	26.9416	0.3999	0.7009	0.8683
TWF1	Twinfilin-1	Q12792	0.0501	27.5500	−0.3995	0.7012	0.8683
PPM1B	Protein phosphatase 1B	O75688	0.1065	23.6135	−0.3971	0.7047	0.8707
HEXB	Beta-hexosaminidase subunit beta	P07686	0.0502	25.4905	−0.3938	0.7052	0.8707
LMCD1	LIM and cysteine-rich domains protein 1	Q9NZU5	−0.0815	24.2968	0.3961	0.7054	0.8707
H2A1B	Histone H2A.2	P04908	0.1519	30.3626	−0.3948	0.7063	0.8707
STAT1	Signal transducer and activator of transcription 1-alpha/beta	P42224	0.0416	27.1331	−0.3921	0.7064	0.8707
CCHL	Holocytochrome c-type synthase	P53701	0.0456	24.5540	−0.3917	0.7067	0.8707
IF4G1	Eukaryotic translation initiation factor 4 gamma 1	Q04637	−0.0395	27.9320	0.3905	0.7076	0.8707
F120A	Oxidative stress-associated Src activator	Q9NZB2	−0.0821	24.0146	0.3901	0.7079	0.8707
FAF2	FAS-associated factor 2	Q96CS3	0.0481	26.1348	−0.3901	0.7079	0.8707
RAB5C	Ras-related protein Rab-5C	P51148	0.0848	26.0612	−0.3896	0.7082	0.8707
AMPL	Leucine aminopeptidase 3	P28838	0.0631	28.0357	−0.3876	0.7096	0.8718
RS15A	40S ribosomal protein S15a	P62244	−0.0414	29.2935	0.3847	0.7117	0.8737
KDEL2	KDEL motif-containing protein 2	Q7Z4H8	−0.0448	24.9629	0.3835	0.7125	0.8741
ZCCHL	Zinc finger CCCH-type antiviral protein 1-like	Q96H79	−0.0571	25.5111	0.3850	0.7132	0.8743
RSU1	Ras suppressor protein 1	Q15404	0.0525	26.4107	−0.3808	0.7144	0.8751
IMA1	Importin subunit alpha-1	P52292	−0.0446	29.3140	0.3769	0.7172	0.8767
ORN	RNA exonuclease 2 homolog	Q9Y3B8	0.0509	26.3022	−0.3764	0.7175	0.8767
RB6I2	ELKS/Rab6-interacting/CAST family member 1	Q8IUD2	0.1148	24.2400	−0.3758	0.7180	0.8767
FUMH	Fumarate hydratase, mitochondrial	P07954	−0.0943	25.8298	0.3755	0.7182	0.8767
PCNA	Proliferating cell nuclear antigen	P12004	0.0432	30.0512	−0.3753	0.7183	0.8767
6PGL	6-phosphogluconolactonase	O95336	0.0573	26.2819	−0.3742	0.7191	0.8767
RUVB1	RuvB-like 1	Q9Y265	−0.0393	28.2939	0.3735	0.7196	0.8767
COA3	Cytochrome c oxidase assembly factor 3 homolog, mitochondrial	Q9Y2R0	0.0716	25.2512	−0.3752	0.7201	0.8767
MIC60	Mitochondrial inner membrane protein	Q16891	−0.0538	27.1343	0.3725	0.7203	0.8767
ECH1	Delta(3,5)-Delta(2,4)-dienoyl-CoA isomerase, mitochondrial	Q13011	0.0401	26.0098	−0.3709	0.7215	0.8770
SKP1	S-phase kinase-associated protein 1	P63208	−0.0771	27.1708	0.3708	0.7216	0.8770
TM165	Transmembrane protein 165	Q9HC07	0.1011	24.1966	−0.3685	0.7232	0.8774

ACPH	Acylamino-acid-releasing enzyme	P13798	−0.0816	25.4802	0.3680	0.7235	0.8774
PGAM1	Phosphoglycerate mutase 1	P18669	−0.0400	30.5852	0.3678	0.7237	0.8774
ECHB	Trifunctional enzyme subunit beta, mitochondrial (TP-beta)	P55084	−0.0395	27.5166	0.3675	0.7239	0.8774
P20D2	Peptidase M20 domain-containing protein 2	Q8IYS1	0.1596	23.8834	−0.3721	0.7247	0.8777
CYFP1	Cytoplasmic FMR1-interacting protein 1	Q7L576	−0.0472	24.9633	0.3636	0.7267	0.8787
BRX1	Ribosome biogenesis protein BRX1 homolog	Q8TDN6	−0.1279	24.0329	0.3633	0.7269	0.8787
TFR1	Transferrin receptor protein 1	P02786	0.0376	28.4821	−0.3620	0.7278	0.8787
BCCIP	BRCA2 and CDKN1A-interacting protein	Q9P287	0.0596	26.2282	−0.3615	0.7282	0.8787
ACADV	Very long-chain specific acyl-CoA dehydrogenase, mitochondrial	P49748	0.0365	25.4690	−0.3606	0.7288	0.8787
ATPK	ATP synthase subunit f, mitochondrial	P56134	0.1779	22.6857	−0.3600	0.7292	0.8787
SUCB1	Succinyl-CoA synthetase beta-A chain	Q9P2R7	0.0493	24.6539	−0.3621	0.7294	0.8787
SNP23	Synaptosomal-associated protein 23	O00161	−0.0463	23.8321	0.3596	0.7296	0.8787
RL8	60S ribosomal protein L8	P62917	−0.0395	29.9040	0.3587	0.7302	0.8788
PYRG1	CTP synthase 1	P17812	−0.0319	29.4471	0.3564	0.7318	0.8799
ACOT2	Acyl-CoA thioesterase 2	P49753	−0.0943	23.7950	0.3572	0.7329	0.8799
OTUB1	Otubain-1	Q96FW1	0.0418	27.8668	−0.3530	0.7343	0.8799
CKAP5	Cytoskeleton-associated protein 5	Q14008	−0.0341	26.8698	0.3525	0.7346	0.8799
PFKAL	ATP-dependent 6-phosphofructokinase, liver type	P17858	−0.0616	25.3349	0.3522	0.7349	0.8799
PPME1	Protein phosphatase methylesterase 1	Q9Y570	−0.0439	29.4262	0.3514	0.7354	0.8799
LS14B	Protein LSM14 homolog B	Q9BX40	−0.0668	23.4348	0.3535	0.7355	0.8799
RRS1	Ribosome biogenesis regulatory protein homolog	Q15050	0.0451	25.7611	−0.3510	0.7357	0.8799
RBMX	RNA-binding motif protein, X chromosome	P38159	−0.0571	27.6255	0.3524	0.7363	0.8799
VINC	Vinculin	P18206	−0.0399	31.3228	0.3497	0.7367	0.8799
CD81	CD81 antigen	P60033	−0.0967	24.1612	0.3495	0.7368	0.8799
CBX5	Chromobox protein homolog 5	P45973	−0.1549	26.2339	0.3485	0.7375	0.8802
RL14	60S ribosomal protein L14	P50914	0.0411	29.3352	−0.3443	0.7406	0.8821
TM9S3	Transmembrane 9 superfamily member 3	Q9HD45	0.1809	24.2605	−0.3457	0.7411	0.8821
PSME2	Proteasome activator complex subunit 2	Q9UL46	−0.0555	25.3548	0.3422	0.7421	0.8821
SHPS1	Signal-regulatory protein alpha	P78324	−0.0852	26.0041	0.3401	0.7436	0.8821
RL12	60S ribosomal protein L12	P30050	−0.0393	29.8006	0.3370	0.7458	0.8821
DNJC7	DnaJ homolog subfamily C member 7	Q99615	−0.0590	26.1398	0.3369	0.7459	0.8821
PDE12	2',5'-phosphodiesterase 12	Q6L8Q7	0.0439	25.5693	−0.3366	0.7461	0.8821
SPTC1	Serine palmitoyltransferase 1	O15269	−0.0512	25.0092	0.3365	0.7462	0.8821
MDHM	Malate dehydrogenase, mitochondrial	P40926	0.0400	28.9698	−0.3341	0.7479	0.8821
MRT4	mRNA turnover protein 4 homolog	Q9UKD2	0.0377	26.5486	−0.3339	0.7480	0.8821
RL10	60S ribosomal protein L10a	P27635	0.0644	28.2284	−0.3338	0.7481	0.8821
PSD11	26S proteasome non-ATPase regulatory subunit 11	O00231	0.0410	28.3576	−0.3338	0.7481	0.8821

ATPO	ATP synthase subunit O, mitochondrial	P48047	0.0349	27.7727	−0.3337	0.7482	0.8821
PLAP	Phospholipase A-2-activating protein	Q9Y263	−0.0363	25.0481	0.3326	0.7490	0.8821
SUCB2	Succinyl-CoA synthetase beta-G chain	Q96I99	0.0549	25.7218	−0.3321	0.7493	0.8821
CDK6	Cyclin-dependent kinase 6	Q00534	0.0576	24.1867	−0.3329	0.7503	0.8821
IF4G2	Eukaryotic translation initiation factor 4 gamma 2	P78344	0.0333	26.7249	−0.3304	0.7506	0.8821
TMA16	Translation machinery-associated protein 16	Q96EY4	0.0569	25.0412	−0.3304	0.7506	0.8821
RT18B	28S ribosomal protein S18b, mitochondrial	Q9Y676	−0.0516	24.9936	0.3319	0.7510	0.8821
GIPC1	GAIP C-terminus-interacting protein	O14908	0.1313	23.8705	−0.3308	0.7518	0.8821
EWS	Ewing sarcoma breakpoint region 1 protein	Q01844	−0.0407	25.5769	0.3283	0.7521	0.8821
PUR4	Phosphoribosylformylglycinamidine synthase	O15067	0.0596	27.1380	−0.3276	0.7526	0.8821
SMC3	Structural maintenance of chromosomes protein 3	Q9UQE7	−0.0372	26.5100	0.3266	0.7533	0.8821
HSP13	Heat shock 70 kDa protein 13	P48723	0.0508	23.5142	−0.3311	0.7537	0.8821
SERC	Phosphoserine aminotransferase	Q9Y617	0.0415	26.8439	−0.3260	0.7538	0.8821
CE170	Centrosomal protein of 170 kDa	Q5SW79	−0.1207	25.0819	0.3306	0.7540	0.8821
LAMB1	Laminin subunit beta-1	P07942	0.0395	26.4509	−0.3253	0.7543	0.8821
ECM29	Proteasome-associated protein ECM29 homolog	Q5VYK3	−0.0514	24.1732	0.3249	0.7546	0.8821
GNAI3	Guanine nucleotide-binding protein G(k) subunit alpha	P08754	−0.0725	25.6368	0.3248	0.7546	0.8821
RBMS2	RNA-binding motif, single-stranded-interacting protein 2	Q15434	0.0629	24.5850	−0.3239	0.7553	0.8821
PUR9	Bifunctional purine biosynthesis protein PURH	P31939	−0.0330	29.2907	0.3233	0.7558	0.8821
NADC	Quinolinate phosphoribosyltransferase [decarboxylating])	Q15274	−0.0924	26.9672	0.3250	0.7560	0.8821
PARP1	Poly [ADP-ribose] polymerase 1	P09874	0.0435	27.7507	−0.3228	0.7561	0.8821
RAP2C	Ras-related protein Rap-2c	Q9Y3L5	0.0468	23.6378	−0.3220	0.7567	0.8822
CALM	Calmodulin	P62158	−0.0540	29.2957	0.3204	0.7579	0.8830
EPS8	Epidermal growth factor receptor kinase substrate 8	Q12929	−0.0550	23.3987	0.3190	0.7589	0.8835
RNZ2	Zinc phosphodiesterase ELAC protein 2	Q9BQ52	0.0353	25.1603	−0.3182	0.7594	0.8836
THIL	Acetyl-CoA acetyltransferase, mitochondrial	P24752	0.0406	28.8946	−0.3172	0.7602	0.8839
TENA	Tenascin-C	P24821	0.0434	27.6350	−0.3158	0.7612	0.8844
DRG1	Developmentally-regulated GTP-binding protein 1	Q9Y295	−0.0350	26.5914	0.3142	0.7624	0.8852
DCBD2	Discoidin, CUB and LCCL domain-containing protein 2	Q96PD2	−0.0814	24.2972	0.3131	0.7632	0.8855
VDAC3	Voltage-dependent anion-selective channel protein 3	Q9Y277	−0.0422	27.7625	0.3123	0.7638	0.8856
RS19	40S ribosomal protein S19	P39019	−0.0517	29.1609	0.3112	0.7645	0.8859
TSNAX	Translin-associated protein X	Q99598	0.0422	23.9772	−0.3106	0.7650	0.8859
RL13A	60S ribosomal protein L13a	P40429	0.1330	26.3106	−0.3112	0.7659	0.8860

ECHA	Trifunctional enzyme subunit alpha, mitochondrial	P40939	−0.0345	28.6657	0.3088	0.7663	0.8860
GDS1	Rap1 GTPase-GDP dissociation stimulator 1	P52306	−0.0926	23.4563	0.3102	0.7666	0.8860
PDC10	Programmed cell death protein 10	Q9BUL8	0.0355	24.9721	−0.3089	0.7676	0.8862
COPG1	Coatomer subunit gamma-1	Q9Y678	−0.0441	27.4326	0.3061	0.7682	0.8862
SPB6	Serpin B6	P35237	−0.0308	26.9408	0.3057	0.7686	0.8862
SNX9	Sorting nexin-9	Q9Y5X1	0.0437	24.3015	−0.3052	0.7689	0.8862
FA98A	Protein FAM98A	Q8NCA5	−0.0419	25.7119	0.3040	0.7698	0.8866
GSHB	Glutathione synthetase	P48637	−0.0455	24.2186	0.3051	0.7703	0.8867
G6PD	Glucose-6-phosphate 1-dehydrogenase	P11413	−0.0350	26.5542	0.2971	0.7749	0.8907
DNJA1	DnaJ homolog subfamily A member 1	P31689	0.0375	27.3469	−0.2965	0.7753	0.8907
HPRT	Hypoxanthine-guanine phosphoribosyltransferase	P00492	0.0309	27.6212	−0.2958	0.7758	0.8907
RS11	40S ribosomal protein S11	P62280	−0.0402	28.8590	0.2954	0.7761	0.8907
RAB13	Ras-related protein Rab-13	P51153	0.0672	24.6374	−0.2963	0.7768	0.8907
SYIC	Isoleucyl-tRNA synthetase	P41252	−0.0395	27.9552	0.2943	0.7769	0.8907
PYGB	Glycogen phosphorylase, brain form	P11216	0.0285	27.7567	−0.2929	0.7779	0.8912
ODPB	Pyruvate dehydrogenase E1 component subunit beta, mitochondrial	P11177	−0.0297	26.8283	0.2911	0.7792	0.8921
MOV10	Moloney leukemia virus 10 protein	Q9HCE1	0.0552	23.9584	−0.2889	0.7809	0.8934
SAFB1	Scaffold attachment factor B1	Q15424	0.0508	24.5636	−0.2872	0.7821	0.8938
DX39A	ATP-dependent RNA helicase DDX39A	O00148	−0.0332	25.1886	0.2886	0.7824	0.8938
RPR1A	Regulation of nuclear pre-mRNA domain-containing protein 1A	Q96P16	−0.0719	22.8762	0.2880	0.7828	0.8938
TBB5	Tubulin beta chain	P07437	0.0272	30.1887	−0.2823	0.7857	0.8965
HNRL1	Heterogeneous nuclear ribonucleoprotein U-like protein 1	Q9BUJ2	0.0329	26.6093	−0.2815	0.7863	0.8966
RAC1	Ras-related C3 botulinum toxin substrate 1	P63000	−0.0600	28.1204	0.2764	0.7901	0.9003
TCPD	T-complex protein 1 subunit delta	P50991	−0.0275	30.5312	0.2722	0.7932	0.9032
PURA	Transcriptional activator protein Pur-alpha	Q00577	0.0434	26.1310	−0.2714	0.7937	0.9033
LKHA4	Leukotriene A(4) hydrolase	P09960	−0.0280	28.4144	0.2638	0.7994	0.9084
SYRC	Arginyl-tRNA synthetase	P54136	−0.0272	29.0932	0.2624	0.8004	0.9084
KAD2	Adenylate kinase 2, mitochondrial	P54819	0.0361	26.9079	−0.2623	0.8005	0.9084
DDX46	Probable ATP-dependent RNA helicase DDX46	Q7L014	0.0295	26.6213	−0.2617	0.8009	0.9084
CNPY2	Protein canopy homolog 2	Q9Y2B0	−0.0344	27.0362	0.2617	0.8010	0.9084
NTM1A	N-terminal Xaa-Pro-Lys N-methyltransferase 1	Q9BV86	−0.0711	24.0313	0.2610	0.8014	0.9084
40422	Septin-10	Q9P0V9	0.0489	25.4030	−0.2598	0.8023	0.9085
BICD2	Protein bicaudal D homolog 2	Q8TD16	−0.0749	23.4648	0.2611	0.8026	0.9085
ERF3A	G1 to S phase transition protein 1 homolog	P15170	−0.0363	25.2497	0.2595	0.8037	0.9089
PRKDC	DNA-dependent protein kinase catalytic subunit	P78527	−0.0230	29.4622	0.2576	0.8040	0.9089
RCN1	Reticulocalbin-1	Q15293	0.0804	26.4872	−0.2522	0.8080	0.9128

FA50A	Protein FAM50A	Q14320	0.0362	25.4140	−0.2531	0.8084	0.9128
NUP93	Nucleoporin Nup93	Q8N1F7	−0.0251	27.5189	0.2484	0.8108	0.9144
ERP29	Endoplasmic reticulum resident protein 29	P30040	0.0244	28.2209	−0.2482	0.8110	0.9144
MBOA7	Membrane-bound O-acyltransferase domain-containing protein 7	Q96N66	−0.0602	23.9328	0.2446	0.8147	0.9176
SNUT1	Squamous cell carcinoma antigen recognized by T-cells 1	O43290	−0.0363	25.1900	0.2444	0.8149	0.9176
PRS8	Proteasome 26S subunit ATPase 5	P62195	−0.0223	27.9154	0.2421	0.8155	0.9177
RL6	60S ribosomal protein L6	Q02878	−0.0240	30.9047	0.2368	0.8194	0.9194
THOC1	THO complex subunit 1	Q96FV9	0.0348	23.1301	−0.2378	0.8197	0.9194
PLOD3	Procollagen-lysine,2-oxoglutarate 5-dioxygenase 3	O60568	0.0448	24.3449	−0.2396	0.8199	0.9194
MOT1	Solute carrier family 16 member 1	P53985	−0.0402	24.8716	0.2351	0.8207	0.9194
SO4C1	Solute carrier organic anion transporter family member 4C1	Q6ZQN7	−0.0487	23.0040	0.2383	0.8209	0.9194
PDCD5	Programmed cell death protein 5	O14737	0.0425	26.9990	−0.2345	0.8212	0.9194
MMS19	MMS19 nucleotide excision repair protein homolog	Q96T76	−0.0379	23.6528	0.2352	0.8217	0.9194
STAU1	Double-stranded RNA-binding protein Staufen homolog 1	O95793	−0.0242	27.3465	0.2336	0.8218	0.9194
PARK7	Parkinson disease protein 7	Q99497	0.0264	30.1705	−0.2330	0.8223	0.9194
DHB12	17-beta-hydroxysteroid dehydrogenase 12	Q53GQ0	−0.0423	24.8017	0.2343	0.8224	0.9194
BAP31	B-cell receptor-associated protein 31	P51572	0.0329	26.6629	−0.2307	0.8240	0.9203
LAR4B	La-related protein 4B	Q92615	−0.0386	23.3430	0.2305	0.8242	0.9203
GAGE3	G antigen 3	Q13067	0.0625	28.0949	−0.2281	0.8260	0.9216
CHD4	Chromodomain-helicase-DNA-binding protein 4	Q14839	0.0428	24.7153	−0.2286	0.8266	0.9217
TCTP	Translationally-controlled tumor protein	P13693	−0.0498	26.5754	0.2262	0.8274	0.9220
MYOF	Myoferlin	Q9NZM1	−0.0236	29.5330	0.2250	0.8283	0.9221
IN35	Interferon-induced 35 kDa protein	P80217	−0.0476	24.7006	0.2241	0.8289	0.9221
WDR74	WD repeat-containing protein 74	Q6RFH5	0.0839	23.0169	−0.2271	0.8291	0.9221
ULA1	NEDD8-activating enzyme E1 regulatory subunit	Q13564	−0.0463	24.8768	0.2227	0.8300	0.9223
TP53B	Tumor suppressor p53-binding protein 1	Q12888	0.0339	24.8525	−0.2215	0.8309	0.9223
HEM3	Hydroxymethylbilane synthase	P08397	−0.0308	24.3647	0.2215	0.8309	0.9223
HNRPM	Heterogeneous nuclear ribonucleoprotein M	P52272	0.0243	29.8277	−0.2193	0.8325	0.9235
UBR4	E3 ubiquitin-protein ligase UBR4	Q5T4S7	−0.0256	27.0806	0.2172	0.8341	0.9241
DDX54	ATP-dependent RNA helicase DDX54	Q8TDD1	0.0523	25.5269	−0.2157	0.8352	0.9241
GMFB	Glia maturation factor beta	P60983	−0.0801	25.4832	0.2156	0.8353	0.9241
PRDX1	Peroxiredoxin-1	Q06830	0.0200	32.0959	−0.2140	0.8365	0.9241
AIMP2	Aminoacyl tRNA synthase complex-interacting multifunctional protein 2	Q13155	−0.0278	26.1732	0.2140	0.8365	0.9241
RL22	60S ribosomal protein L22	P35268	0.0245	29.1745	−0.2140	0.8365	0.9241
NOG2	Nucleolar GTP-binding protein 2	Q13823	0.0545	23.8272	−0.2148	0.8369	0.9241
HNRPF	Heterogeneous nuclear ribonucleoprotein F	P52597	0.0288	27.4550	−0.2127	0.8375	0.9241

F136A	Protein FAM136A	Q96C01	0.0675	24.8661	−0.2118	0.8382	0.9241
MAP1B	Microtubule-associated protein 1B	P46821	0.0227	27.7460	−0.2115	0.8384	0.9241
GALT7	N-acetylgalactosaminyltransferase 7	Q86SF2	−0.0288	24.4842	0.2093	0.8401	0.9253
SRP68	Signal recognition particle subunit SRP68	Q9UHB9	0.0204	26.6218	−0.2048	0.8434	0.9282
RS24	40S ribosomal protein S24	P62847	0.1076	27.1645	−0.2039	0.8442	0.9282
DLDH	Dihydrolipoyl dehydrogenase, mitochondrial	P09622	−0.0238	25.5598	0.2037	0.8443	0.9282
EMC3	ER membrane protein complex subunit 3	Q9P0I2	0.0627	23.3856	−0.2030	0.8457	0.9291
RM38	39S ribosomal protein L38, mitochondrial	Q96DV4	0.0334	24.4460	−0.2006	0.8466	0.9294
ILK	Integrin-linked protein kinase	Q13418	0.0422	25.7675	−0.2000	0.8470	0.9294
RL18A	60S ribosomal protein L18a	Q02543	−0.0241	28.5491	0.1986	0.8481	0.9300
RM53	39S ribosomal protein L53, mitochondrial	Q96EL3	−0.0460	24.9319	0.1950	0.8529	0.9337
HM13	Minor histocompatibility antigen H13	Q8TCT9	−0.0325	26.7604	0.1922	0.8529	0.9337
EIF3G	Eukaryotic translation initiation factor 3 subunit G	O75821	−0.0267	27.8041	0.1920	0.8531	0.9337
DNJC8	DnaJ homolog subfamily C member 8	O75937	−0.0530	25.5873	0.1886	0.8557	0.9359
CAP1	Adenylyl cyclase-associated protein 1	Q01518	0.0210	30.5104	−0.1863	0.8574	0.9368
RBM4	RNA-binding motif protein 4	Q9BWF3	−0.0232	26.6913	0.1860	0.8576	0.9368
SNAG	N-ethylmaleimide-sensitive factor attachment protein gamma	Q99747	−0.0305	25.0444	0.1849	0.8585	0.9370
DC1L1	Cytoplasmic dynein 1 light intermediate chain 1	Q9Y6G9	0.0233	26.1676	−0.1843	0.8589	0.9370
ACSL4	Long-chain-fatty-acid-CoA ligase 4	O60488	0.0201	25.9589	−0.1822	0.8605	0.9376
RAVR1	Ribonucleoprotein PTB-binding 1	Q8IY67	0.0207	23.9095	−0.1825	0.8611	0.9376
BZW1	Basic leucine zipper and W2 domain-containing protein 1	Q7L1Q6	−0.0436	25.9547	0.1811	0.8613	0.9376
RL3	60S ribosomal protein L3	P39023	−0.0276	30.2146	0.1806	0.8617	0.9376
UN45A	Protein unc-45 homolog A	Q9H3U1	0.0437	24.6771	−0.1800	0.8622	0.9376
LRC59	Leucine-rich repeat-containing protein 59	Q96AG4	−0.0200	28.8110	0.1778	0.8638	0.9380
EIF3B	Eukaryotic translation initiation factor 3 subunit B	P55884	0.0192	28.2173	−0.1777	0.8639	0.9380
TPM3	Tropomyosin alpha-3 chain	P06753	0.0257	29.2269	−0.1774	0.8641	0.9380
SYMPK	Symplekin	Q92797	−0.0324	23.4992	0.1746	0.8662	0.9396
PUR6	Multifunctional protein ADE2	P22234	−0.0177	30.2809	0.1732	0.8673	0.9399
VPS35	Vesicle protein sorting 35	Q96QK1	0.0213	28.3465	−0.1712	0.8688	0.9399
ERLN2	Endoplasmic reticulum lipid raft-associated protein 2	O94905	−0.0352	23.8646	0.1710	0.8690	0.9399
ELP1	IkappaB kinase complex-associated protein	O95163	−0.0302	23.4921	0.1718	0.8692	0.9399
KHDR1	KH domain-containing, RNA-binding, signal transduction-associated protein 1	Q07666	−0.0473	27.0801	0.1700	0.8698	0.9399
RS26	40S ribosomal protein S26	P62854	0.0190	28.0459	−0.1697	0.8699	0.9399
DUT	Deoxyuridine 5'-triphosphate nucleotidohydrolase, mitochondrial	P33316	−0.0269	28.5745	0.1692	0.8703	0.9399

STK26	Serine/threonine-protein kinase 26	Q9P289	−0.0164	24.7299	0.1667	0.8722	0.9414
NADAP	Solute carrier family 4 anion exchanger member 1 adapter protein	Q9BWU0	−0.0291	24.1132	0.1668	0.8729	0.9415
RLA2	60S acidic ribosomal protein P2	P05387	0.0187	30.4320	−0.1629	0.8751	0.9433
HUMMR	Mitochondria-localized glutamic acid-rich protein	Q8TDB4	−0.0266	25.6753	0.1619	0.8766	0.9443
EPN4	Clathrin interactor 1	Q14677	0.0267	26.2514	−0.1581	0.8788	0.9461
GRP75	Heat shock 70 kDa protein 9	P38646	−0.0147	30.9489	0.1550	0.8811	0.9480
TCPE	T-complex protein 1 subunit epsilon	P48643	−0.0162	30.2092	0.1534	0.8824	0.9481
IF2B3	Insulin-like growth factor 2 mRNA-binding protein 3	O00425	−0.0153	27.2341	0.1526	0.8830	0.9481
UBF1	Upstream-binding factor 1	P17480	0.0344	25.3549	−0.1506	0.8844	0.9481
EPHA2	Ephrin type-A receptor 2	P29317	0.0211	26.2180	−0.1501	0.8848	0.9481
GSTO1	Glutathione S-transferase omega-1	P78417	0.0199	27.4691	−0.1493	0.8854	0.9481
SC24C	Protein transport protein Sec24C	P53992	−0.0176	26.0600	0.1485	0.8860	0.9481
DCTN1	Dynactin subunit 1	Q14203	−0.0157	27.5065	0.1484	0.8861	0.9481
XRCC5	X-ray repair cross-complementing protein 5	P13010	0.0156	29.2181	−0.1468	0.8874	0.9481
PCH2	Thyroid receptor-interacting protein 13	Q15645	0.0144	26.1241	−0.1460	0.8880	0.9481
PQBP1	Polyglutamine-binding protein 1	O60828	0.0162	25.5105	−0.1456	0.8883	0.9481
RU17	U1 small nuclear ribonucleoprotein 70 kDa	P08621	−0.0209	26.7698	0.1453	0.8885	0.9481
EEA1	Early endosome antigen 1	Q15075	−0.0167	28.1213	0.1447	0.8889	0.9481
TLN1	Talin-1	Q9Y490	−0.0140	29.1213	0.1437	0.8897	0.9481
MVP	Major vault protein	Q14764	0.0161	29.8891	−0.1434	0.8899	0.9481
CHCH2	Coiled-coil-helix-coiled-coil-helix domain-containing protein 2	Q9Y6H1	−0.0272	25.5131	0.1442	0.8900	0.9481
TOM40	Translocase of outer membrane 40 kDa subunit homolog	O96008	0.0207	26.8343	−0.1433	0.8900	0.9481
PESC	Pescadillo homolog	O00541	−0.0256	25.3737	0.1392	0.8931	0.9491
ROCK1	Rho-associated, coiled-coil-containing protein kinase 1	Q13464	−0.0401	24.3871	0.1388	0.8934	0.9491
VAT1	Synaptic vesicle membrane protein VAT-1 homolog	Q99536	−0.0151	28.3379	0.1381	0.8940	0.9491
RB11B	Ras-related protein Rab-11B	Q15907	0.0139	28.1003	−0.1379	0.8941	0.9491
PAGE2	Prostate-associated gene 2 protein	Q7Z2X7	−0.0133	28.5273	0.1379	0.8941	0.9491
SSF1	Peter Pan homolog	Q9NQ55	0.0181	23.2022	−0.1395	0.8943	0.9491
TRM61	tRNA (adenine(58)-N(1))-methyltransferase catalytic subunit TRMT61A	Q96FX7	−0.0375	23.2039	0.1348	0.8965	0.9507
ODP2	Dihydrolipoamide acetyltransferase component of pyruvate dehydrogenase complex	P10515	−0.0149	27.2550	0.1342	0.8969	0.9507
LRBA	Lipopolysaccharide-responsive and beige-like anchor protein	P50851	−0.0189	24.8683	0.1353	0.8975	0.9507
ILKAP	Integrin-linked kinase-associated serine/threonine phosphatase 2C	Q9H0C8	−0.0210	23.4861	0.1344	0.8982	0.9509

SH3G1	SH3 domain-containing GRB2-like protein 1	Q99961	0.0132	25.2358	−0.1311	0.8993	0.9512
HECD1	E3 ubiquitin-protein ligase HECTD1	Q9ULT8	−0.0249	23.4631	0.1314	0.8997	0.9512
AP3D1	Adaptor-related protein complex 3 subunit delta-1	O14617	0.0461	23.9979	−0.1294	0.9012	0.9523
NU155	Nucleoporin Nup155	O75694	−0.0137	24.7349	0.1260	0.9032	0.9539
CLIC1	Chloride intracellular channel protein 1	O00299	−0.0123	30.6734	0.1245	0.9044	0.9545
SRSF1	Serine/arginine-rich splicing factor 1	Q07955	0.0139	27.4847	−0.1233	0.9052	0.9545
HEAT1	HEAT repeat-containing protein 1	Q9H583	−0.0294	25.1995	0.1229	0.9055	0.9545
HNRPK	Heterogeneous nuclear ribonucleoprotein K	P61978	−0.0153	30.7816	0.1208	0.9072	0.9557
ZW10	Centromere/kinetochore protein zw10 homolog	O43264	−0.0118	24.0596	0.1186	0.9089	0.9566
KDM2A	Lysine-specific demethylase 2A	Q9Y2K7	−0.0322	23.6520	0.1198	0.9092	0.9566
TBCA	Tubulin-specific chaperone A	O75347	0.0146	28.7631	−0.1161	0.9108	0.9575
SAM50	Sorting and assembly machinery component 50 homolog	Q9Y512	0.0265	23.5241	−0.1162	0.9112	0.9575
NCKP1	Nck-associated protein 1	Q9Y2A7	−0.0106	25.0638	0.1137	0.9126	0.9581
UBE2N	Ubiquitin-conjugating enzyme E2 N	P61088	−0.0135	28.0005	0.1132	0.9130	0.9581
RAB10	Ras-related protein Rab-10	P61026	−0.0283	25.3272	0.1120	0.9139	0.9581
CIR1A	Cirhin	Q969X6	−0.0223	24.3466	0.1119	0.9140	0.9581
AKA12	A-kinase anchor protein 12	Q02952	0.0120	27.9721	−0.1110	0.9146	0.9582
PADI2	Protein-arginine deiminase type-2	Q9Y2J8	0.0213	26.4974	−0.1057	0.9187	0.9619
NPL4	Nuclear protein localization protein 4 homolog	Q8TAT6	−0.0095	26.6132	0.1014	0.9220	0.9648
SGTA	Small glutamine-rich tetratricopeptide repeat-containing protein alpha	O43765	−0.0126	26.4359	0.1007	0.9226	0.9648
NIF3L	NIF3-like protein 1	Q9GZT8	−0.0138	22.9266	0.1002	0.9240	0.9656
HXK1	Hexokinase-1	P19367	0.0130	24.2525	−0.0987	0.9245	0.9656
SAE2	Ubiquitin-like modifier-activating enzyme 2	Q9UBT2	−0.0115	26.8660	0.0952	0.9268	0.9674
TPIS	Triosephosphate isomerase	P60174	0.0098	31.1575	−0.0922	0.9291	0.9687
RRAS2	Ras-related protein R-Ras2	P62070	0.0148	25.4447	−0.0927	0.9291	0.9687
HAP28	PDGF-associated protein	Q13442	0.0108	26.2844	−0.0856	0.9341	0.9727
TAGL2	Transgelin-2	P37802	0.0085	29.9114	−0.0854	0.9343	0.9727
PFKAP	ATP-dependent 6-phosphofructokinase, platelet type	Q01813	−0.0107	29.5856	0.0848	0.9347	0.9727
SART3	Squamous cell carcinoma antigen recognized by T-cells 3	Q15020	−0.0152	24.5994	0.0835	0.9358	0.9730
IF2A	Eukaryotic translation initiation factor 2 subunit 1	P05198	−0.0085	28.6433	0.0830	0.9362	0.9730
SAHH2	S-adenosylhomocysteine hydrolase-like protein 1	O43865	0.0113	25.9384	−0.0814	0.9377	0.9731
PSIP1	PC4 and SFRS1-interacting protein	O75475	−0.0191	24.6604	0.0810	0.9377	0.9731
CSN3	COP9 signalosome complex subunit 3	Q9UNS2	−0.0121	25.2925	0.0807	0.9379	0.9731
CARM1	Coactivator-associated arginine methyltransferase 1	Q86X55	−0.0127	23.5781	0.0774	0.9412	0.9755
GLOD4	Glyoxalase domain-containing protein 4	Q9HC38	0.0090	26.5124	−0.0762	0.9413	0.9755
GMPPB	GDP-mannose pyrophosphorylase B	Q9Y5P6	−0.0078	25.8619	0.0707	0.9456	0.9786

MYPT1	Protein phosphatase 1 regulatory subunit 12A	O14974	0.0092	25.1412	−0.0692	0.9467	0.9786
LG3BP	Lectin galactoside-binding soluble 3-binding protein	Q08380	0.0104	24.6825	−0.0690	0.9469	0.9786
CSN8	COP9 signalosome complex subunit 8	Q99627	−0.0171	24.9079	0.0688	0.9470	0.9786
MCM4	DNA replication licensing factor MCM4	P33991	0.0090	27.7978	−0.0660	0.9492	0.9786
UBC12	Ubiquitin-conjugating enzyme E2 M	P61081	0.0099	26.1980	−0.0658	0.9493	0.9786
ACLY	ATP-citrate synthase	P53396	0.0059	29.3298	−0.0650	0.9499	0.9786
CLIC4	Chloride intracellular channel protein 4	Q9Y696	0.0124	27.3356	−0.0650	0.9500	0.9786
CATA	Catalase	P04040	−0.0067	26.1095	0.0648	0.9501	0.9786
UMPS	Uridine 5'-monophosphate synthase	P11172	0.0097	25.2189	−0.0645	0.9503	0.9786
38961	Septin-6	Q14141	−0.0094	25.1434	0.0641	0.9507	0.9786
IF2B2	Insulin-like growth factor 2 mRNA-binding protein 2	Q9Y6M1	−0.0079	25.7658	0.0628	0.9516	0.9787
EF1A1	Eukaryotic elongation factor 1 A-1	P68104	0.0070	33.6304	−0.0625	0.9519	0.9787
PRS6A	Proteasome 26S subunit ATPase 3	P17980	0.0057	28.2582	−0.0600	0.9538	0.9792
SRPR	Signal recognition particle receptor subunit alpha	P08240	−0.0062	26.7418	0.0592	0.9544	0.9792
NUCKS	Nuclear ubiquitous casein and cyclin-dependent kinase substrate 1	Q9H1E3	−0.0148	23.9615	0.0588	0.9547	0.9792
STT3A	Dolichyl-diphosphooligosaccharide--protein glycosyltransferase subunit STT3A	P46977	−0.0066	26.4398	0.0575	0.9557	0.9792
RL34	60S ribosomal protein L34	P49207	0.0105	27.4716	−0.0577	0.9558	0.9792
PSMD1	26S proteasome non-ATPase regulatory subunit 1	Q99460	0.0055	28.7802	−0.0568	0.9562	0.9792
PTRF	Polymerase I and transcript release factor	Q6NZI2	−0.0072	29.3516	0.0566	0.9564	0.9792
SPSY	Spermine synthase	P52788	−0.0069	28.2898	0.0525	0.9596	0.9819
IGBP1	Immunoglobulin-binding protein 1	P78318	−0.0101	24.8114	0.0497	0.9617	0.9834
LETM1	Leucine zipper-EF-hand-containing transmembrane protein 1	O95202	0.0056	27.8897	−0.0486	0.9626	0.9834
ODPA	Pyruvate dehydrogenase E1 component subunit alpha, somatic form, mitochondrial	P08559	−0.0080	25.9735	0.0482	0.9629	0.9834
RS12	40S ribosomal protein S12	P25398	0.0073	29.9067	−0.0476	0.9633	0.9834
MDHC	Malate dehydrogenase, cytoplasmic	P40925	−0.0056	28.7744	0.0443	0.9658	0.9848
HGS	Hepatocyte growth factor-regulated tyrosine kinase substrate	O14964	−0.0063	26.1623	0.0436	0.9664	0.9848
37500	Septin-2	Q15019	0.0052	28.1065	−0.0431	0.9668	0.9848
MYL6	Myosin light polypeptide 6	P60660	0.0052	29.7005	−0.0427	0.9671	0.9848
GGCT	Gamma-glutamylcyclotransferase	O75223	−0.0085	24.8762	0.0423	0.9676	0.9848
IF5A1	Eukaryotic initiation factor 5A isoform 1	P63241	0.0044	28.2475	−0.0401	0.9691	0.9855
SF01	Splicing factor 1	Q15637	0.0099	26.1085	−0.0397	0.9695	0.9855
ATG3	Autophagy-related protein 3	Q9NT62	0.0049	24.2008	−0.0374	0.9716	0.9871
NUCB1	Nucleobindin-1	Q02818	0.0065	24.8985	−0.0362	0.9723	0.9873
RBM10	RNA-binding motif protein 10	P98175	−0.0048	24.7372	0.0331	0.9745	0.9885
DDAH2	Dimethylarginine dimethylaminohydrolase 2	O95865	0.0049	24.7538	−0.0329	0.9746	0.9885

ZFR	Zinc finger RNA-binding protein	Q96KR1	0.0041	24.6739	−0.0308	0.9763	0.9890
ANM5	Protein arginine N-methyltransferase 5	O14744	0.0041	27.9297	−0.0308	0.9763	0.9890
MCM7	DNA replication licensing factor MCM7	P33993	−0.0035	28.0301	0.0278	0.9785	0.9895
PSMD3	26S proteasome non-ATPase regulatory subunit 3	O43242	0.0030	27.9393	−0.0276	0.9787	0.9895
ROA3	Heterogeneous nuclear ribonucleoprotein A3	P51991	0.0031	28.1995	−0.0272	0.9791	0.9895
RAD50	DNA repair protein RAD50	Q92878	−0.0050	26.6405	0.0268	0.9793	0.9895
RS3	40S ribosomal protein S3	P23396	−0.0026	30.5390	0.0264	0.9797	0.9895
AN32A	Acidic leucine-rich nuclear phosphoprotein 32 family member A	P39687	−0.0042	26.8469	0.0244	0.9813	0.9903
RS7	40S ribosomal protein S7	P62081	0.0030	28.7745	−0.0238	0.9817	0.9903
SRP09	Signal recognition particle 9 kDa protein	P49458	0.0032	26.4072	−0.0217	0.9833	0.9914
APEX1	DNA-(apurinic or apyrimidinic site) lyase	P27695	−0.0020	28.1985	0.0205	0.9842	0.9917
QOR	Quinone oxidoreductase	Q08257	0.0020	28.2702	−0.0179	0.9862	0.9932
RPAC2	DNA-directed RNA polymerases I and III subunit RPAC2	Q9Y2S0	−0.0038	24.1414	0.0169	0.9871	0.9935
YTHD3	YTH domain-containing family protein 3	Q7Z739	−0.0011	23.8633	0.0090	0.9931	0.9985
DBLOH	Second mitochondria-derived activator of caspase	Q9NR28	0.0032	25.1277	−0.0081	0.9938	0.9985
CSRP1	Cysteine and glycine-rich protein 1	P21291	0.0022	25.8455	−0.0073	0.9944	0.9985
LEG1	Galectin-1	P09382	0.0006	30.1747	−0.0057	0.9956	0.9985
PININ	Pinin	Q9H307	−0.0005	25.1029	0.0039	0.9970	0.9985
PDLI7	Protein enigma	Q9NR12	−0.0004	25.5047	0.0038	0.9971	0.9985
ENOA	Alpha-enolase	P06733	−0.0003	32.7067	0.0034	0.9974	0.9985
RFA2	Replication protein A 32 kDa subunit	P15927	0.0008	25.5289	−0.0030	0.9977	0.9985
PBDC1	Polysaccharide biosynthesis domain-containing protein 1	Q9BVG4	−0.0003	25.2787	0.0027	0.9979	0.9985
LMAN2	Vesicular integral-membrane protein VIP36	Q12907	−0.0004	25.9042	0.0026	0.9980	0.9985
RAGP1	Ran GTPase-activating protein 1 (RanGAP1)	P46060	0.0002	28.4556	−0.0017	0.9987	0.9987

Table shows global protein changes upon CAS depletion in HLE cells adjusted to the control siRNA assessed by qMS. Yellow indicates adjusted p-value < 0.005.
